# Association of major depression, schizophrenia and bipolar disorder with thyroid cancer: a bidirectional two-sample mendelian randomized study

**DOI:** 10.1186/s12888-024-05682-7

**Published:** 2024-04-09

**Authors:** Rongliang Qiu, Huihui Lin, Hongzhan Jiang, Jiali Shen, Jiaxi He, Jinbo Fu

**Affiliations:** 1 The School of Clinical Medicine, Fujian Medical University, Fuzhou, 350122 China; 2 Department of General Surgery, Zhongshan Hospital of Xiamen University, Xiamen, 361004 China; 3 School of Nursing, Fujian University of Traditional Chinese Medicine, Fuzhou, 350122 China; 4 School of Medicine, Xiamen University, Xiamen, 361004 China

**Keywords:** Major depressive disease, Schizophrenia, Bipolar disorder, Mendelian randomization, Thyroid cancer

## Abstract

**Background:**

Major depressive disease (MDD), schizophrenia (SCZ), and bipolar disorder (BD) are common psychiatric disorders, and their relationship with thyroid cancer has been of great interest. This study aimed to investigate the potential causal effects of MDD, SCZ, BD, and thyroid cancer.

**Methods:**

We used publicly available summary statistics from large-scale genome-wide association studies to select genetic variant loci associated with MDD, SCZ, BD, and thyroid cancer as instrumental variables (IVs), which were quality controlled and clustered. Additionally, we used three Mendelian randomization (MR) methods, inverse variance weighted (IVW), MR–Egger regression and weighted median estimator (WME) methods, to estimate the bidirectional causal relationship between psychiatric disorders and thyroid cancer. In addition, we performed heterogeneity and multivariate tests to verify the validity of the IVs.

**Results:**

We used two-sample bidirectional MR analysis to determine whether there was a positive causal association between MDD and thyroid cancer risk. The results of the IVW analysis (OR = 3.956 95% CI = 1.177–13.299; *P* = 0.026) and the WME method (OR = 5.563 95% CI = 0.998–31.008; *P* = 0.050) confirmed that MDD may increase the risk of thyroid cancer. Additionally, our study revealed a correlation between genetic susceptibility to SCZ and thyroid cancer (OR = 1.532 95% CI = 1.123–2.088; *P* = 0.007). The results of the WME method analysis based on the median estimate (OR = 1.599 95% CI = 1.014–2.521; *P* = 0.043) also suggested that SCZ may increase the risk of thyroid cancer. Furthermore, our study did not find a causal relationship between BD and thyroid cancer incidence. In addition, the results of reverse MR analysis showed no significant causal relationships between thyroid cancer and MDD, SCZ, or BD (*P* > 0.05), ruling out the possibility of reverse causality.

**Conclusions:**

This MR method analysis provides new evidence that MDD and SCZ may be positively associated with thyroid cancer risk while also revealing a correlation between BD and thyroid cancer. These results may have important implications for public health policy and clinical practice. Future studies will help elucidate the biological mechanisms of these associations and potential confounders.

**Supplementary Information:**

The online version contains supplementary material available at 10.1186/s12888-024-05682-7.

## Background

Cancer is recognized as a disease that poses a serious threat to human health, and it has been reported that in the United States alone, more than 609,360 people are expected to lose their lives to cancer in 2022 [1]. However, the mechanisms underlying the development of most cancers are still not fully understood, which has led to delays in the diagnosis and treatment of cancers, contributing to the increasing incidence and mortality of cancer worldwide. Thyroid cancer is one of the most common endocrine tumours, and its incidence has been steadily increasing worldwide over the last three decades due to the widespread use of diagnostic imaging techniques and ultrasound-guided fine needle aspiration (US-FNA) [2, 3]. Despite the continued increase in the incidence of thyroid cancer, its mortality trend has remained relatively stable. Risk factors for thyroid cancer include metabolic syndrome (including diabetes mellitus, hypertension, obesity, etc.), poor lifestyle habits, and environmental pollution, but these factors do not fully explain the mechanism of thyroid cancer development. Therefore, identifying other potentially modifiable risk factors, such as psychiatric disorders, is important for the prevention and treatment of thyroid cancer.

Major depressive disease (MDD), schizophrenia (SCZ), and bipolar disorder (BD) are all serious psychiatric disorders that overlap genetically and clinically, suggesting that they may share common aetiological mechanisms [4]. The results of a study suggest that quantitative changes in plasma lipids affect several individual characteristics, including those affected by serious psychiatric disorders (MDD, SCZ and BD) [5]. Moreover, clinical studies have shown that patients with MDD, SCZ and BD have altered Homer1a levels in specific regions and cell types of the brain. A growing body of research confirms the close connection between these three disorders [6]. MDD is ranked by the World Health Organization as one of the most burdensome diseases in the world; it seriously damages people’s physical and mental health and is associated with a variety of endocrine disorders, such as hypothyroidism and hyperthyroidism [7–11]. Studies have shown that patients with hyperthyroidism and hypothyroidism differ in the presentation of depressive symptoms and disorders [12, 13]. Specifically, hyperthyroidism was associated with more depressive symptoms (e.g., insomnia and weight loss) [14], whereas hypothyroidism was associated with fewer depressive symptoms (e.g., energy deficit and fatigue) [15]. In addition, individuals with hyperthyroidism have a higher incidence of MDD [13]. The relationship between depression and cancer has long been of interest, and some observational studies have suggested that depression may be an important risk factor for cancer [16]. A cross-sectional study from Korea revealed a 5.6% incidence of depression in thyroid cancer patients [17]. Another study from Germany showed that cancer patients were five times more likely to be depressed than was the general population, and thyroid cancer patients with a detectable high burden of depressive symptoms were 9.3 times more likely to be depressed than was the general population [18]. However, despite observational studies revealing a correlation between MDD and thyroid cancer, the relationship between MDD and thyroid cancer has not been systematically explored.

SCZ is a chronic psychiatric disorder accompanied by inconsistent behavioural and cognitive symptoms and has profound effects on both individuals and society. More than 50% of those diagnosed have intermittent and chronic psychiatric problems [19]. This results in a particularly high risk of disengagement from the labour market, with employment rates ranging from 10 to 30%, unemployment rates as high as 89–95%, and a 15–20 year reduction in life expectancy [20, 21]. There is growing evidence that thyroid function may be altered in patients with SCZ, but the results of observational studies have been inconsistent [22, 23]. In addition, the role of the thyroid gland in the pathophysiology of SCZ is poorly understood, and the relationship between thyroid disorders and SCZ is unclear.

SCZ and BD are considered part of the psychiatric continuum and share similar clinical features. BD is a chronic, disabling illness and a major contributor to the global burden of disease. BD can cause mood swings ranging from depression to mania. Patients exhibit fluctuations during the course of the illness, with some patients experiencing episodes only every few years, while others experience episodes almost continuously. A large body of evidence confirms the association between abnormal thyroid hormone levels and different psychopathological conditions, triggering neuropsychiatric symptoms [24]. However, observational studies may find an association between psychiatric disorders and thyroid disorders, but confounding factors and reverse causality cannot be excluded.

To explore the causal association between psychiatric disorders (MDD, SCZ and BD) and thyroid cancer risk, we used a two-sample bidirectional Mendelian randomization (MR) study. MR studies [25] use genetic variation as an instrumental variable closely related to the exposure of interest to explore the causal effect between the exposure and the outcome, thereby improving the reliability of causal inference. Because of the random segregation of alleles at the meiotic stage and the stochastic nature of germline genetic variation at fertilization, MR analyses can avoid confounding factors and reverse causation. In this study, we utilized a two-sample bidirectional MR approach to explore the associations between MDD, SCZ, BD, and thyroid cancer based on statistically pooled data from a genome-wide association study (GWAS). This study aimed to gain insights into the potential link between psychiatric disorders and thyroid cancer and to provide new perspectives and insights for the prevention and treatment of thyroid cancer.

## Methods

**Fig. 1:**
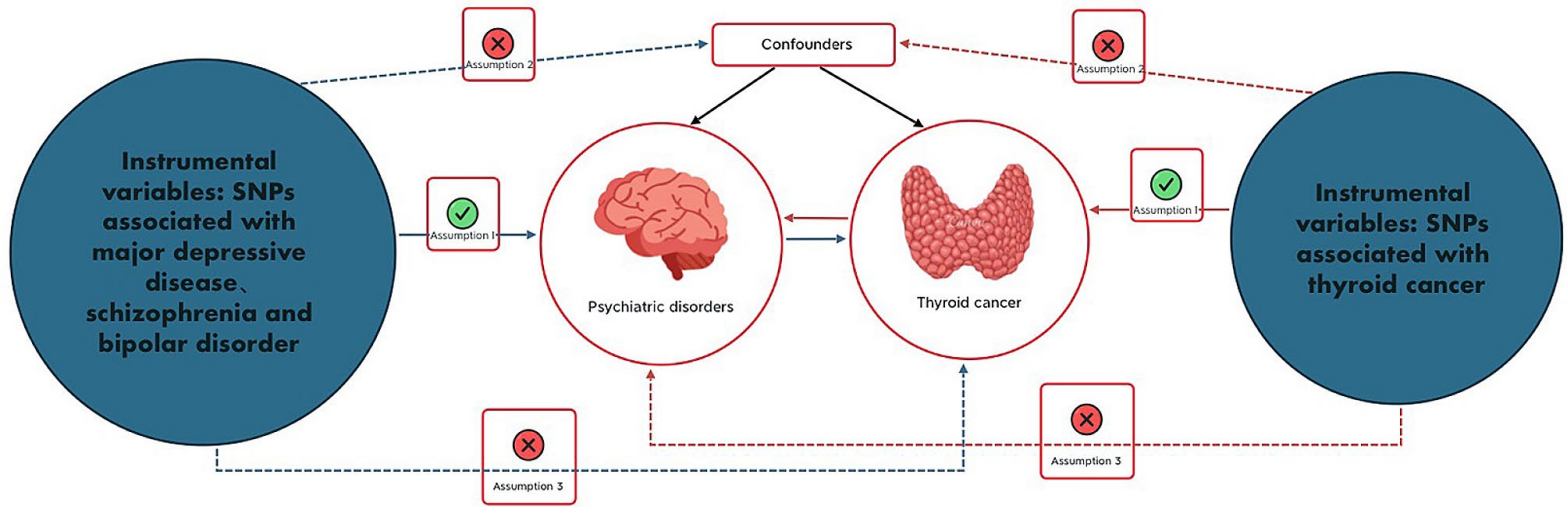
Flowchart of the design of a Mendelian randomized study of the causal association between psychiatric disorders and thyroid cancer. Blue solid lines represent associations between instrumental variables (SNPs) and exposure and between exposure and outcome. The red solid line represents reverse causality. psychiatric disorders include major depression, schizophrenia, and bipolar disorder

This study is an analysis of previously collected and published public data, including statistical aggregations related to MDD, SCZ, BD, and thyroid cancer, from large public GWASs. Due to the source and nature of the data, no additional ethical review or informed consent was required for this study. Two-sample bidirectional MR analyses were used to assess the causal relationship between psychiatric disorders (MDD, SCZ, BD) and thyroid cancer. We chose psychiatric disorders (MDD, SCZ, BD) as the exposure factor and thyroid cancer as the outcome indicator. Moreover, we conducted a reverse two-sample MR analysis with thyroid cancer as an exposure factor and psychiatric disorders (MDD, SCZ, BD) as an outcome indicator. A flow chart of the MR research design constructed according to this paper is shown in Fig. 1.

### Data sources for patients with major depression, schizophrenia, bipolar disorder, or thyroid cancer

The summary-level dataset used for GWASs for MDD in this study was obtained from a meta-analysis of GWAS data conducted by Howard et al. [26]. It comprises three large-scale GWASs, the Psychiatric Genomics Consortium (PGC), the UK Biobank, and 23andme. Of the three GWASs, only the UK Biobank and the PGC publish summary statistics on genetic variation. The dataset included 500,199 European subjects, including 170,756 cases and 329,443 controls. In the UK Biobank, Howard et al. used a broad definition of depression and asked participants if they reported neurological, anxiety, tension, or depression symptoms to their general practitioner or psychiatrist. At the PGC, Wray et al. diagnosed depression in participants according to international consensus diagnostic criteria (DSM-IV, ICD-9, or ICD-10). See Table 1 for details.

**Table 1:** Genetic summary data sources for psychiatric disorders and thyroid cancer

Trait	Sample size	Population	ncase	ncontrol	Sex	Year	PMID
Major depressive disease	500,199	European	170,756	329,443	Males and Females	2019	30,718,901
Schizophrenia	320,404	European, East Asian, African American and Latino	76,755	243,649	Males and Females	2022	35,396,580
Bipolar disorder	51,710	European	20,352	31,358	Males and Females	2019	31,043,756
Thyroid cancer	1080	European	649	431	Males and Females	2013	23,894,154

Statistical summary data for SCZ and BD were obtained from the most recent PGC’s GWAS summary statistics. The data for SCZ [27] are based on a major meta-analysis of multiple groups, including Europeans, East Asians, African Americans, and Latinos, including 76,755 cases and 243,649 control participants. BD [28] was based on a summary analysis of European ancestry and included 20,352 cases and 31,358 control participants. See Table 1 for detailed information.

To perform two-sample bidirectional analyses, we used independent genome-wide significant single nucleotide polymorphisms (SNPs) as exposure indicators for MDD (50 SNPs), SCZ (217 SNPs), and BD (16 SNPs). The F-statistics of the above SNPs are all greater than 10, indicating that they are strongly correlated instrumental variables (IVs). The detailed information is specified in the Supplementary Material: Tables 1, 2 and 3.

Data on genetic variants associated with thyroid cancer were obtained by Deutsches the Krebsforschungszentrum (DKFZ) through GWAS [29] and included 1080 European participants, including 649 in the case group and 431 in the control group, as detailed in Table 1. For the bivariate analyses, we used independent genome-wide significant SNPs as indicators of exposure to thyroid cancer (347 SNPs). All 347 SNPs had F-statistics greater than 10, indicating that they were strongly correlated IVs. Specific SNP information is provided in the Supplementary Material: Table 4.

### Selection of genetic instrumental variables

This study was conducted in strict accordance with the quality control steps. First, we selected exposure-related GWAS data and screened SNP loci with genome-wide significance (*p* < 5 × 10^− 8^) for pooled aggregation. Second, to avoid linkage disequilibrium (LD) from affecting the results, we performed a clustering process by setting the parameter (r^2^) threshold (r^2^ < 0.001 and region width = 10,000 kb) to assess LD among SNPs to ensure independence. SNPs need to fulfil three basic assumptions to serve as IVs for exposure factors, and the fulfilment of these assumptions will enhance the testing power and estimation accuracy of IVs: (1) the association assumption: genetic variants are associated with exposure; (2) the independence assumption: genetic variants are independent of confounders between exposure and outcome; and (3) the exclusivity assumption: genetic variants affect the outcome only through exposure [30]. Next, we extracted summary statistics of eligible SNPs from the outcome GWAS; finally, we determined that the SNPs included in the dataset met the instrumental variable requirements. The palindromic sequences were excluded to ensure that the effects of SNPs on exposure and outcome were from the same allele. This series of steps finalized the identification of SNPs that served as genetic IVs for this study.

### Statistical analysis

After coordinating the GWAS effect alleles for MDD, SCZ, BD, and thyroid cancer, we selected three MR approaches. The inverse variance weighted (IVW) test, MR–Egger regression, and weighted median estimator (WME) were used to assess the causal relationship between psychiatric disorders and thyroid cancer risk. The main method of analysis was IVW, while WME and MR–Egger regression were used as complementary methods to IVW estimation, as they provide more reliable estimates under more relaxed conditions [31]. The Cochran’s Q test was used to estimate the heterogeneity of the causal effects of individual gene variants. If horizontal pleiotropy or heterogeneity is detected, fixed-effects IVW analysis should be chosen, and vice versa for random-effects IVW analysis [32, 33]. The IVW method does not take into account the presence of an intercept term and uses the variance of the outcome as the fitting weight. In contrast, the MR–Egger regression method, which is an MR method for assessing the causal effect of genetic variation on the relationship between exposure and outcome, takes into account the presence of an intercept term [34]. This method corrects for polytropic bias and detects directed polytropy but is susceptible to instrumental variable assumptions. When the Egger intercept of a linear regression is close to zero, it indicates the absence of directional pleiotropy, thus satisfying the exclusivity assumption. The weighted median method is a method that combines data from multiple genetic variants into a single causal estimate and requires that more than 50% of the weights come from valid IVs to obtain a reliable estimate of the causal effect [31]. To ensure the reliability of the MR estimates, we also detected outliers that may affect our MR estimates by looking at forest plots, funnel plots, scatter plots, and leave-one-out methods.

To test the first hypothesis of correlation, we also assessed the strength of the relationship between IVs and phenotype using the F-statistic (F = beta^2^/ se^2^, with beta being the allele effect value and SD being the standard deviation), with F > 10 indicating the presence of strongly correlated IVs [35].

All of the above MR-related statistical analyses were implemented using TwoSampleMR in R 4.1.1 software.

## Results

### Selection of genetic instrumental variables

Three sets of genetic instruments were constructed for the forwards MR study after a series of quality control steps. First, we merged the exposure (MDD)- and outcome (thyroid cancer)-related datasets, and after removing 2 palindromic sequences (rs4936276 and rs4730387), we ultimately included 26 SNPs for analysis. The second set of genetic tools was constructed after the same quality control steps, combining the exposure (SCZ) and outcome (thyroid cancer) datasets and deleting six palindromic sequences (rs12363019, rs217310, rs2470951, rs2944821, rs7709645, rs9925915) before finally including 111 SNPs that were analysed. A third set of genetic instruments was constructed following the same quality control steps, combining exposure (BD) and outcome (thyroid cancer), and after deleting 2 palindromes sequences (rs10455979, rs5758065), and finally included 9 SNPs for analysis. The F-statistics of the above SNPs were greater than 10, indicating that they were strongly correlated with each other (Supplementary Material: Tables 1, 2 and 3).

Three sets of genetic instruments were constructed in the reverse MR study after a series of quality control steps. First, we combined exposure (thyroid cancer) and outcome (MDD)-related datasets, resulting in the inclusion of 331 SNPs for analysis. The second set of genetic instruments was constructed following the same quality control steps, combining the exposure (thyroid cancer) and outcome (SCZ) datasets, resulting in the inclusion of 338 SNPs for analysis. A third set of genetic instruments was constructed following the same quality control steps, combining the exposure (thyroid cancer) and outcome (BD) data and ultimately including 338 SNPs for analysis. The F values of the above IVs were all > 10, indicating reliable results without weak bias.

### Mendelian randomization analysis

In our study, we explored the causal relationship between psychiatric disorders (MDD, SCZ, and BD) and thyroid cancer using psychiatric disorders as exposure factors. The results of the IVW analysis showed a significant association between MDD and the risk of thyroid cancer (OR = 3.956 95% CI = 1.177–13.299; *P* = 0.026), confirming the possibility of an increased risk of thyroid cancer due to MDD. These findings were reinforced by the results obtained by the WME method (OR = 5.563 95% CI = 0.998–31.008; *P* = 0.050), which were consistent with those of the IVW method. However, the results of the MR–Egger regression (OR = 76.975 95% CI = 0.008-766576.333; *P* = 0.364) showed that the difference in the effect of MDD and thyroid cancer was not statistically significant (Table 2), which may be due to the high false-positive rate of false-negative results from this method. Nevertheless, the IVW and WME methods suggest that MDD may increase the risk of thyroid cancer.

**Table 2:** Estimates of the causal effect of different psychiatric disorders on thyroid cancer incidence

Exposure	Outcome	IVW (fixed-effect)				MR–Egger						Weighted median	
		OR (95% CI)	P value	Cochran Q	P value	OR (95% CI)	P value	Cochran Q	P value	MR–Egger intercept	P value	OR (95% CI)	P value
Major depressive disease	Thyroid cancer	3.956 (1.177–13.299)	0.026	22.594	0.601	76.975 (0.008-766576.333)	0.364	22.187	0.568	-0.087	0.530	5.563 (0.998–31.008)	0.050
Schizophrenia	Thyroid cancer	1.532 (1.123–2.088)	0.007	112.947	0.404	1.867 (0.438–7.964)	0.401	112.870	0.381	-0.012	0.785	1.599 (1.014–2.521)	0.043
Bipolar disorder	Thyroid cancer	0.919 (0.460–1.836)	0.811	6.246	0.620	155.530 (1.613-14998.511)	0.067	1.287	0.989	-0.477	0.061	1.003 (0.393–2.559)	0.996

In addition, we found that genetic susceptibility to SCZ was correlated with thyroid cancer (OR = 1.532 95% CI = 1.123–2.088; *P* = 0.007). The results of the WME method analysis based on the median estimate (OR = 1.599 95% CI = 1.014–2.521; *P* = 0.043) also support that SCZ may increase the risk of thyroid cancer (Table 2).

However, no causal relationship between BD and thyroid cancer was found in any of the MR analyses. In addition, we performed reverse MR analysis, which showed no evidence of a causal relationship between genetic susceptibility to thyroid cancer and psychiatric disorders (MDD, SCZ, and BD), ruling out the possibility of reverse causation (Supplementary Material: Table 5).

### Sensitivity analysis

For sensitivity analysis, we first tested for heterogeneity of results using Cochran’s Q for IVW and MR–Egger regression. The results showed that the p values of the analyses were greater than 0.05, which indicated that there was no significant heterogeneity in our study. Similarly, the MR–Egger intercept method results also showed no horizontal pleiotropy (all p values greater than 0.05). We also constructed funnel plots and leave-one-out plots. The funnel plot was roughly symmetrical, indicating a relatively low risk of bias and high reliability of the results. A leave-one-out plot was generated to reject SNPs one by one, and the analysis showed that the causal relationship between psychiatric disorders and thyroid cancer was largely not driven by a single SNP. We also examined scatter plots, in which each point represents an instrumental variable. Each horizontal solid line in the forest plot reflects a single SNP estimated using the Wald ratio method. Leave-one-out, scatter, funnel, and forest plots can be found in the supplementary materials.

In the inverse sensitivity analyses, Cochran’s Q test revealed heterogeneity between the effects of thyroid cancer on MDD, SCZ, and BD. Therefore, IVW analysis under a random effects model was chosen to balance the heterogeneity of the results. However, it is noteworthy that no heterogeneity was found in thyroid cancer patients with MDD or SCZ. p values for the MR–Egger intercept method were all greater than 0.05, suggesting that there was no horizontal pleiotropy in the results. Leave-one-out, scatter, funnel, and forest plots can be found in the supplementary materials.

## Discussion

With the increasing prevalence of psychiatric disorders and thyroid cancer, there is an increasing overlap between them, prompting us to delve deeper into their relationship. This study is the first two-sample bidirectional MR study of psychiatric disorders (MDD, SCZ, BD) and thyroid cancer. Our MR study showed a significant causal association between MDD and SCZ and thyroid cancer, whereas no such association was found between BD and thyroid cancer. Reverse MR analysis ruled out the possibility of reverse causation.

MR studies have the advantage of effectively avoiding confounding bias. Because SNPs are randomly assigned at conception, MR is also able to exclude reverse causality effects relative to observational studies, thus enhancing the credibility of causal inferences. We suggest the following possible mechanisms for the positive causal relationship between MDD and thyroid cancer: First, MDD may lead to abnormal functioning of the hypothalamic–pituitary–thyroid (HPT) axis, which in turn affects thyroid hormone levels and thyroid-stimulating hormone (TSH) secretion. TSH is a key factor in promoting thyroid cell proliferation, and abnormal TSH levels may increase the risk of thyroid nodules and cancer. Patients with early-stage MDD may suffer from thyroid and metabolic dysfunction [36]. Data from the study showed that 26.2% of depressed patients had abnormal thyroid function, 18.3% of whom had MDD, and 62.4% of the study population was female [37]. The results of a multicentre study by the European Antidepressant Study Group showed that the prevalence of hypothyroidism and hyperthyroidism in patients with MDD was 13.2% and 1.6%, respectively [38]. These results imply that MDD may regulate thyroid hormone levels through the HPT axis, thereby affecting thyroid function and structure. Abnormal HPT axis function has been the focus of research on neuroendocrine mechanisms in patients with psychiatric disorders, and our findings provide insight into the relationship between genetic susceptibility to MDD and thyroid cancer. Second, MDD leads to elevated peripheral inflammatory marker levels [39, 40], which induce chronic inflammation and gene mutations in the thyroid gland. These peripheral inflammatory markers include interleukins (ILs), tumour necrosis factor (TNF), and C-reactive protein (CRP), which can affect thyroid tissues through blood circulation or neuroendocrine pathways. Chronic inflammation can mediate tumour development, and the two are interconnected through endogenous and exogenous pathways. Chronic inflammation of the thyroid gland may contribute to genetic defects through the secretion of high levels of mutagenic agents (e.g., reactive oxygen species and nitric oxide) [41]. Finally, MDD may be associated with type C personality, which is characterized by abnormal emotional expression and abnormal emotion regulation that may affect the immune system and endocrine function [42–44]. Some scholars [43] regard negative emotions as an independent risk factor for the occurrence of thyroid cancer and believe that the persistence or recurrence of depression and anxiety is a stress factor for the human body and that stress causes changes in the cerebral cortex and hypothalamus, which can directly or indirectly suppress the immune system and interfere with the endocrine function of the body [44], thus affecting the normal synthesis and release of thyroid hormones and triggering thyroid nodules and increasing the likelihood of thyroid cancer.

There may be different aetiologies regarding the genetic susceptibility of patients with SCZ to an increased risk of thyroid cancer. Beginning in the late 19th century, when hypothyroidism was connected with psychiatric disorders, an increasing number of clinical studies have shown a strong independent association between SCZ and hypothyroidism [45]. A recent community-based cross-sectional study comparing patients with SCZ (*n* = 1252) and healthy controls matched for age, sex, socioeconomic status and ancestry (*n* = 3756) revealed that the incidence of hypothyroidism in patients with SCZ increased after treatment but not before diagnosis [22]. Similarly, in observational studies, patients with SCZ are more likely to have abnormal thyroid function after initiating treatment with antipsychotics [46, 47]. Thus, the use of antipsychotics may lead to abnormalities in thyroid function, although it is not clear whether the HPT axis can be directly affected. A systematic review and meta-analysis summarizing 19 studies suggested that TSH levels may be reduced at the onset of psychosis and elevated in patients with multiple episodes of psychosis [48]. Studies have shown that dopamine or dopamine agonists inhibit TSH secretion, and a possible explanation for the elevated TSH levels lies in the fact that antidopaminergic drugs used to treat SCZ inhibit dopamine neurotransmission, which may cause elevated TSH levels [49]. Thus, there may be a causal relationship between hypothyroidism or elevated TSH levels and the manifestations of SCZ. Thyroid hormones not only play a role in the dopaminergic system but also in the regulation of serotonergic, glutamatergic, and GABAergic networks [50]. During neurodevelopment, thyroid hormones play a critical role, and their deficiency may severely impair the development of neural tissues, leading to abnormalities and damage in the cerebellar cortex and cerebral cortex [51]. In the adult brain, thyroid hormone interacts with glial cells to regulate immune responses and neurotransmitter release and to control neuronal metabolism.

However, our study did not find conclusive evidence to support a causal role between genetic susceptibility to BD and thyroid cancer risk. To date, the association between affective disorders and thyroid cancer has not been widely reported. Although previous epidemiologic studies using case–control methods have suggested an association between BD and abnormal thyroid function [52–54], this topic has yet to be thoroughly investigated. One large meta-analysis reported that thyroid hormones may affect neurodevelopment by modulating the brain’s serotonin system [43]. The current preferred mood stabilizer for maintenance treatment of BD is lithium, although lithium alters thyroid functional status [55]. However, little is known about the pathophysiologic role of thyroid hormones in BD, and genetically, our study did not find a direct relationship between BD and thyroid cancer. However, further validation with larger datasets is needed in the future.

Our study has important implications for understanding the potential link between psychiatric disorders and thyroid cancer, as well as providing new ideas and strategies for the prevention and treatment of these common psychiatric disorders. For example, we can reduce the risk of thyroid cancer by screening and treating psychiatric disorders or improve the clinical management of psychiatric disorders by monitoring and regulating thyroid hormone levels. Specifically, we could conduct thyroid function testing and interventions in patients with psychiatric disorders, along with psychological assessment and treatment in patients with thyroid cancer. This integrated approach is expected to mitigate, to some extent, the adverse effects of psychiatric disorders and thyroid cancer on patients’ quality of life and socioeconomic status. In addition, our study provides clues and research directions for in-depth exploration of the potential relationships between MDD and thyroid cancer and between SCZ and thyroid cancer. More experimental and clinical studies are needed in the future to validate our findings and reveal the molecular cellular mechanisms underlying the causal relationship between psychiatric disorders and thyroid cancer.

In conclusion, our study is the first two-sample bidirectional MR study on the causal relationship between psychiatric disorders and thyroid cancer. Although our study provides useful insights for obtaining a deeper understanding of the relationship between psychiatric disorders and thyroid cancer, there are several limitations to consider. First, we used European population-based GWAS data to select IVs and obtain exposure data, which may limit the generalizability and applicability of our results. Second, our IVs were based on the use of a single nucleotide polymorphism-based design, which may not fully capture genetic variability in exposure or outcome. Finally, due to the limitations of the dataset, the number of thyroid cancer patients in the study was relatively small, which may have led to bias.

## Conclusions

In summary, our study provides some suggestive evidence that MDD and SCZ are positively associated with thyroid cancer. This finding may have implications for health care policies regarding psychiatric disorders and thyroid cancer. Considering the high prevalence of psychiatric disorders and thyroid cancer in the general population, revealing the causal relationship between psychiatric disorders and thyroid cancer is important for public health policies for early prevention and timely prevention.

### Electronic supplementary material

Below is the link to the electronic supplementary material.

Supplementary Material 1

## Data Availability

Major depression：https://gwas.mrcieu.ac.uk/datasets/ieu-b-102/;Schizophrenia;https://gwas.mrcieu.ac.uk/datasets/ieu-b-5099/; Bipolar disorder:https://gwas.mrcieu.ac.uk/datasets/ieu-b-41/; Thyroid caencer:https://gwas.mrcieu.ac.uk/datasets/ieu-a-1082/;

## Abbreviations

MDD: Major depressive disease
SCZ: schizophrenia
BD: bipolar disorder
MR: Mendelian randomization
IVs: instrumental variables
IVW: inverse variance weighted
US-FNA: ultrasound-guided Fine Needle Aspiration
GWAS: genome-wide association study
PGC: Psychiatric Genomics Consortium
SNPs: single nucleotide polymorphisms
DKFZ: Deutsches the Krebsforschungszentrum
LD: linkage disequilibrium
WME: Weighted Median Estimator
HPT: hypothalamic–pituitary–thyroid
TSH: thyroid-stimulating hormone
IL: interleukins
TNF: tumour necrosis factor
CRP: C-reactive protein
