# Supplementary material for: Association of major depression, schizophrenia and bipolar disorder with thyroid cancer: a bidirectional two-sample mendelian randomized study
Source: BMC Psychiatry. 2024 Apr 9;24:261. doi: 10.1186/s12888-024-05682-7 (PMC11003083; doi:10.1186/s12888-024-05682-7)
Supplement: Supplementary file 1 — Supplementary Material 1 [file 12888_2024_5682_MOESM1_ESM.docx]

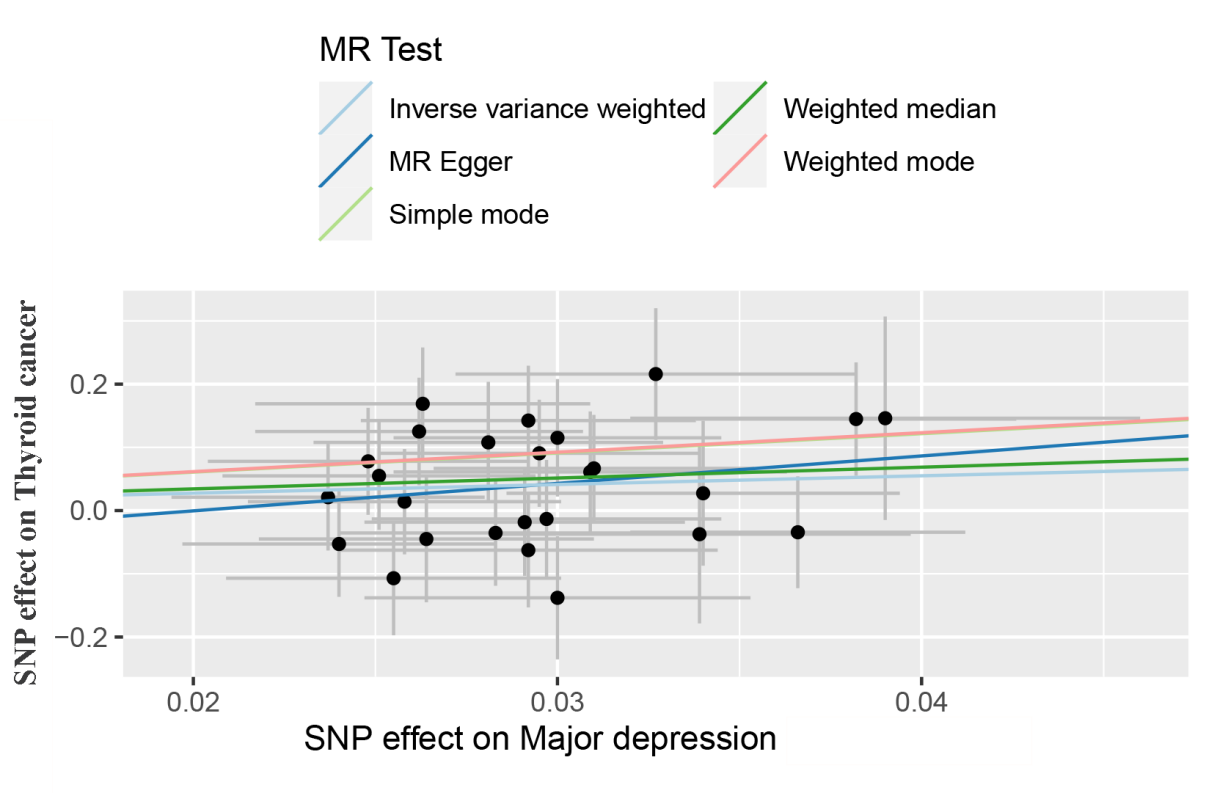


Figure S1: Scatter plot of the causal effect of major depression on thyroid cancer, with the slope of each line corresponding to estimated causal effect per method.


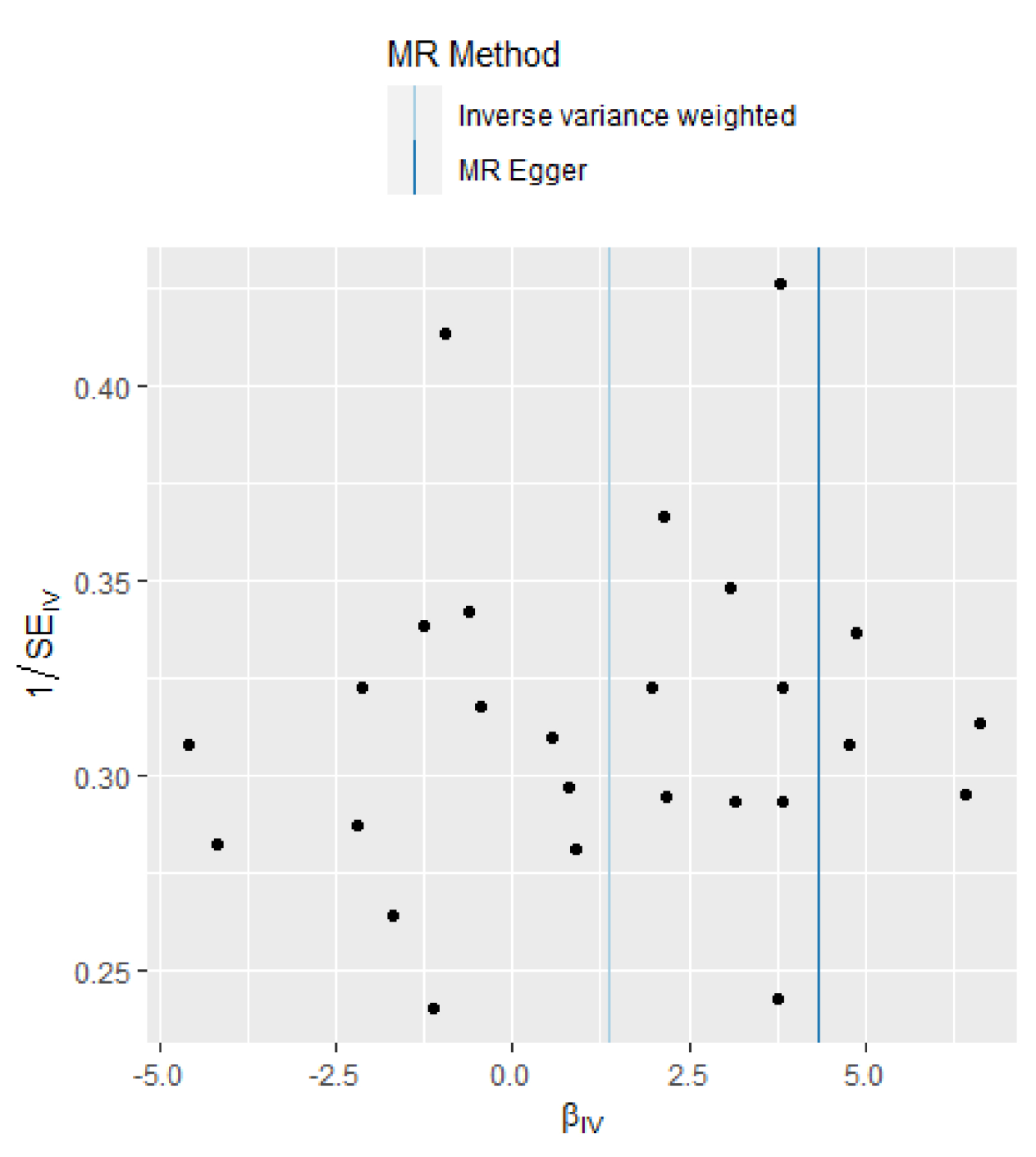


Figure S2: Funnel plot of the causal effect of major depression on thyroid cancer.


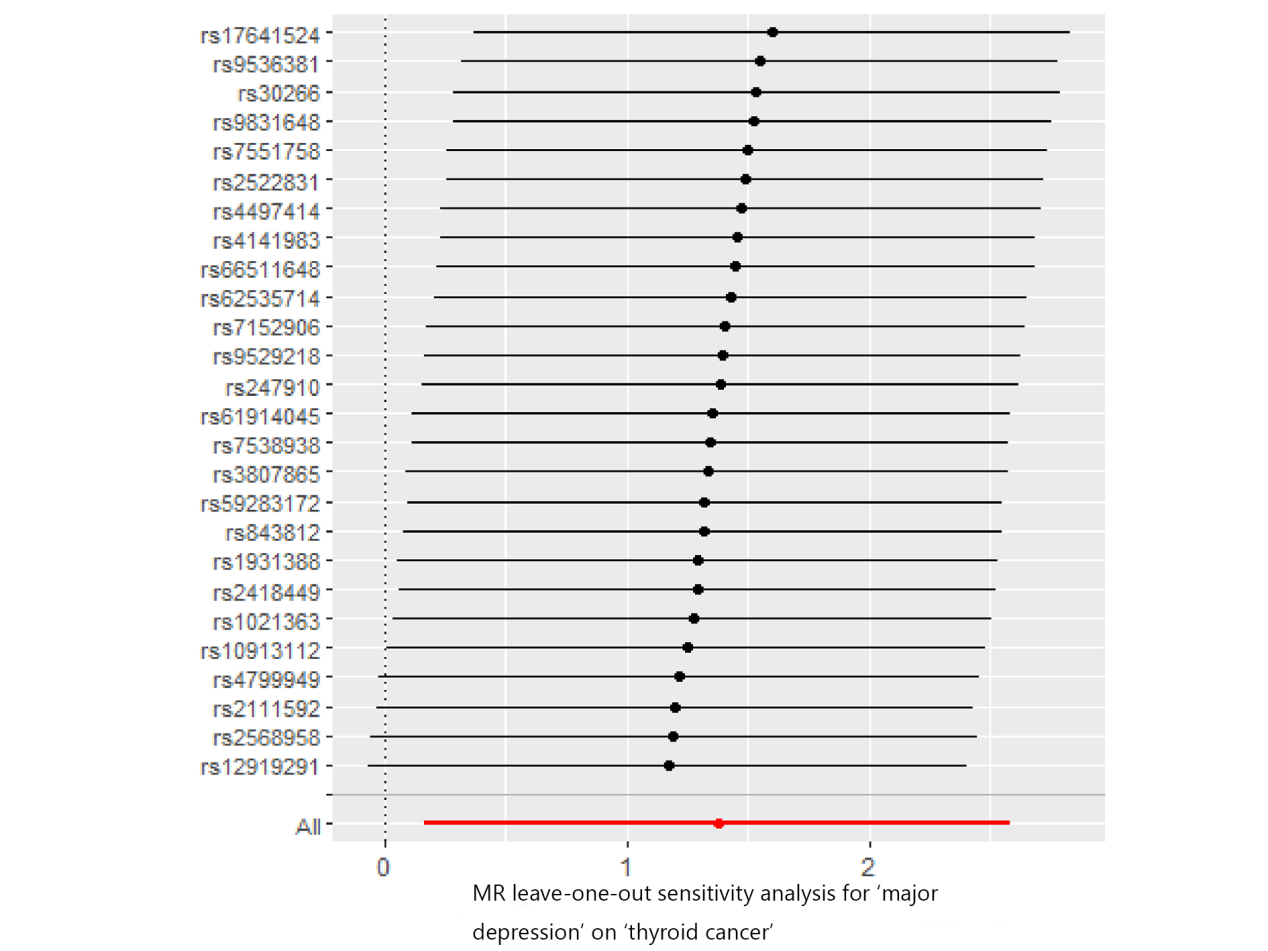


Figure S3: The leave-one-out method for the causal effect of major depression on thyroid cancer.


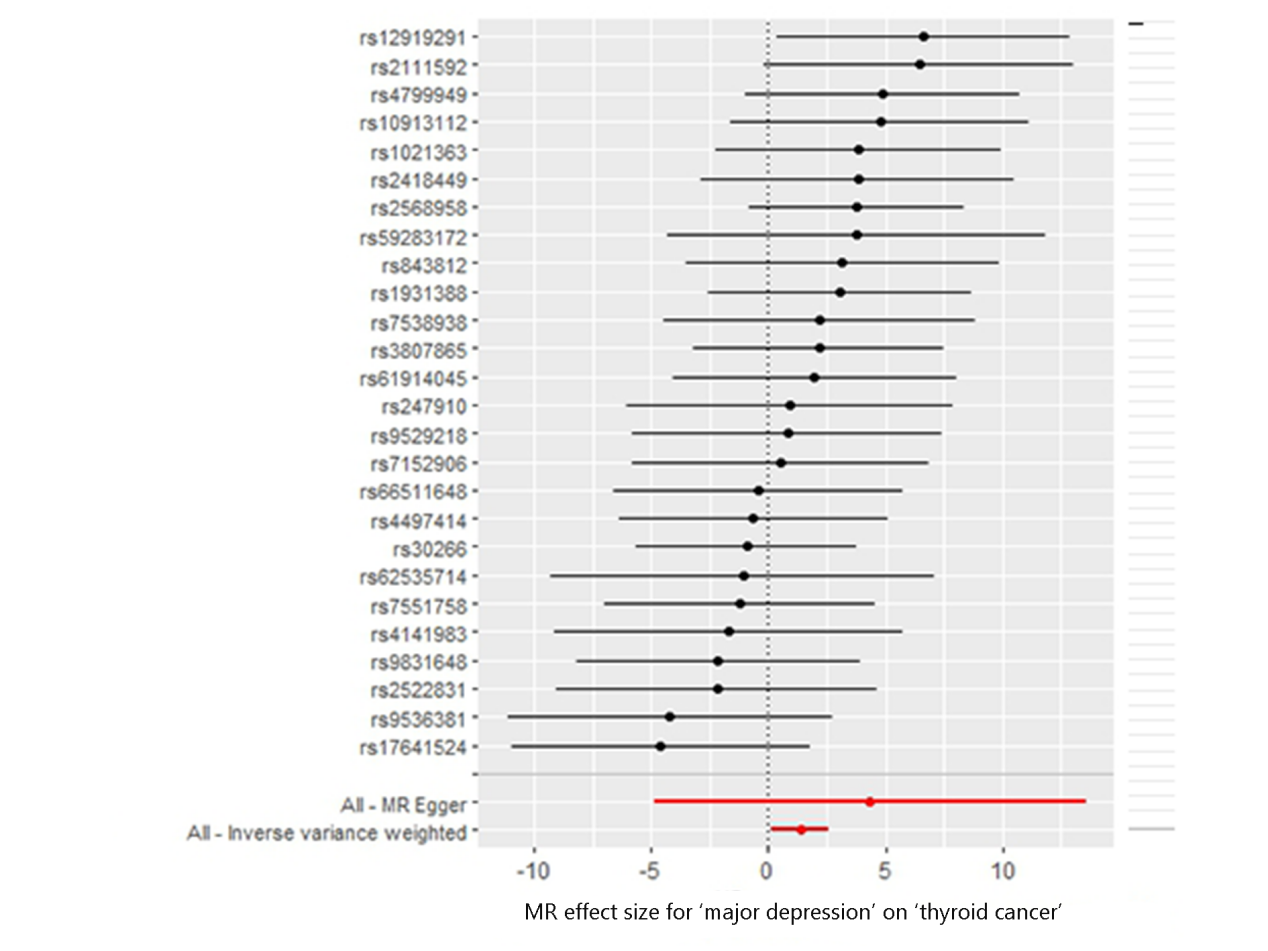


Figure S4: Forest plots of the causal effect of major depression on thyroid cancer.


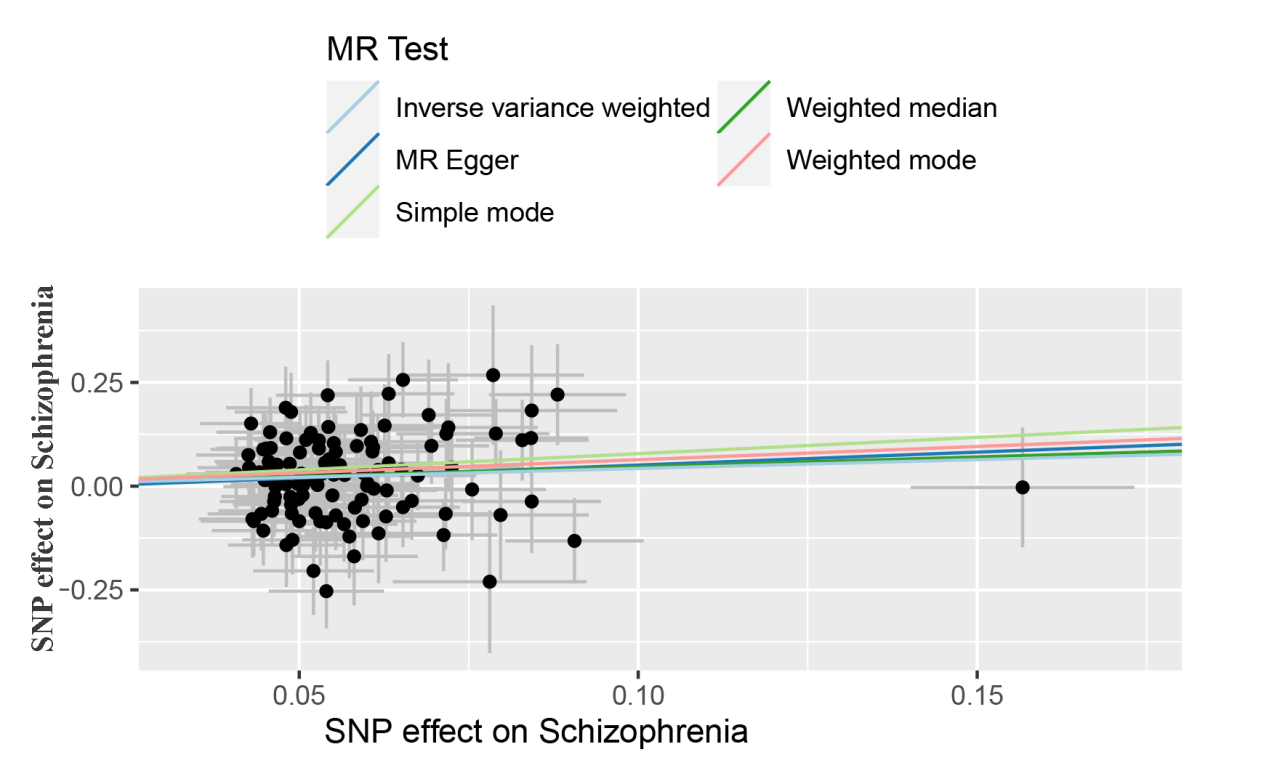


Figure S5: Scatter plot of the causal effect of schizophrenia on thyroid cancer, with the slope of each line corresponding to estimated causal effect per method.


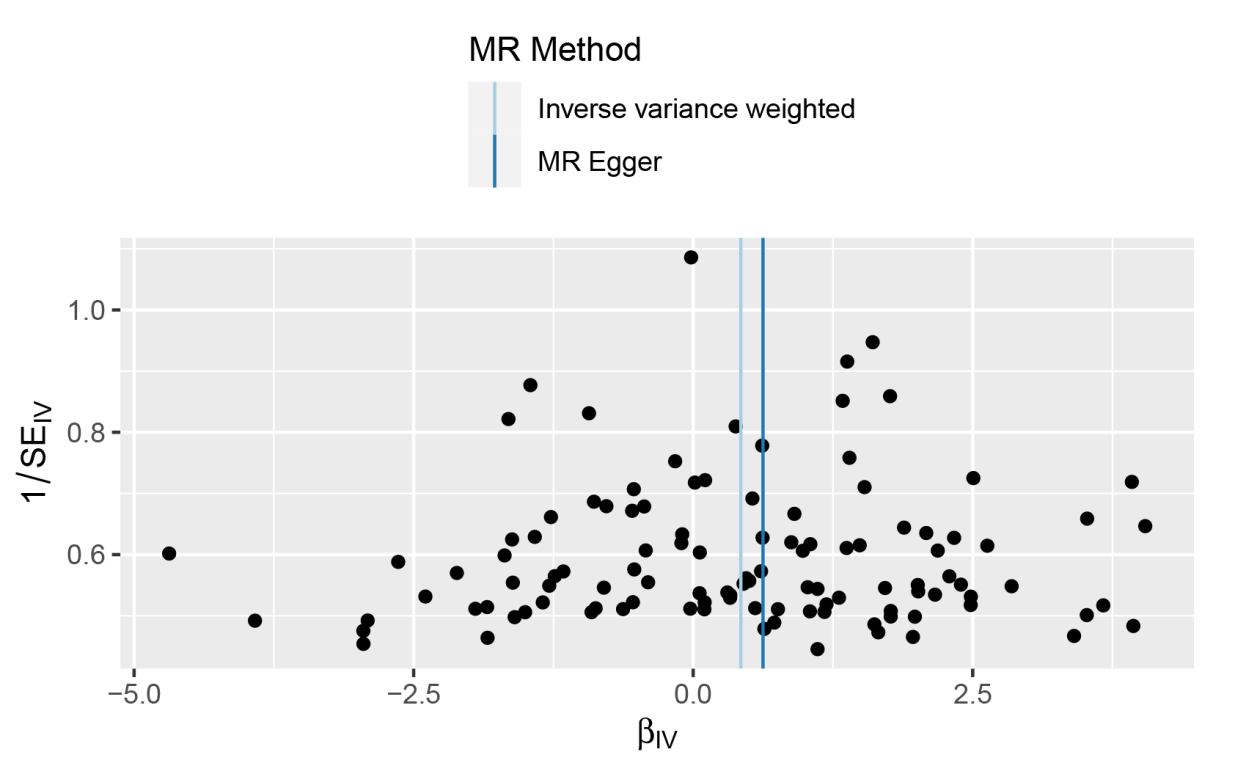


Figure S6: Funnel plot of the causal effect of schizophrenia on thyroid cancer.


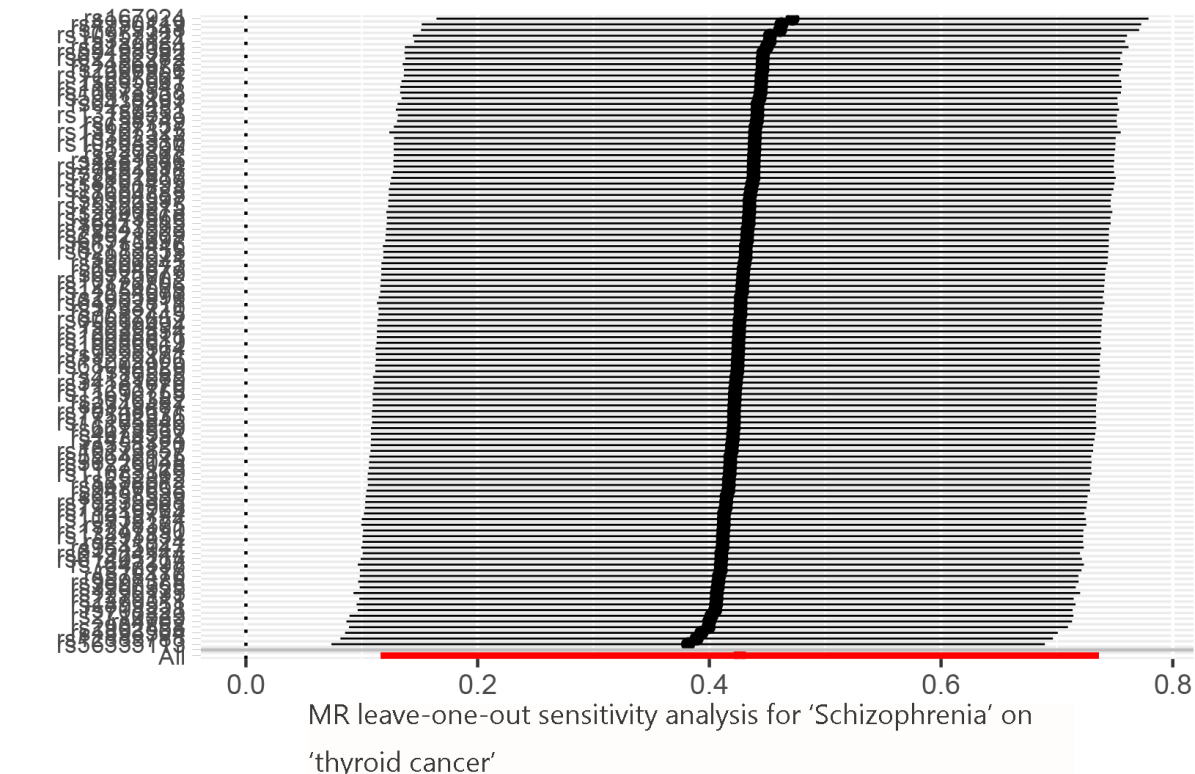


Figure S7: The leave-one-out method for the causal effect of schizophrenia on thyroid cancer.


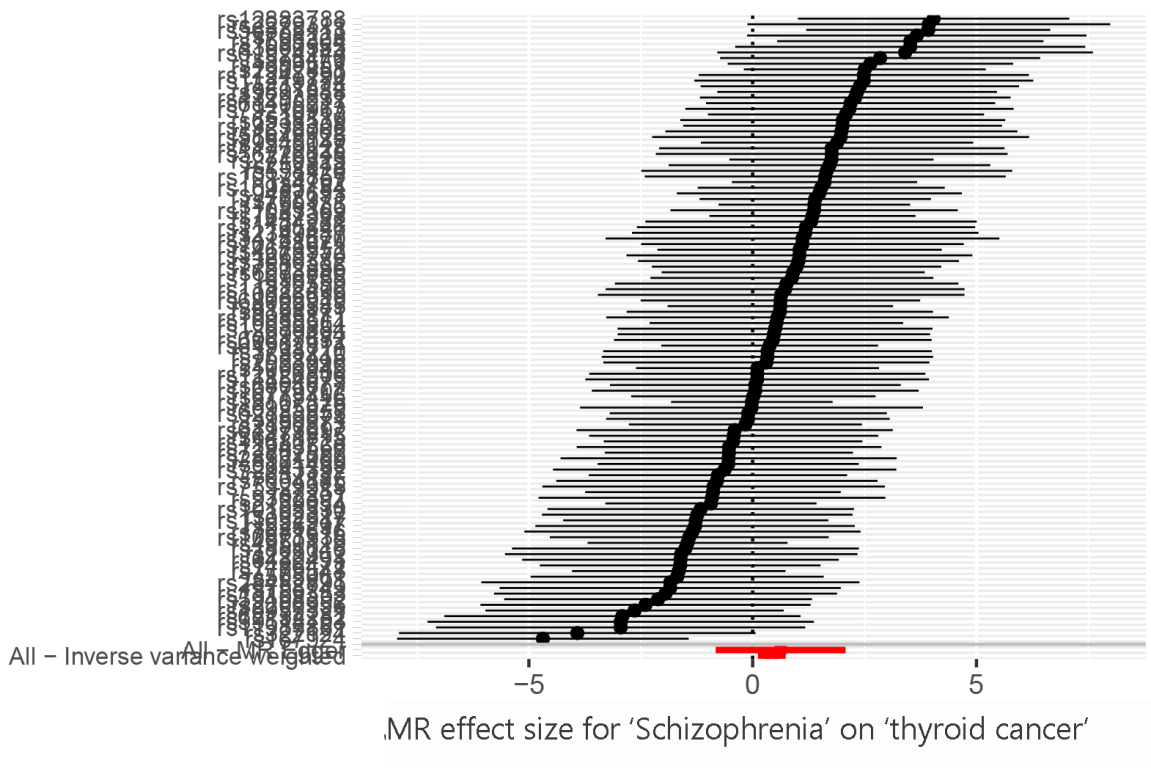


Figure S8: Forest plots of the causal effect of schizophrenia on thyroid cancer.


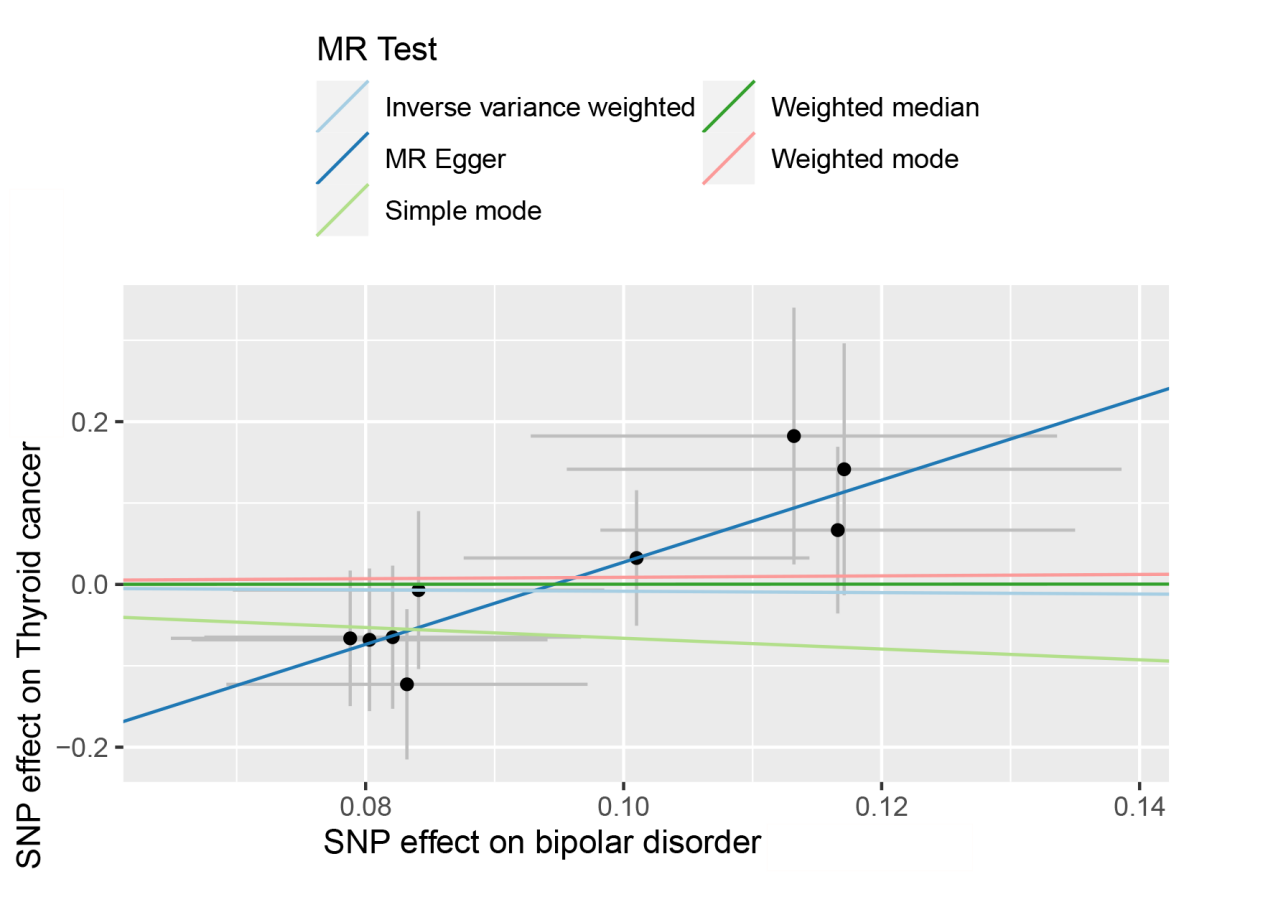


Figure S9: Scatter plot of the causal effect of bipolar disorder on thyroid cancer, with the slope of each line corresponding to estimated causal effect per method.


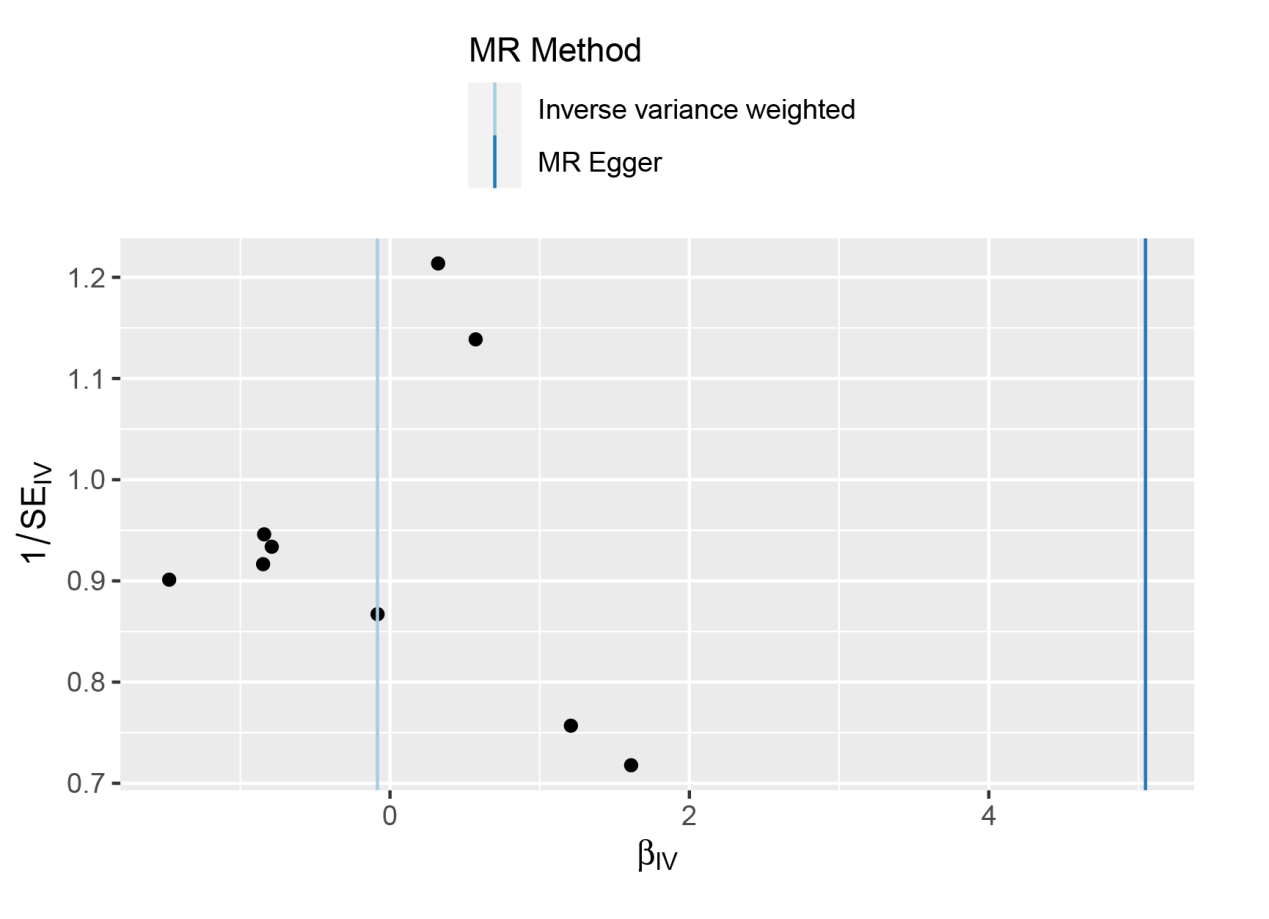


Figure S10: Funnel plot of the causal effect of bipolar disorder on thyroid cancer.


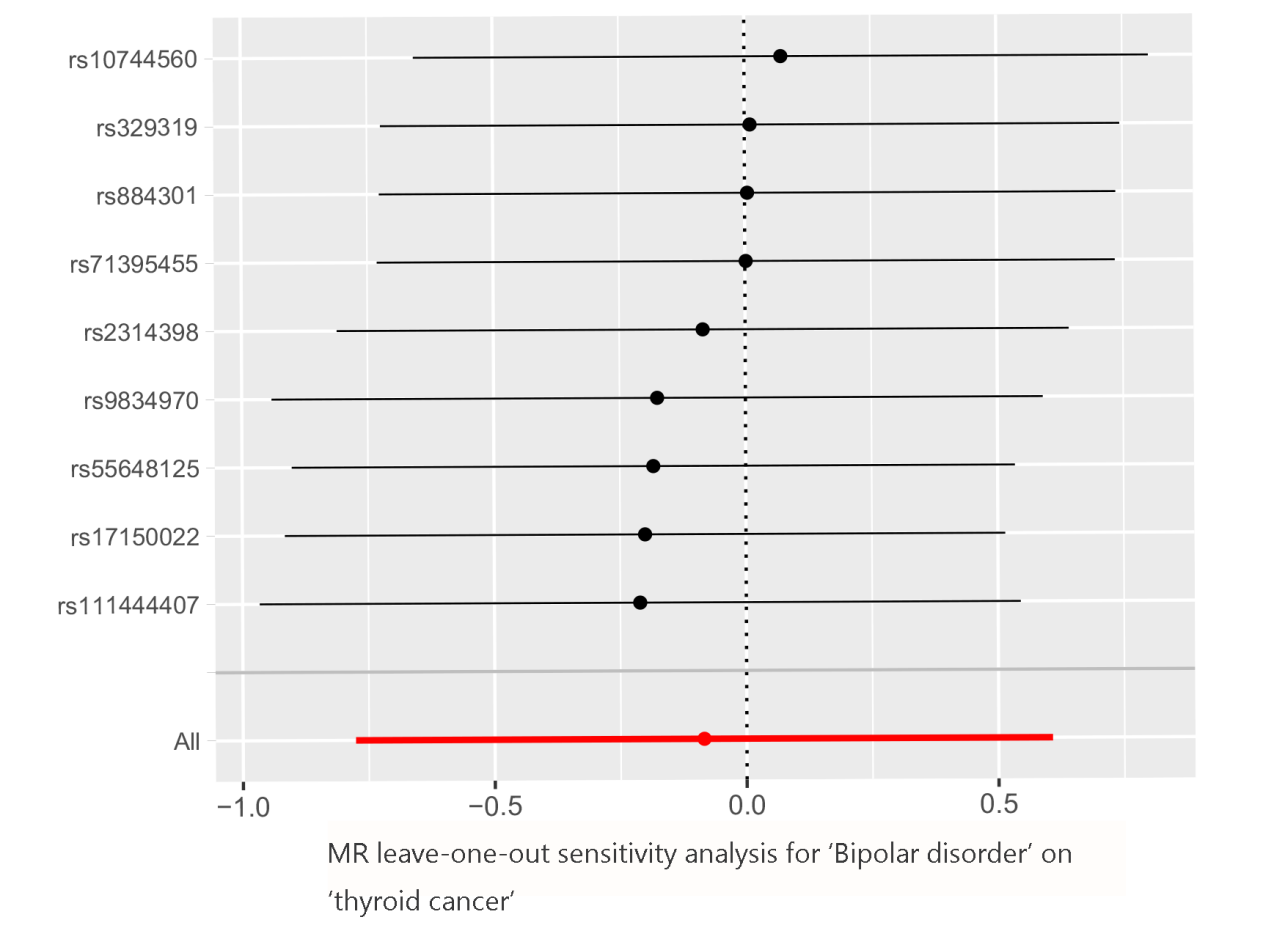


Figure S11: The leave-one-out method for the causal effect of bipolar disorder on thyroid cancer.


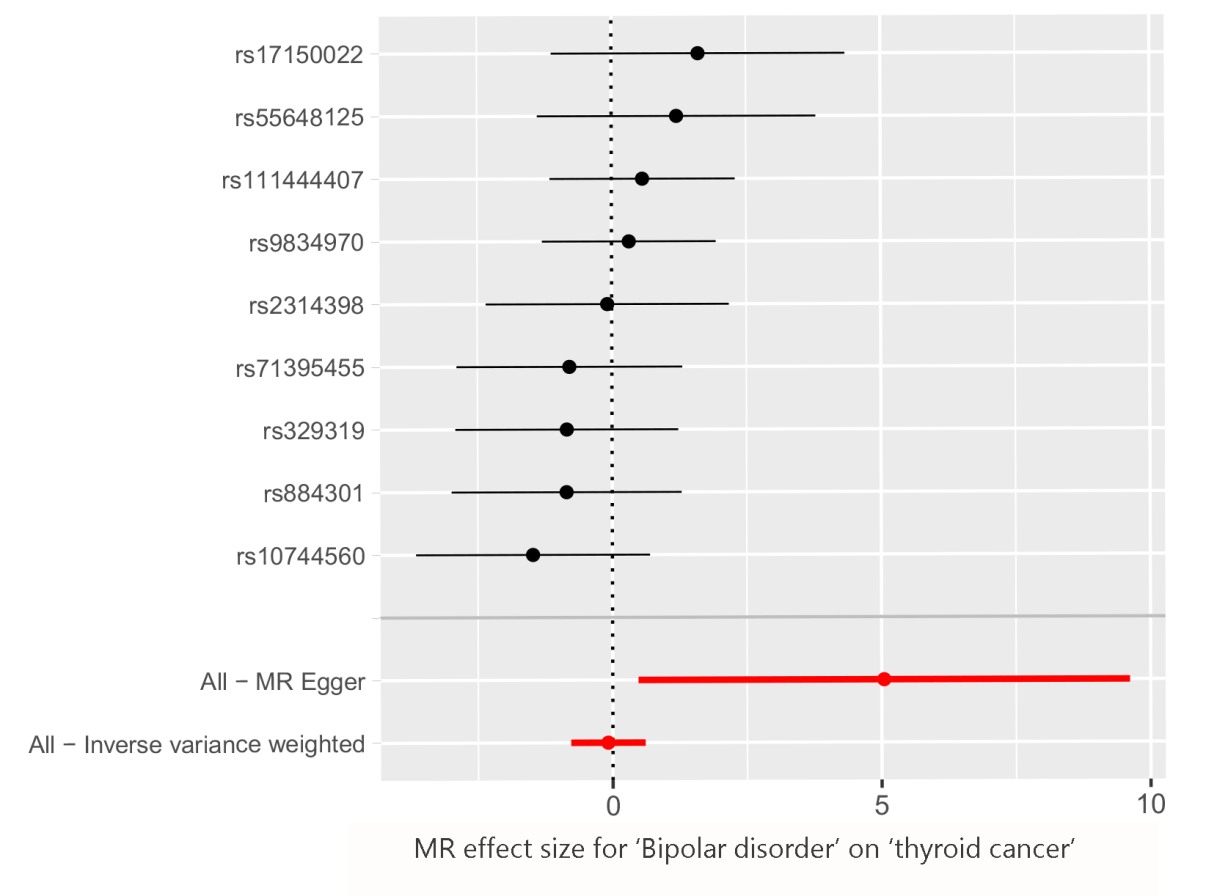


Figure S12: Forest plots of the causal effect of bipolar disorder on thyroid cancer.


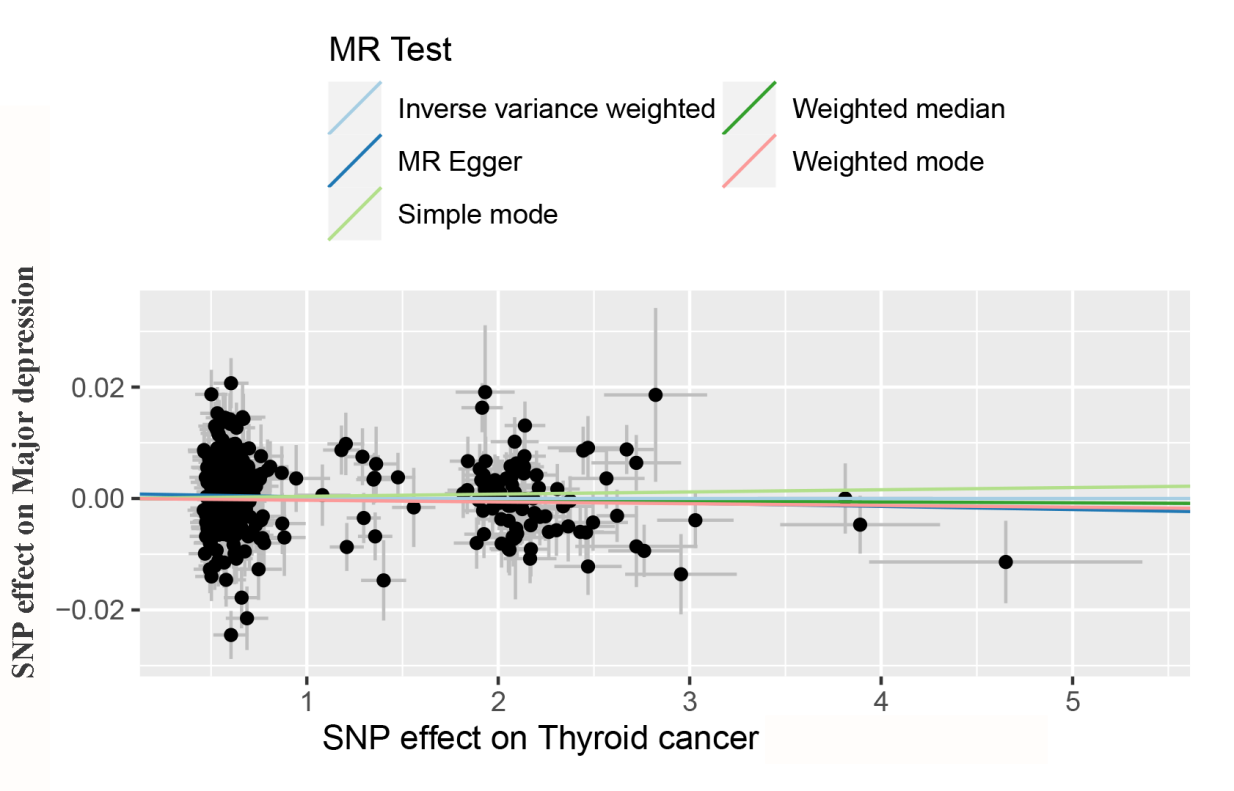


Figure S13: Scatter plot of the causal effect of thyroid cancer on major depression, with the slope of each line corresponding to estimated causal effect per method.


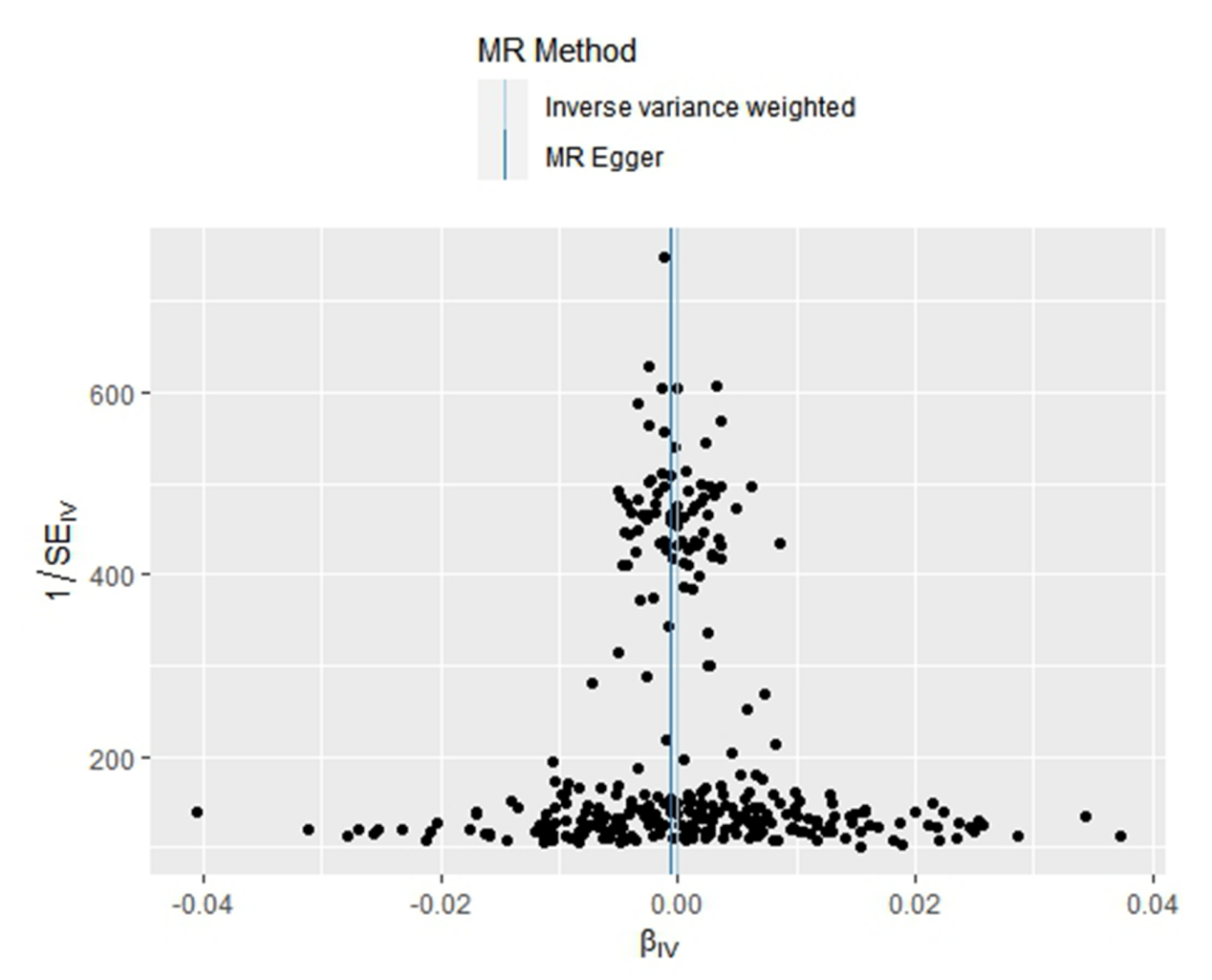


Figure S14: Funnel plot of the causal effect of thyroid cancer on major depression.


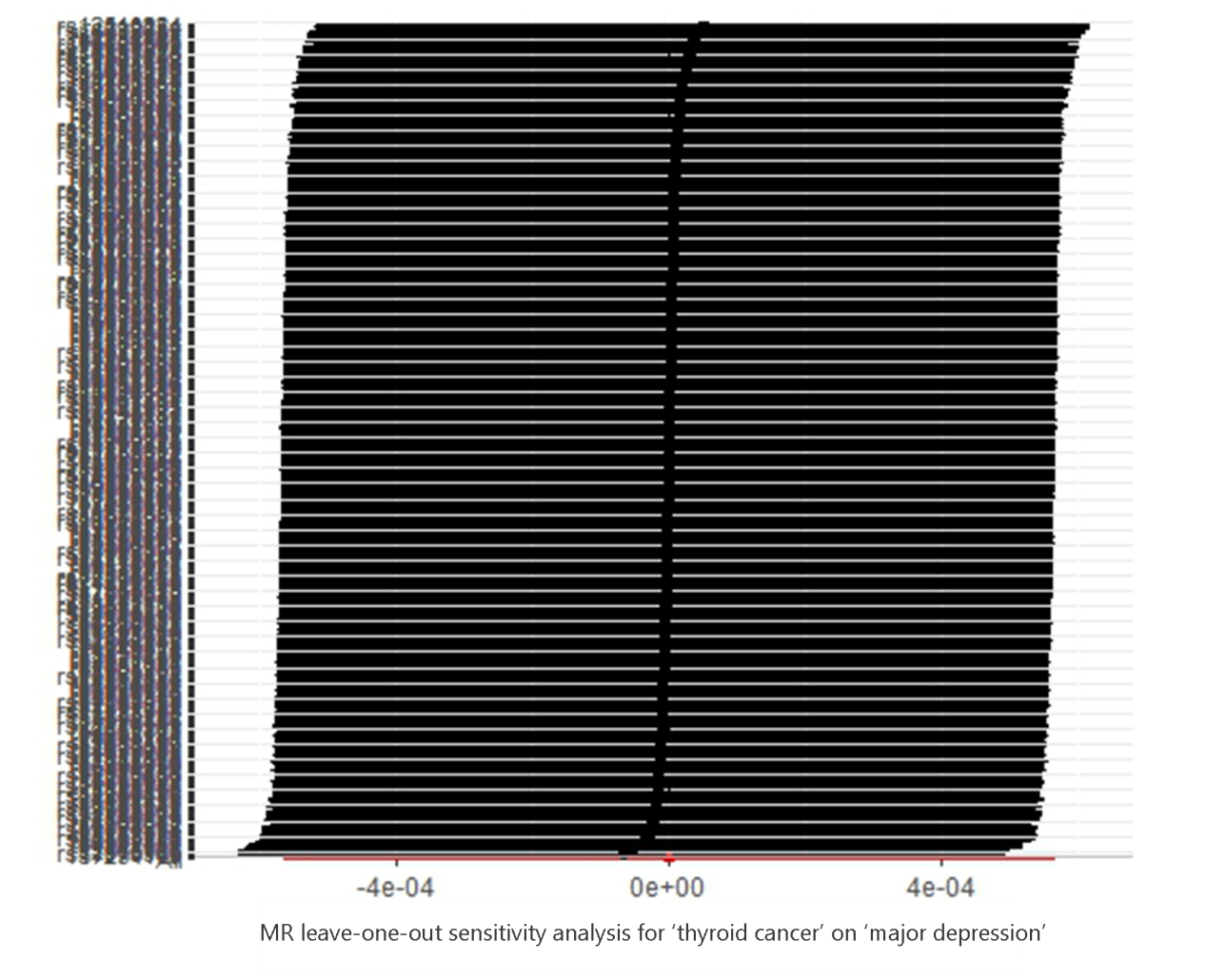


Figure S15: The leave-one-out method for the causal effect of thyroid cancer on major depression.


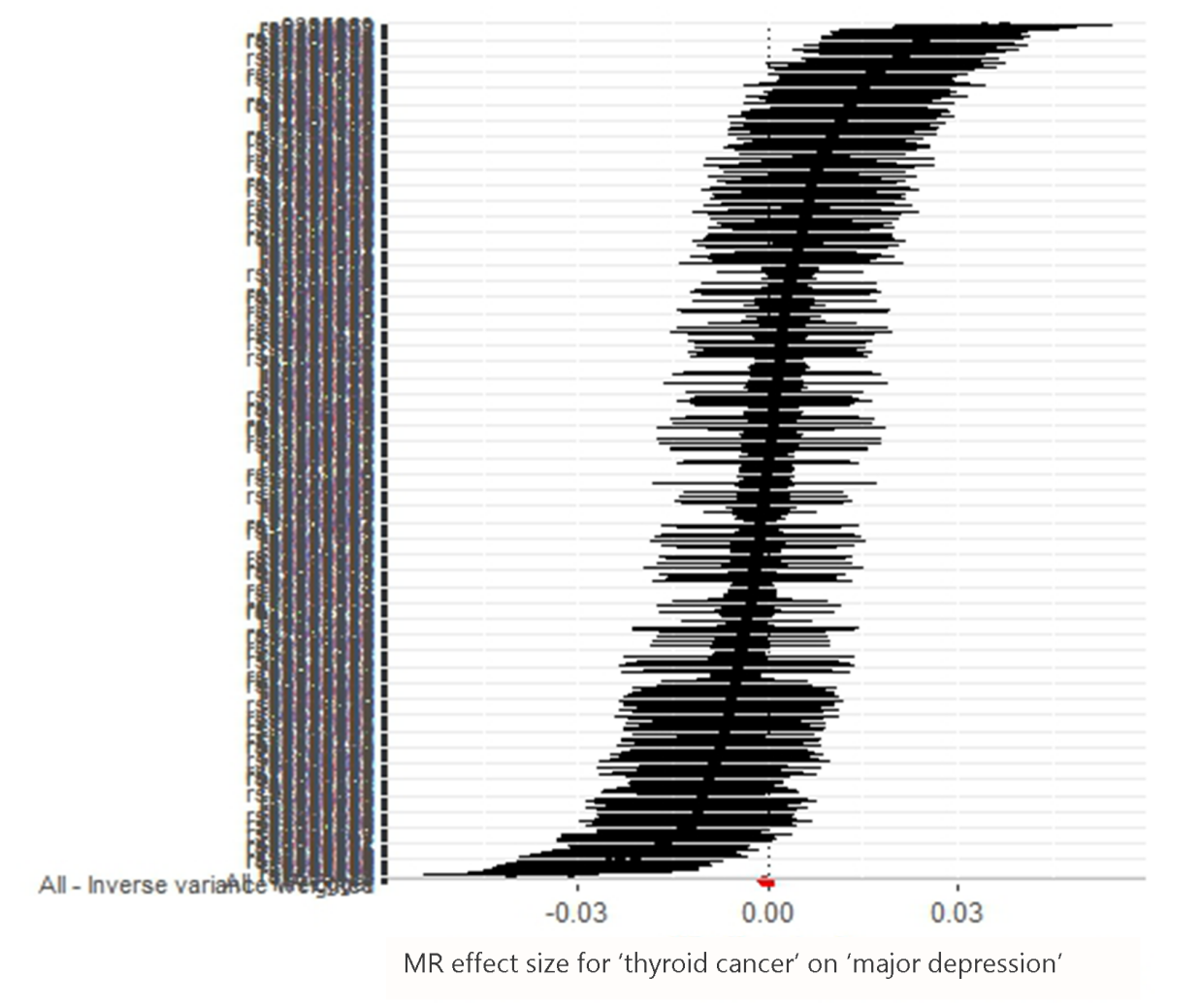


Figure S16: Forest plots of the causal effect of thyroid cancer on major depression.


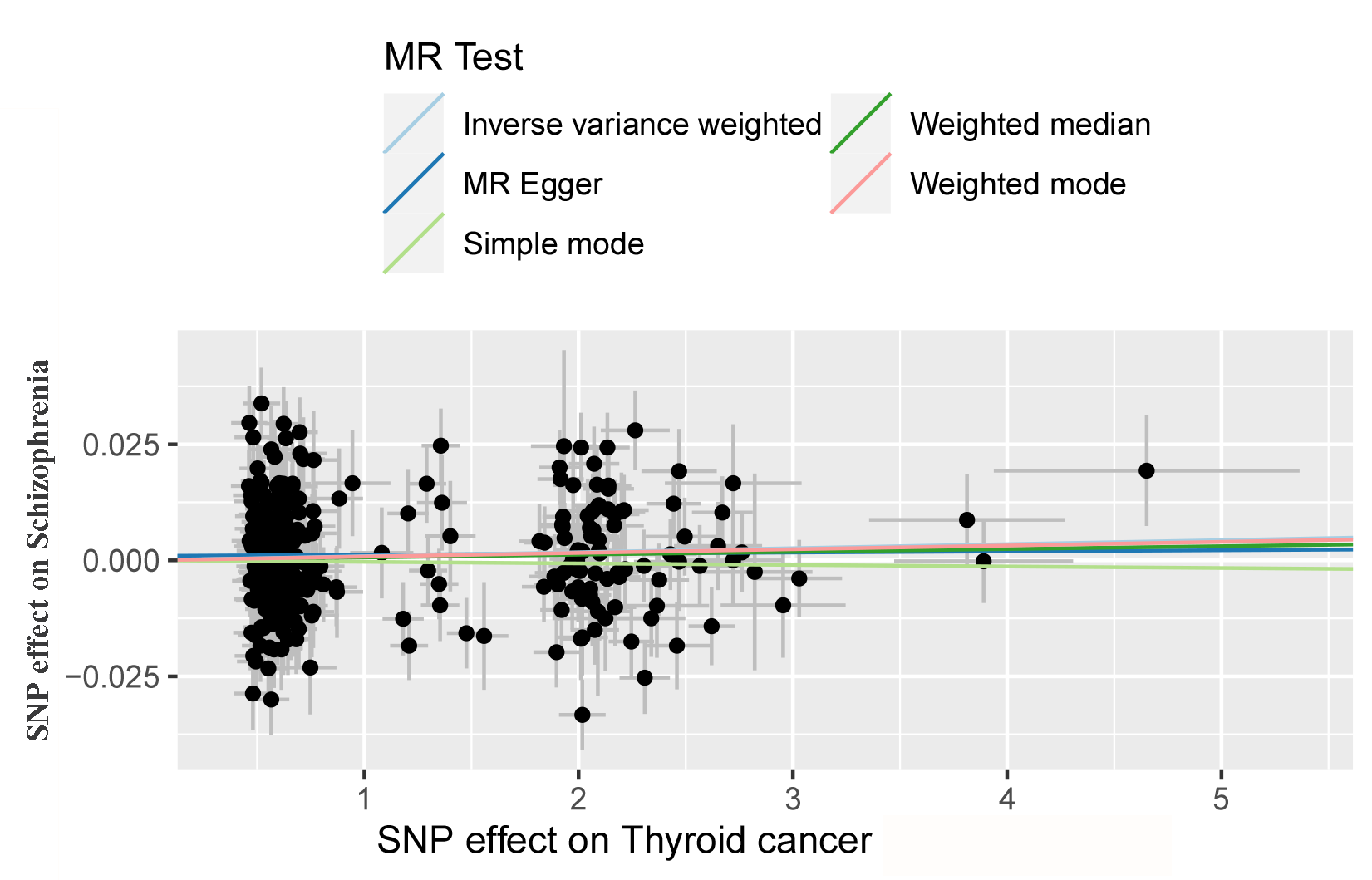


Figure S17: Scatter plot of the causal effect of thyroid cancer on schizophrenia, with the slope of each line corresponding to estimated causal effect per method.


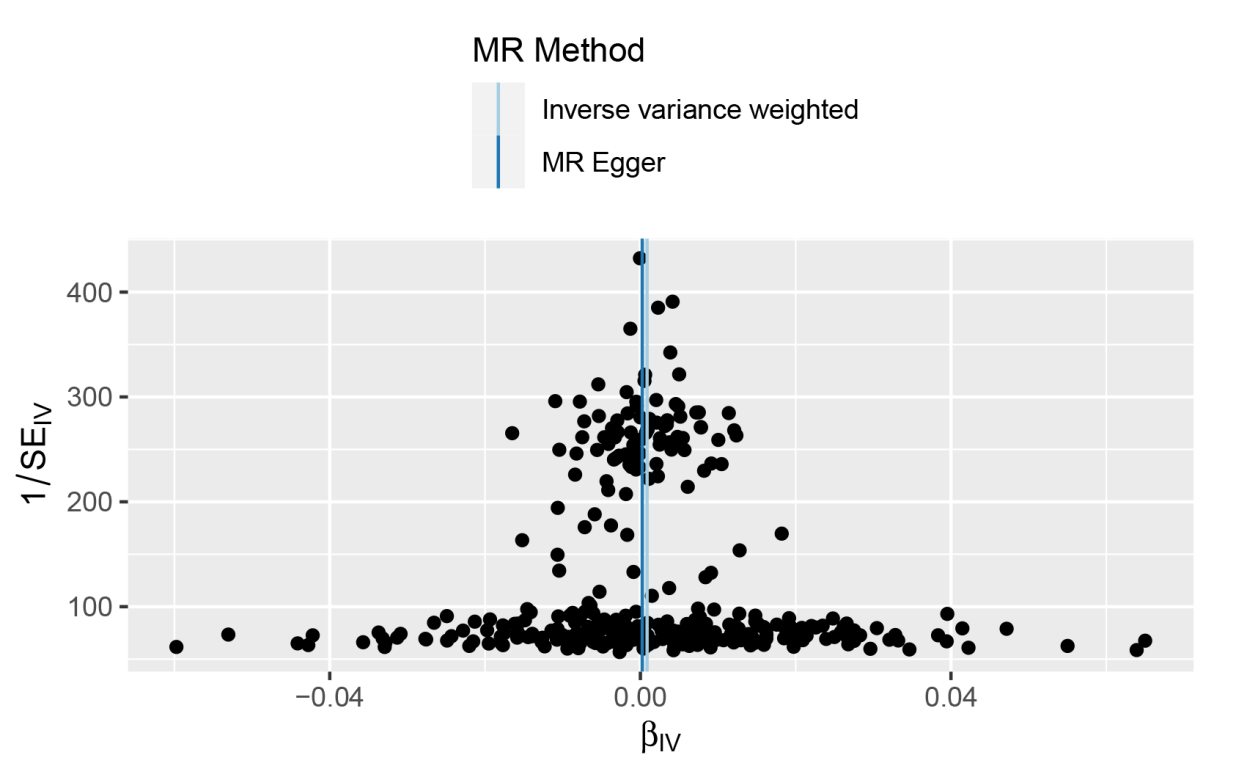


Figure S18: Funnel plot of the causal effect of thyroid cancer on schizophrenia.


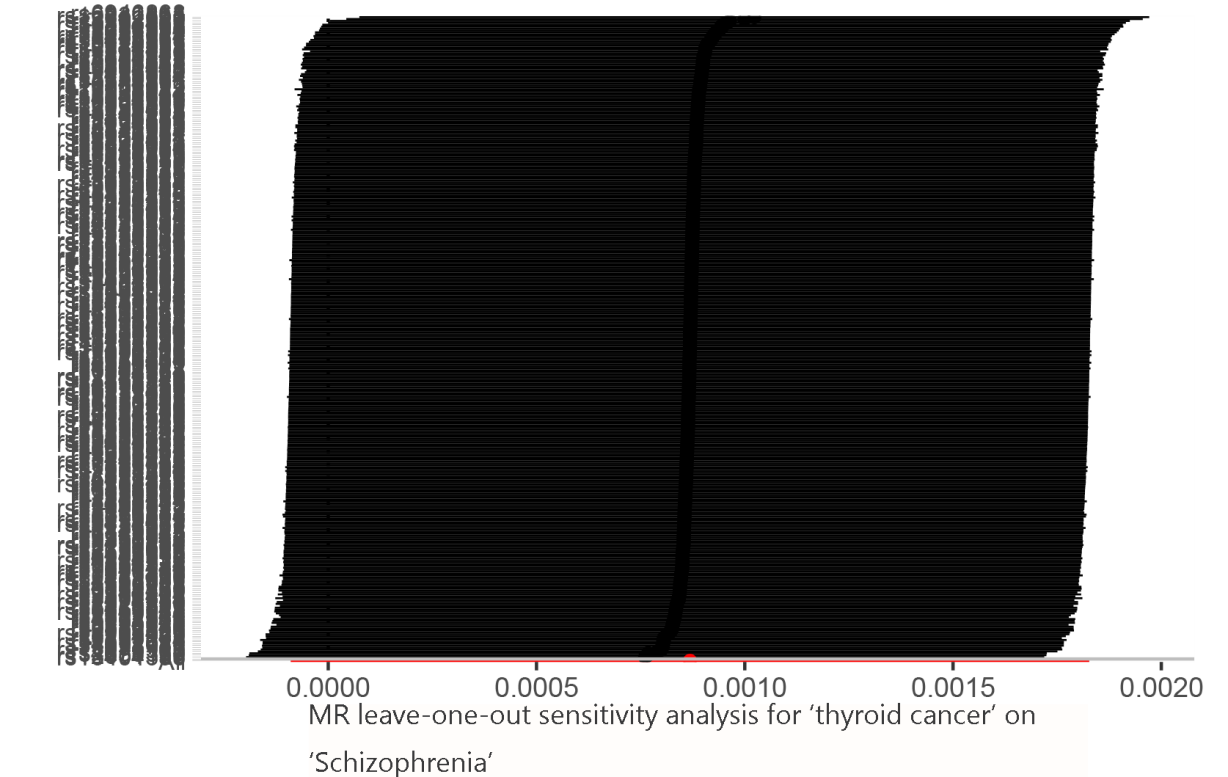


Figure S19: The leave-one-out method for the causal effect of thyroid cancer on schizophrenia.


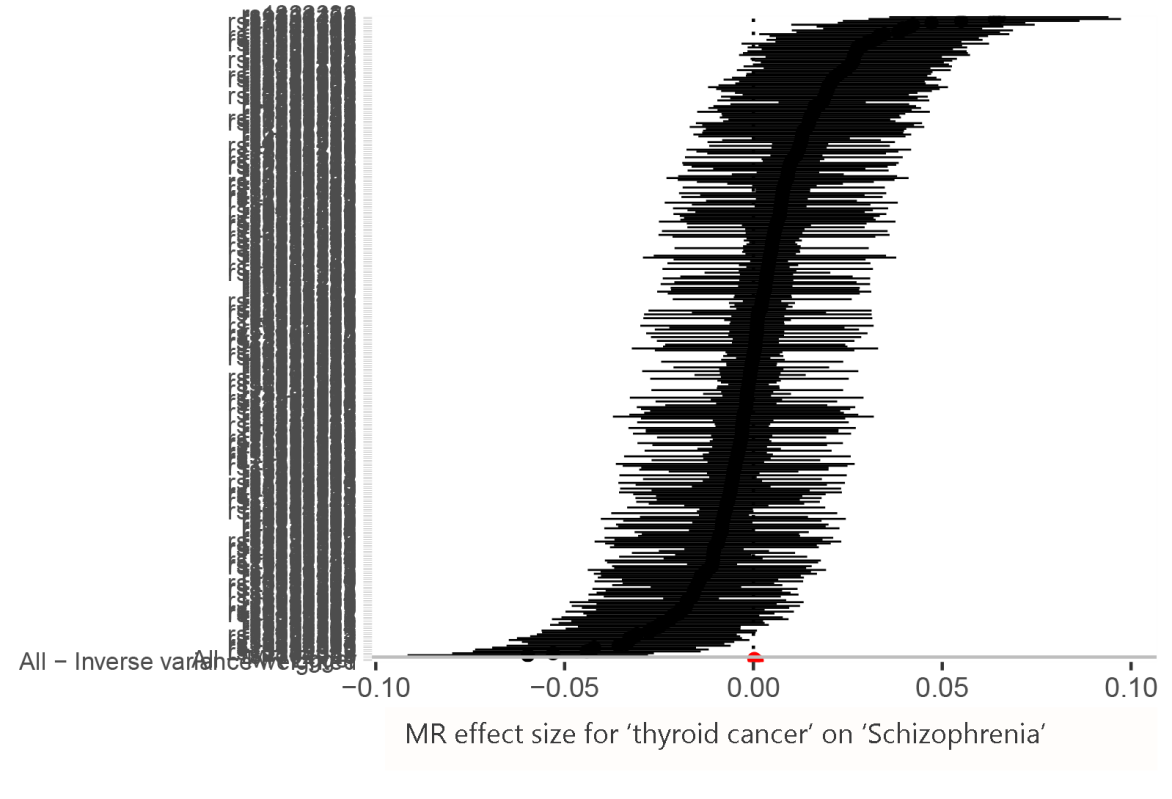


Figure S20: Forest plots of the causal effect of thyroid cancer on schizophrenia.


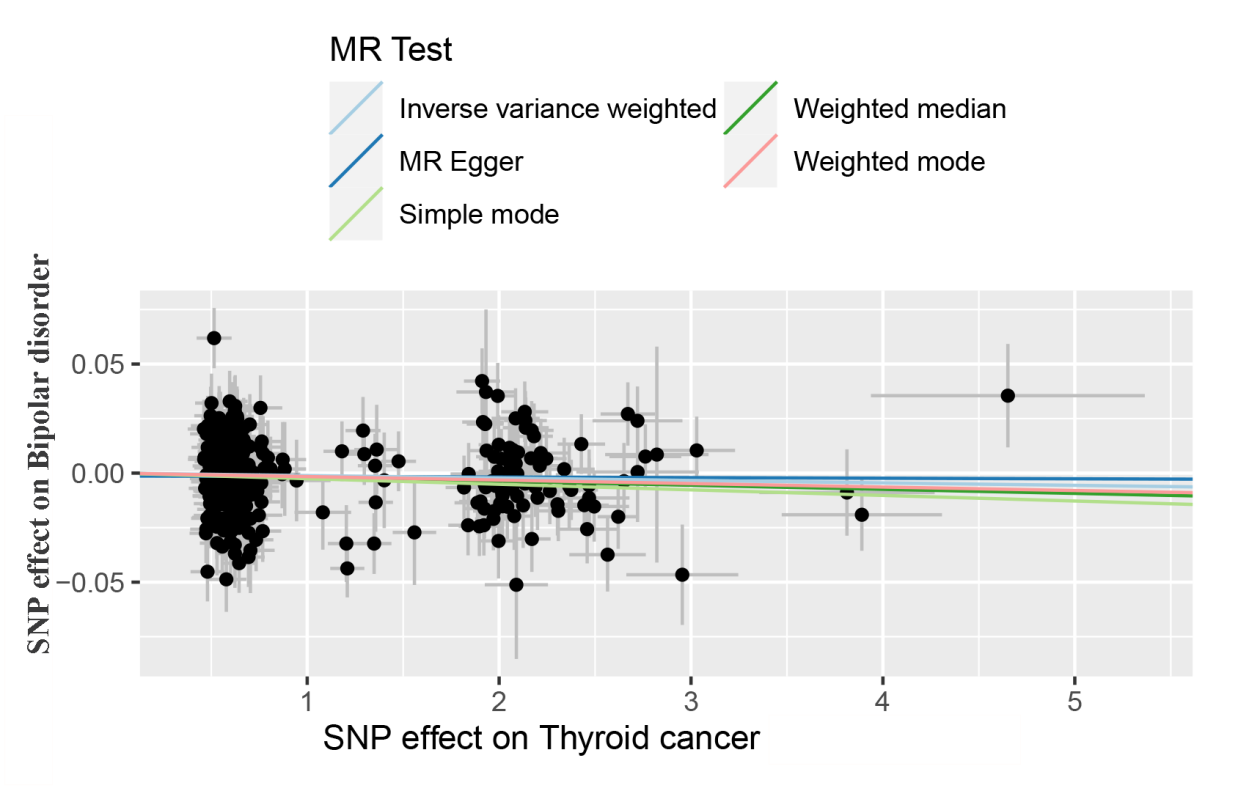


Figure S21: Scatter plot of the causal effect of thyroid cancer on bipolar disorder, with the slope of each line corresponding to estimated causal effect per method.


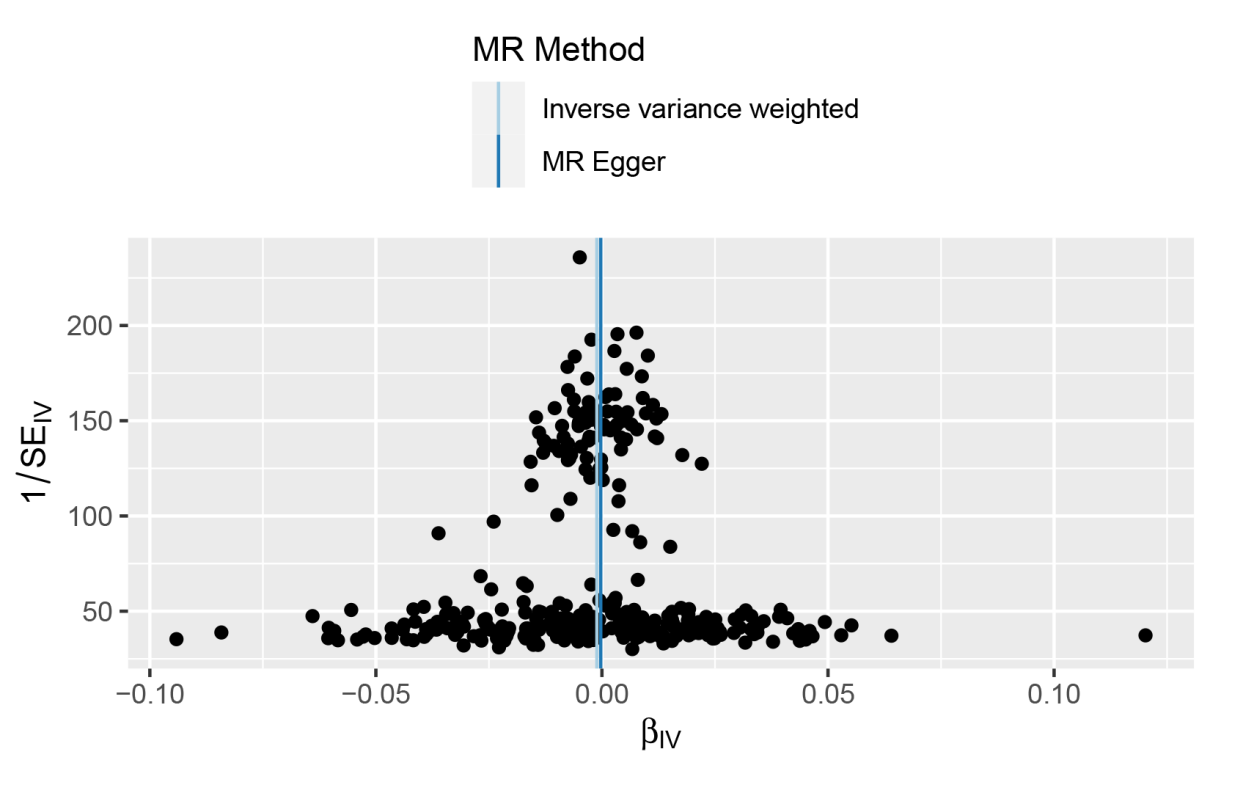


Figure S22: Funnel plot of the causal effect of thyroid cancer on bipolar disorder.


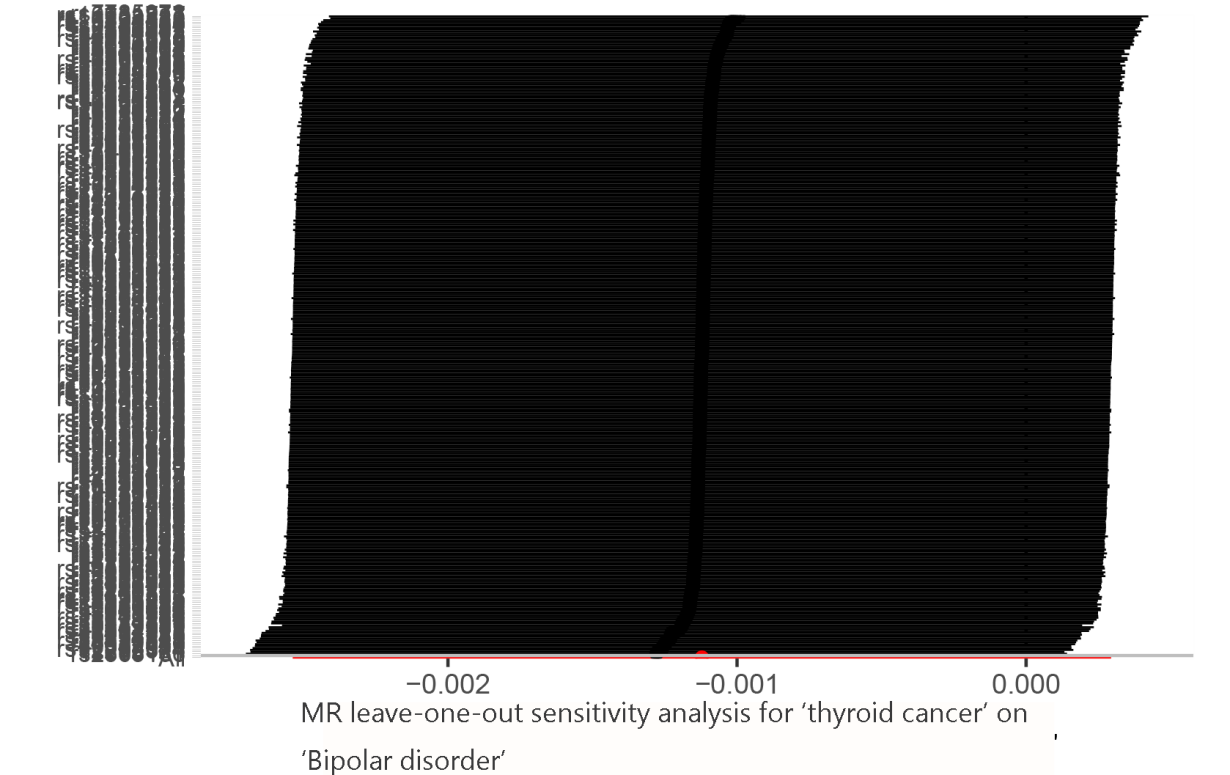


Figure S23: The leave-one-out method for the causal effect of thyroid cancer on bipolar disorder.


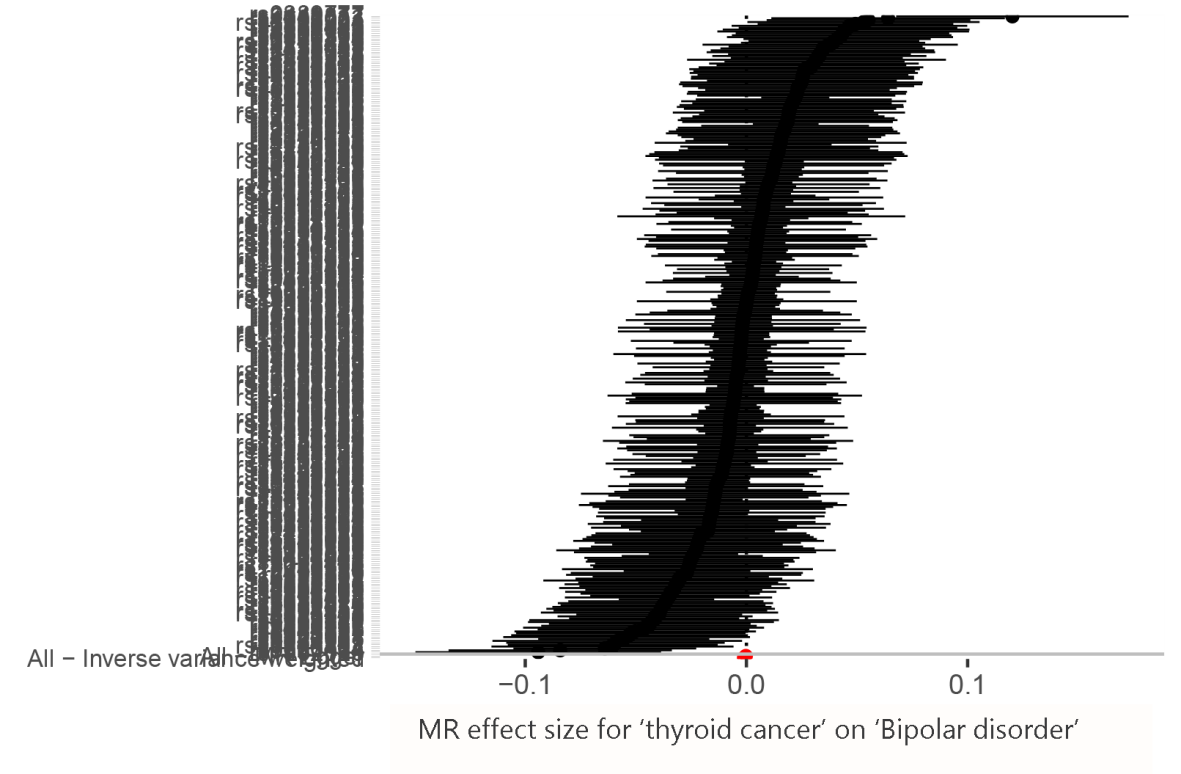


Figure S24: Forest plots of the causal effect of thyroid cancer on bipolar disorder.

Supplementary Table 1. 50 SNPs associated with major depressive disease.

| SNP | Chromosome | position | Effect allele | Other allele | effect allele frequency | Beta | Se | P | Sample size | F |
| --- | --- | --- | --- | --- | --- | --- | --- | --- | --- | --- |
| rs7551758 | 1 | 52274078 | G | T | 0.5329 | 0.0283 | 0.0043 | 5.11E-11 | 500199 | 43.31476474 |
| rs2568958 | 1 | 72765116 | A | G | 0.6042 | 0.0382 | 0.0044 | 2.90E-18 | 500199 | 75.37396694 |
| rs10913112 | 1 | 175913828 | T | C | 0.378 | -0.0262 | 0.0045 | 4.53E-09 | 500199 | 33.8982716 |
| rs17641524 | 1 | 197704717 | T | C | 0.2101 | -0.03 | 0.0053 | 1.50E-08 | 500199 | 32.03987184 |
| rs354155 | 1 | 49675276 | C | G | 0.0923 | -0.0449 | 0.0075 | 1.75E-09 | 500199 | 35.84017778 |
| rs7538938 | 1 | 67132262 | C | T | 0.5599 | 0.0251 | 0.0043 | 7.29E-09 | 500199 | 34.07301244 |
| rs4141983 | 1 | 18122009 | C | T | 0.326 | -0.0264 | 0.0046 | 9.69E-09 | 500199 | 32.93761815 |
| rs2111592 | 2 | 208049581 | A | G | 0.3141 | 0.0263 | 0.0046 | 1.35E-08 | 500199 | 32.68856333 |
| rs72948506 | 2 | 212618440 | A | G | 0.2975 | 0.0265 | 0.0047 | 1.71E-08 | 500199 | 31.7904029 |
| rs35469634 | 3 | 158171455 | G | A | 0.5774 | -0.0241 | 0.0044 | 3.28E-08 | 500199 | 30.00051653 |
| rs843812 | 3 | 61255413 | A | G | 0.4117 | 0.0248 | 0.0044 | 1.41E-08 | 500199 | 31.76859504 |
| rs9831648 | 3 | 49214303 | T | G | 0.7739 | -0.0292 | 0.0052 | 1.59E-08 | 500199 | 31.53254438 |
| rs66511648 | 3 | 117515519 | C | T | 0.284 | 0.0297 | 0.0048 | 6.03E-10 | 500199 | 38.28515625 |
| rs76954012 | 3 | 115977242 | A | T | 0.0931 | 0.0412 | 0.0074 | 2.41E-08 | 500199 | 30.99780862 |
| rs30266 | 5 | 103972357 | A | G | 0.3271 | 0.0366 | 0.0046 | 1.43E-15 | 500199 | 63.30623819 |
| rs247910 | 5 | 87630769 | G | A | 0.457 | 0.0237 | 0.0043 | 4.71E-08 | 500199 | 30.37804218 |
| rs7725715 | 5 | 164487555 | A | G | 0.5343 | 0.029 | 0.0043 | 1.61E-11 | 500199 | 45.48404543 |
| rs150186873 | 6 | 27182377 | C | A | 0.0327 | 0.0704 | 0.012 | 4.51E-09 | 500199 | 34.41777778 |
| rs2232423 | 6 | 28366151 | G | A | 0.1056 | -0.062 | 0.007 | 1.14E-18 | 500199 | 78.44897959 |
| rs9364755 | 6 | 165117329 | G | A | 0.2262 | 0.0283 | 0.0051 | 3.49E-08 | 500199 | 30.79161861 |
| rs2214123 | 6 | 67000001 | G | A | 0.6466 | -0.0261 | 0.0045 | 8.56E-09 | 500199 | 33.64 |
| rs2876520 | 6 | 142996618 | G | C | 0.4688 | 0.026 | 0.0043 | 2.24E-09 | 500199 | 36.56030287 |
| rs2522831 | 7 | 82448100 | C | T | 0.4739 | 0.024 | 0.0043 | 2.11E-08 | 500199 | 31.15197404 |
| rs4730387 | 7 | 109100414 | A | T | 0.4659 | 0.0238 | 0.0043 | 4.12E-08 | 500199 | 30.6349378 |
| rs150346963 | 7 | 117625599 | T | C | 0.4118 | 0.0283 | 0.0044 | 1.16E-10 | 500199 | 41.36828512 |
| rs3807865 | 7 | 12250402 | A | G | 0.4105 | 0.031 | 0.0044 | 1.09E-12 | 500199 | 49.63842975 |
| rs10235664 | 7 | 2086814 | C | T | 0.2529 | -0.027 | 0.0049 | 4.68E-08 | 500199 | 30.36234902 |
| rs59082935 | 7 | 38724868 | T | C | 0.1342 | 0.0363 | 0.0066 | 3.07E-08 | 500199 | 30.25 |
| rs62535714 | 9 | 37182655 | A | G | 0.1639 | 0.0339 | 0.0058 | 4.69E-09 | 500199 | 34.16200951 |
| rs1931388 | 9 | 11203149 | G | A | 0.4042 | -0.0295 | 0.0044 | 1.68E-11 | 500199 | 44.95092975 |
| rs59283172 | 9 | 25232978 | A | G | 0.1081 | -0.039 | 0.007 | 2.41E-08 | 500199 | 31.04081633 |
| rs2418449 | 9 | 119731359 | C | T | 0.281 | -0.0281 | 0.0048 | 4.25E-09 | 500199 | 34.27126736 |
| rs1021363 | 10 | 106610839 | G | A | 0.6434 | -0.03 | 0.0045 | 2.29E-11 | 500199 | 44.44444444 |
| rs198457 | 11 | 61471678 | T | C | 0.1886 | -0.0315 | 0.0056 | 1.90E-08 | 500199 | 31.640625 |
| rs4497414 | 11 | 88756779 | C | T | 0.44 | 0.0291 | 0.0044 | 2.93E-11 | 500199 | 43.74018595 |
| rs4936276 | 11 | 113365141 | C | G | 0.622 | 0.0278 | 0.0044 | 3.57E-10 | 500199 | 39.91942149 |
| rs61914045 | 12 | 52352301 | A | G | 0.2034 | 0.0309 | 0.0054 | 7.96E-09 | 500199 | 32.74382716 |
| rs9529218 | 13 | 31790053 | T | C | 0.2031 | -0.034 | 0.0054 | 2.23E-10 | 500199 | 39.64334705 |
| rs9536381 | 13 | 53860655 | T | C | 0.3259 | 0.0255 | 0.0046 | 2.62E-08 | 500199 | 30.73015123 |
| rs508502 | 13 | 80921519 | T | C | 0.2992 | -0.0264 | 0.0048 | 3.56E-08 | 500199 | 30.25 |
| rs1950829 | 14 | 42097937 | G | A | 0.5173 | -0.0297 | 0.0043 | 4.74E-12 | 500199 | 47.70632774 |
| rs754287 | 14 | 103997525 | A | T | 0.3664 | -0.0289 | 0.0045 | 1.31E-10 | 500199 | 41.24493827 |
| rs7152906 | 14 | 75125540 | C | T | 0.5196 | 0.0258 | 0.0043 | 1.87E-09 | 500199 | 36 |
| rs28541419 | 15 | 88945878 | G | C | 0.2308 | -0.0292 | 0.0052 | 1.76E-08 | 500199 | 31.53254438 |
| rs12919291 | 16 | 13800430 | C | G | 0.1884 | 0.0327 | 0.0055 | 3.09E-09 | 500199 | 35.34842975 |
| rs4799949 | 18 | 35155910 | T | C | 0.6684 | -0.0292 | 0.0046 | 1.40E-10 | 500199 | 40.29489603 |
| rs12967143 | 18 | 53099012 | C | G | 0.7012 | -0.0345 | 0.0047 | 2.53E-13 | 500199 | 53.88184699 |
| rs7241572 | 18 | 77580712 | A | G | 0.2047 | 0.0323 | 0.0054 | 2.43E-09 | 500199 | 35.77812071 |
| rs1367635 | 18 | 50861409 | C | T | 0.5148 | 0.0253 | 0.0043 | 4.35E-09 | 500199 | 34.61817198 |
| rs13037326 | 20 | 44692598 | T | C | 0.2597 | 0.031 | 0.0049 | 2.40E-10 | 500199 | 40.02498959 |

Supplementary Table 2. 217 SNPs associated with schizophrenia.

| SNP | Chromosome | position | Effect allele | Other allele | effect allele frequency | Beta | Se | P | Sample size | F |
| --- | --- | --- | --- | --- | --- | --- | --- | --- | --- | --- |
| rs3900555 | 1 | 61063225 | A | G | 0.527 | 0.0428971 | 0.0075 | 9.95E-09 | 175799 | 32.71397668 |
| rs10779702 | 1 | 8423510 | G | A | 0.712 | -0.0526968 | 0.0084 | 3.22E-10 | 175799 | 39.35590604 |
| rs56335113 | 1 | 30427639 | G | A | 0.673 | -0.0653007 | 0.0081 | 1.06E-15 | 175799 | 64.99285811 |
| rs6673880 | 1 | 2373168 | G | A | 0.563 | 0.0559991 | 0.0083 | 1.56E-11 | 175799 | 45.52038323 |
| rs10157075 | 1 | 84210463 | T | A | 0.167 | -0.0590045 | 0.0101 | 5.26E-09 | 175799 | 34.12931105 |
| rs12039854 | 1 | 97132899 | G | T | 0.188 | 0.0551005 | 0.0092 | 2.57E-09 | 175799 | 35.87033436 |
| rs7515363 | 1 | 200414959 | T | C | 0.681 | -0.0491044 | 0.0083 | 3.43E-09 | 175799 | 35.0013369 |
| rs61787564 | 1 | 29051568 | A | G | 0.148 | -0.064401 | 0.0113 | 1.34E-08 | 175799 | 32.48092099 |
| rs2970610 | 1 | 44097530 | C | T | 0.655 | -0.0639039 | 0.0078 | 2.51E-16 | 175799 | 67.12209788 |
| rs12042444 | 1 | 73988149 | T | C | 0.623 | -0.0642979 | 0.0077 | 8.53E-17 | 175799 | 69.72878975 |
| rs12126806 | 1 | 200963825 | T | C | 0.26 | -0.0497992 | 0.0086 | 6.91E-09 | 175799 | 33.53110223 |
| rs11587347 | 1 | 239198959 | G | C | 0.086 | 0.102299 | 0.0135 | 4.00E-14 | 175799 | 57.42159342 |
| rs558120 | 1 | 36537640 | C | T | 0.78 | 0.0584981 | 0.0106 | 3.32E-08 | 175799 | 30.45592474 |
| rs6588168 | 1 | 66324118 | T | C | 0.52 | 0.0458974 | 0.0075 | 1.17E-09 | 175799 | 37.45015692 |
| rs11165846 | 1 | 97819667 | G | C | 0.279 | 0.0544034 | 0.0083 | 6.45E-11 | 175799 | 42.96312863 |
| rs4950119 | 1 | 98491248 | C | A | 0.832 | 0.0906033 | 0.0102 | 8.36E-19 | 175799 | 78.90194128 |
| rs12138231 | 1 | 150115398 | A | T | 0.822 | 0.0663001 | 0.0108 | 7.37E-10 | 175799 | 37.68607047 |
| rs10559 | 1 | 153920564 | G | A | 0.301 | 0.0440982 | 0.008 | 4.02E-08 | 175799 | 30.38517568 |
| rs60124939 | 1 | 174075302 | T | C | 0.251 | -0.052896 | 0.009 | 3.76E-09 | 175799 | 34.54304711 |
| rs16851048 | 1 | 177276006 | C | T | 0.2 | 0.0680968 | 0.0091 | 8.28E-14 | 175799 | 55.99775595 |
| rs1486472 | 1 | 243803714 | A | G | 0.603 | 0.0566021 | 0.0079 | 9.16E-13 | 175799 | 51.33468554 |
| rs3770752 | 2 | 37576136 | G | A | 0.308 | -0.0592967 | 0.0084 | 1.74E-12 | 175799 | 49.8313298 |
| rs2139054 | 2 | 58156583 | C | A | 0.621 | 0.0716034 | 0.0077 | 1.04E-20 | 175799 | 86.47405788 |
| rs62152282 | 2 | 104981749 | T | A | 0.214 | 0.0580953 | 0.0094 | 6.40E-10 | 161405 | 38.19673927 |
| rs2167378 | 2 | 200045258 | T | C | 0.485 | -0.0577027 | 0.0075 | 9.90E-15 | 175799 | 59.19291711 |
| rs6715416 | 2 | 200054222 | G | A | 0.479 | -0.0600033 | 0.0075 | 1.05E-15 | 175799 | 64.00704019 |
| rs34181670 | 2 | 28019856 | C | T | 0.295 | -0.0485997 | 0.0085 | 1.13E-08 | 175799 | 32.69108429 |
| rs999494 | 2 | 73157395 | T | C | 0.191 | -0.0567016 | 0.0096 | 3.62E-09 | 175799 | 34.88575784 |
| rs62183855 | 2 | 172956449 | C | A | 0.171 | -0.061001 | 0.0102 | 1.97E-09 | 175799 | 35.76626299 |
| rs10173857 | 2 | 174951182 | T | C | 0.249 | -0.0501036 | 0.0091 | 3.84E-08 | 175799 | 30.3148259 |
| rs778371 | 2 | 233743109 | G | A | 0.256 | 0.0695001 | 0.0085 | 2.80E-16 | 175799 | 66.85486367 |
| rs17247190 | 2 | 2315801 | G | A | 0.219 | 0.0517039 | 0.0092 | 2.15E-08 | 175799 | 31.58427783 |
| rs1396728 | 2 | 22697499 | T | C | 0.455 | -0.0477992 | 0.0079 | 1.63E-09 | 175799 | 36.60893319 |
| rs7582445 | 2 | 60495874 | C | A | 0.639 | -0.0450976 | 0.0079 | 8.97E-09 | 175799 | 32.58762259 |
| rs6546857 | 2 | 73837955 | G | A | 0.231 | 0.0542028 | 0.0094 | 8.70E-09 | 175799 | 33.24970041 |
| rs6430491 | 2 | 134840967 | A | G | 0.241 | -0.0539956 | 0.0087 | 6.71E-10 | 175799 | 38.51928682 |
| rs12991836 | 2 | 145141541 | C | A | 0.379 | 0.0555021 | 0.0078 | 8.19E-13 | 175799 | 50.63252966 |
| rs60617652 | 2 | 198313300 | A | G | 0.443 | -0.0461963 | 0.0076 | 1.00E-09 | 175799 | 36.94768237 |
| rs11680723 | 2 | 200810957 | G | C | 0.159 | 0.0842951 | 0.0102 | 1.05E-16 | 175799 | 68.29742295 |
| rs7604885 | 2 | 162806408 | A | G | 0.378 | 0.0462953 | 0.008 | 5.94E-09 | 175799 | 33.48835628 |
| rs11693094 | 2 | 185601420 | T | C | 0.457 | -0.0555009 | 0.0076 | 3.20E-13 | 175799 | 53.33015756 |
| rs2033996 | 2 | 76284826 | A | T | 0.188 | 0.0519988 | 0.0095 | 4.33E-08 | 175799 | 29.95983603 |
| rs68002929 | 2 | 146419989 | A | T | 0.16 | 0.0724954 | 0.0101 | 5.52E-13 | 175799 | 51.52027273 |
| rs1881046 | 2 | 156835793 | T | G | 0.347 | -0.0443996 | 0.0079 | 1.92E-08 | 175799 | 31.5866765 |
| rs13090130 | 3 | 161777035 | A | G | 0.385 | -0.052099 | 0.0077 | 1.04E-11 | 175799 | 45.78016193 |
| rs11129383 | 3 | 30106169 | A | G | 0.284 | 0.0451961 | 0.0082 | 3.98E-08 | 175799 | 30.37905198 |
| rs4678552 | 3 | 36844522 | A | G | 0.291 | 0.0538017 | 0.0086 | 3.19E-10 | 175799 | 39.13768149 |
| rs167924 | 3 | 107379837 | G | A | 0.658 | 0.0540022 | 0.0085 | 1.84E-10 | 175799 | 40.36315024 |
| rs10935184 | 3 | 136153468 | C | T | 0.485 | -0.0608975 | 0.0078 | 7.93E-15 | 175799 | 60.95505434 |
| rs17194490 | 3 | 2547786 | T | G | 0.152 | 0.0763952 | 0.0113 | 1.53E-11 | 175799 | 45.70621492 |
| rs1399551 | 3 | 80548877 | A | T | 0.708 | 0.0542978 | 0.0082 | 4.51E-11 | 175799 | 43.84668478 |
| rs1604060 | 3 | 117772036 | G | A | 0.9059 | 0.0758017 | 0.0134 | 1.55E-08 | 175799 | 31.99987594 |
| rs525297 | 3 | 161461168 | C | A | 0.406 | -0.0459958 | 0.0079 | 5.81E-09 | 161405 | 33.89863191 |
| rs7647398 | 3 | 180733150 | T | C | 0.183 | -0.0828971 | 0.0098 | 2.46E-17 | 175799 | 71.55278205 |
| rs1866862 | 3 | 16868616 | T | A | 0.38 | 0.0499048 | 0.0077 | 8.50E-11 | 175799 | 42.00521273 |
| rs6804677 | 3 | 17856693 | C | T | 0.566 | 0.0505988 | 0.0081 | 3.84E-10 | 175799 | 39.02207836 |
| rs2710323 | 3 | 52815905 | C | T | 0.488 | -0.0716972 | 0.0074 | 3.17E-22 | 175799 | 93.87305493 |
| rs59971314 | 3 | 63914618 | C | G | 0.319 | 0.0460963 | 0.0079 | 5.88E-09 | 175799 | 34.04692956 |
| rs59518103 | 4 | 19896708 | C | T | 0.072 | -0.0780993 | 0.0143 | 4.51E-08 | 175799 | 29.82786767 |
| rs215483 | 4 | 23377121 | A | G | 0.313 | 0.0471021 | 0.008 | 4.23E-09 | 175799 | 34.66574726 |
| rs61405217 | 4 | 170322312 | T | C | 0.574 | -0.0509972 | 0.0076 | 1.43E-11 | 175799 | 45.02621897 |
| rs35734242 | 4 | 706700 | C | T | 0.42 | 0.0513986 | 0.0077 | 2.24E-11 | 175799 | 44.55753216 |
| rs13107325 | 4 | 103188709 | T | C | 0.064 | 0.156701 | 0.0165 | 1.92E-21 | 175799 | 90.19358458 |
| rs28521069 | 4 | 118852986 | T | C | 0.313 | 0.0464 | 0.0079 | 4.69E-09 | 175799 | 34.49703573 |
| rs1454606 | 4 | 33642614 | T | C | 0.185 | 0.0631954 | 0.0102 | 5.00E-10 | 175799 | 38.38579951 |
| rs28454198 | 4 | 80204001 | C | G | 0.45 | -0.0477992 | 0.0077 | 5.97E-10 | 175799 | 38.53539418 |
| rs28482891 | 4 | 47218926 | A | C | 0.335 | 0.0430954 | 0.0079 | 4.01E-08 | 175799 | 29.75826792 |
| rs7681616 | 4 | 143806223 | A | C | 0.232 | -0.0489044 | 0.0088 | 2.70E-08 | 175799 | 30.88378537 |
| rs12498839 | 4 | 176731401 | A | G | 0.066 | 0.117096 | 0.0149 | 3.24E-15 | 175799 | 61.76061086 |
| rs13159353 | 5 | 90148516 | C | G | 0.373 | -0.0432989 | 0.0078 | 2.77E-08 | 175799 | 30.81516669 |
| rs10117 | 5 | 137892170 | A | G | 0.474 | -0.052896 | 0.0079 | 1.88E-11 | 175799 | 44.83234764 |
| rs10035564 | 5 | 45252500 | G | A | 0.376 | 0.0594953 | 0.0078 | 1.70E-14 | 175799 | 58.18032088 |
| rs7701188 | 5 | 88744550 | A | G | 0.118 | -0.0746992 | 0.0118 | 2.81E-10 | 175799 | 40.07447918 |
| rs252812 | 5 | 106735490 | G | A | 0.775 | -0.052896 | 0.0091 | 5.77E-09 | 175799 | 33.78803062 |
| rs3733710 | 5 | 140167463 | T | C | 0.529 | -0.0448969 | 0.0077 | 5.52E-09 | 161405 | 33.99783487 |
| rs7709645 | 5 | 60731458 | C | G | 0.451 | 0.0649973 | 0.0078 | 6.11E-17 | 175799 | 69.43867533 |
| rs11241041 | 5 | 109183341 | T | G | 0.253 | 0.0596014 | 0.0096 | 5.35E-10 | 161405 | 38.54521356 |
| rs72723227 | 5 | 7245664 | A | G | 0.334 | -0.0503033 | 0.008 | 3.76E-10 | 175799 | 39.53784361 |
| rs11740474 | 5 | 153680747 | T | A | 0.35 | 0.0484027 | 0.0082 | 4.43E-09 | 175799 | 34.84267352 |
| rs12652777 | 5 | 155775075 | C | T | 0.554 | -0.0443996 | 0.0076 | 5.10E-09 | 175799 | 34.12957895 |
| rs9687282 | 5 | 139065988 | G | T | 0.31 | 0.0475949 | 0.0082 | 7.34E-09 | 175799 | 33.68938885 |
| rs72802887 | 5 | 152249082 | G | A | 0.241 | -0.066602 | 0.0093 | 8.53E-13 | 175799 | 51.28715926 |
| rs9470670 | 6 | 11988081 | G | T | 0.164 | 0.0555973 | 0.0099 | 1.91E-08 | 175799 | 31.53820801 |
| rs2206956 | 6 | 146739228 | A | G | 0.54 | 0.0447045 | 0.0076 | 4.04E-09 | 175799 | 34.59993629 |
| rs34555420 | 6 | 26090270 | T | G | 0.075 | -0.168696 | 0.0173 | 1.82E-22 | 161405 | 95.08617199 |
| rs6938026 | 6 | 43185733 | G | A | 0.249 | -0.0603046 | 0.0088 | 7.23E-12 | 175799 | 46.96080554 |
| rs6925079 | 6 | 64946311 | C | T | 0.357 | 0.0465048 | 0.0077 | 1.81E-09 | 175799 | 36.47657991 |
| rs80249955 | 6 | 69279811 | T | C | 0.026 | 0.154597 | 0.0283 | 4.63E-08 | 165729 | 29.84209118 |
| rs9487653 | 6 | 111822689 | G | A | 0.803 | -0.0553969 | 0.0101 | 4.40E-08 | 175799 | 30.0834872 |
| rs140365013 | 6 | 27523869 | A | G | 0.086 | -0.205696 | 0.0156 | 1.21E-39 | 175799 | 173.8611293 |
| rs9459170 | 6 | 165061435 | C | T | 0.21 | -0.0511018 | 0.0093 | 3.83E-08 | 175799 | 30.19301611 |
| rs55648125 | 6 | 50816718 | G | A | 0.09 | 0.072001 | 0.0132 | 4.42E-08 | 175799 | 29.75289257 |
| rs217310 | 6 | 84364565 | T | A | 0.424 | -0.0487044 | 0.0075 | 9.94E-11 | 175799 | 42.17099697 |
| rs12190758 | 6 | 93148341 | G | A | 0.207 | 0.0613011 | 0.0094 | 6.01E-11 | 175799 | 42.52857471 |
| rs9398171 | 6 | 108983527 | T | C | 0.687 | -0.0503033 | 0.0081 | 4.48E-10 | 175799 | 38.56762675 |
| rs58120505 | 7 | 2029867 | C | T | 0.443 | -0.0825012 | 0.0075 | 6.26E-28 | 175799 | 121.00352 |
| rs6946576 | 7 | 139018309 | A | G | 0.553 | -0.0456997 | 0.0084 | 4.94E-08 | 175799 | 29.59839257 |
| rs13233308 | 7 | 87244960 | T | C | 0.469 | -0.0458048 | 0.0075 | 1.23E-09 | 175799 | 37.29919472 |
| rs7803571 | 7 | 110993511 | T | C | 0.397 | -0.0575989 | 0.0076 | 4.49E-14 | 175799 | 57.43824933 |
| rs12671608 | 7 | 153524115 | C | T | 0.139 | 0.0598986 | 0.0109 | 4.44E-08 | 175799 | 30.19815068 |
| rs2944821 | 7 | 71795998 | C | G | 0.389 | -0.0493995 | 0.0078 | 2.51E-10 | 175799 | 40.11029915 |
| rs1593304 | 7 | 131619847 | G | A | 0.785 | 0.0631954 | 0.0097 | 5.91E-11 | 175799 | 42.44509067 |
| rs7779548 | 7 | 137074540 | A | G | 0.34 | -0.0712969 | 0.0079 | 1.82E-19 | 175799 | 81.44925412 |
| rs35274762 | 7 | 86412221 | C | T | 0.113 | -0.102304 | 0.0122 | 5.91E-17 | 175799 | 70.31784746 |
| rs56226048 | 7 | 2705107 | T | G | 0.133 | 0.0605996 | 0.0109 | 3.09E-08 | 175799 | 30.90911136 |
| rs2349487 | 7 | 8565024 | T | C | 0.328 | 0.0456042 | 0.0078 | 5.71E-09 | 175799 | 34.18381094 |
| rs79210963 | 7 | 24717969 | C | T | 0.097 | 0.0842951 | 0.0126 | 1.86E-11 | 175799 | 44.7572681 |
| rs2470951 | 7 | 104593713 | T | A | 0.405 | 0.0527997 | 0.0075 | 2.72E-12 | 175799 | 49.5610368 |
| rs211824 | 7 | 110049463 | A | G | 0.376 | -0.0481042 | 0.0077 | 4.51E-10 | 175799 | 39.02874106 |
| rs73229090 | 8 | 27442127 | A | C | 0.107 | -0.097798 | 0.014 | 2.44E-12 | 175799 | 48.79820818 |
| rs74804370 | 8 | 33998571 | C | T | 0.038 | 0.119798 | 0.0218 | 3.68E-08 | 165729 | 30.198554 |
| rs1434281 | 8 | 103671080 | A | G | 0.33 | -0.045499 | 0.008 | 1.08E-08 | 175799 | 32.34623439 |
| rs4129585 | 8 | 143312933 | C | A | 0.605 | -0.065104 | 0.0077 | 2.51E-17 | 175799 | 71.48812306 |
| rs59498392 | 8 | 38260433 | G | C | 0.231 | -0.0574006 | 0.0089 | 1.10E-10 | 175799 | 41.59612272 |
| rs11987861 | 8 | 9742206 | G | A | 0.755 | -0.0481042 | 0.0086 | 1.97E-08 | 175799 | 31.28737233 |
| rs6471815 | 8 | 60698795 | G | A | 0.615 | -0.050503 | 0.0076 | 2.71E-11 | 175799 | 44.1577737 |
| rs10957321 | 8 | 65605878 | A | G | 0.492 | 0.0490012 | 0.0074 | 3.67E-11 | 175799 | 43.84802048 |
| rs11779128 | 8 | 144929811 | T | C | 0.519 | -0.0425038 | 0.0077 | 3.98E-08 | 175799 | 30.47011325 |
| rs10103330 | 8 | 4180090 | A | T | 0.216 | 0.0628014 | 0.0093 | 1.45E-11 | 161405 | 45.60083064 |
| rs73219806 | 8 | 26279173 | A | C | 0.204 | 0.071496 | 0.0094 | 2.71E-14 | 175799 | 57.85058868 |
| rs4043663 | 8 | 89275232 | A | G | 0.756 | -0.0575045 | 0.0087 | 3.63E-11 | 175799 | 43.68830123 |
| rs10086619 | 8 | 111580570 | G | A | 0.166 | 0.0615988 | 0.0098 | 2.97E-10 | 175799 | 39.50866474 |
| rs498591 | 9 | 14509105 | T | A | 0.142 | 0.0594953 | 0.0105 | 1.36E-08 | 175799 | 32.1060383 |
| rs505061 | 9 | 22767164 | A | C | 0.489 | 0.0499994 | 0.0074 | 1.29E-11 | 175799 | 45.65266619 |
| rs10985811 | 9 | 101070487 | C | T | 0.201 | 0.0532953 | 0.0093 | 9.33E-09 | 175799 | 32.84066368 |
| rs6479494 | 9 | 96330499 | G | A | 0.155 | 0.0584981 | 0.0102 | 9.57E-09 | 175799 | 32.89146197 |
| rs13293831 | 9 | 129665139 | T | C | 0.153 | 0.0591027 | 0.0103 | 8.73E-09 | 175799 | 32.92609244 |
| rs2381411 | 9 | 36319928 | C | T | 0.411 | 0.0428031 | 0.0077 | 2.42E-08 | 175799 | 30.90074835 |
| rs4339716 | 9 | 82279449 | G | T | 0.815 | 0.0530949 | 0.0097 | 4.03E-08 | 175799 | 29.96140298 |
| rs72761691 | 9 | 134786548 | C | A | 0.137 | 0.0618967 | 0.0111 | 2.56E-08 | 175799 | 31.0948906 |
| rs61857878 | 10 | 92789488 | T | A | 0.214 | -0.0611045 | 0.0096 | 1.84E-10 | 175799 | 40.51388802 |
| rs7902292 | 10 | 123903556 | C | T | 0.749 | -0.0499038 | 0.0091 | 4.43E-08 | 175799 | 30.07353284 |
| rs113899647 | 10 | 64850074 | T | C | 0.059 | 0.0898044 | 0.0163 | 3.90E-08 | 175799 | 30.35428605 |
| rs7893279 | 10 | 18745105 | G | T | 0.108 | -0.0995014 | 0.0123 | 6.54E-16 | 175799 | 65.4407337 |
| rs12571643 | 10 | 104520277 | A | G | 0.144 | -0.113498 | 0.0113 | 1.25E-23 | 175799 | 100.8833582 |
| rs17731 | 10 | 3821561 | A | G | 0.371 | 0.0589011 | 0.0077 | 2.95E-14 | 175799 | 58.51475091 |
| rs2279311 | 10 | 21463833 | C | T | 0.266 | 0.048004 | 0.0088 | 4.12E-08 | 175799 | 29.757025 |
| rs12363019 | 11 | 24374545 | A | T | 0.427 | 0.0523992 | 0.008 | 5.91E-11 | 175799 | 42.90119001 |
| rs708228 | 11 | 57585662 | T | C | 0.283 | 0.0488017 | 0.0083 | 3.99E-09 | 175799 | 34.57114128 |
| rs3016382 | 11 | 132581442 | A | C | 0.447 | -0.0459003 | 0.0076 | 1.34E-09 | 175799 | 36.47571918 |
| rs10767735 | 11 | 28642561 | C | G | 0.458 | -0.0503984 | 0.0077 | 7.15E-11 | 175799 | 42.84025506 |
| rs2902858 | 11 | 46524013 | C | T | 0.154 | 0.0880992 | 0.0101 | 2.69E-18 | 175799 | 76.08537438 |
| rs72943392 | 11 | 81178838 | C | G | 0.236 | 0.0499048 | 0.0091 | 4.71E-08 | 175799 | 30.07473811 |
| rs77502336 | 11 | 123394636 | C | G | 0.329 | 0.054604 | 0.008 | 6.94E-12 | 175799 | 46.58745025 |
| rs11219774 | 11 | 124636373 | C | A | 0.165 | -0.0690969 | 0.0101 | 7.79E-12 | 175799 | 46.80307411 |
| rs61902811 | 11 | 113370758 | A | G | 0.29 | -0.0674998 | 0.0086 | 5.33E-15 | 175799 | 61.60388048 |
| rs11222406 | 11 | 130814093 | G | A | 0.512 | -0.0407004 | 0.0074 | 3.65E-08 | 175799 | 30.2505946 |
| rs4936216 | 11 | 133853008 | T | C | 0.187 | -0.0773036 | 0.0103 | 6.28E-14 | 175799 | 56.32808533 |
| rs3017989 | 11 | 134531650 | C | T | 0.465 | 0.0473956 | 0.0078 | 1.37E-09 | 175799 | 36.92213839 |
| rs302317 | 12 | 29921443 | A | G | 0.323 | -0.0524027 | 0.0081 | 7.87E-11 | 175799 | 41.8540309 |
| rs61920311 | 12 | 14423294 | C | A | 0.399 | -0.0459958 | 0.008 | 8.70E-09 | 175799 | 33.05646278 |
| rs578470 | 12 | 50463325 | C | T | 0.373 | 0.0456984 | 0.0079 | 8.42E-09 | 175799 | 33.46168503 |
| rs4766428 | 12 | 110723245 | T | C | 0.502 | 0.0680005 | 0.0082 | 8.11E-17 | 175799 | 68.76960143 |
| rs61937595 | 12 | 57682956 | T | C | 0.081 | -0.119799 | 0.0156 | 1.53E-14 | 175799 | 58.97353879 |
| rs2686386 | 12 | 121639657 | T | C | 0.804 | -0.0546965 | 0.0094 | 6.28E-09 | 175799 | 33.85816107 |
| rs1790135 | 12 | 123669235 | T | C | 0.719 | -0.0842033 | 0.0084 | 6.91E-24 | 175799 | 100.4846334 |
| rs4298967 | 12 | 2408194 | G | A | 0.718 | -0.0825012 | 0.0086 | 1.28E-21 | 175799 | 92.02877233 |
| rs1526803 | 12 | 75348199 | G | C | 0.317 | -0.0465018 | 0.0082 | 1.66E-08 | 175799 | 32.15968773 |
| rs61924144 | 12 | 72258821 | C | A | 0.083 | 0.0785996 | 0.0134 | 4.15E-09 | 175799 | 34.40575362 |
| rs6538539 | 12 | 95195293 | T | G | 0.532 | -0.0450976 | 0.0074 | 9.13E-10 | 175799 | 37.14013013 |
| rs1426371 | 12 | 108629780 | A | G | 0.297 | 0.0515038 | 0.0087 | 2.67E-09 | 175799 | 35.04612782 |
| rs11619756 | 13 | 44329004 | A | G | 0.474 | -0.0507977 | 0.008 | 2.04E-10 | 175799 | 40.31884883 |
| rs9569795 | 13 | 58559530 | C | T | 0.159 | 0.0635044 | 0.0102 | 5.54E-10 | 175799 | 38.76209938 |
| rs9597388 | 13 | 56928696 | A | G | 0.186 | -0.0625991 | 0.0097 | 8.98E-11 | 175799 | 41.64786184 |
| rs9545047 | 13 | 79859456 | C | A | 0.359 | -0.0551035 | 0.0079 | 4.12E-12 | 175799 | 48.65239084 |
| rs61973697 | 13 | 102022648 | A | G | 0.219 | 0.0548998 | 0.0097 | 1.57E-08 | 175799 | 32.03303263 |
| rs650520 | 13 | 38868178 | T | G | 0.377 | 0.0471964 | 0.0081 | 5.65E-09 | 161405 | 33.95061992 |
| rs12877581 | 13 | 74325499 | C | G | 0.242 | 0.0515038 | 0.0092 | 2.44E-08 | 175799 | 31.34028136 |
| rs10148671 | 14 | 29469373 | C | T | 0.566 | 0.0467038 | 0.0079 | 4.14E-09 | 175799 | 34.95024731 |
| rs12883788 | 14 | 33303540 | T | C | 0.418 | 0.0542028 | 0.0076 | 1.30E-12 | 175799 | 50.86467327 |
| rs1676062 | 14 | 84644822 | A | C | 0.373 | -0.0446961 | 0.0078 | 1.02E-08 | 175799 | 32.83598546 |
| rs10873538 | 14 | 104255569 | G | T | 0.326 | 0.0593997 | 0.0079 | 6.14E-14 | 175799 | 56.53459958 |
| rs1540840 | 14 | 99733384 | C | G | 0.394 | -0.0569 | 0.0088 | 9.65E-11 | 175799 | 41.80798037 |
| rs2190873 | 14 | 72424905 | C | T | 0.54 | -0.0628997 | 0.0074 | 2.12E-17 | 175799 | 72.24931081 |
| rs11632947 | 15 | 61852732 | T | C | 0.481 | 0.0553964 | 0.0075 | 1.64E-13 | 175799 | 54.55575347 |
| rs637137 | 15 | 78873976 | A | T | 0.287 | -0.0653007 | 0.0084 | 8.31E-15 | 175799 | 60.43341015 |
| rs56282503 | 15 | 40566759 | C | T | 0.254 | 0.055703 | 0.009 | 6.63E-10 | 175799 | 38.30647172 |
| rs2929278 | 15 | 44250313 | T | C | 0.282 | -0.0569 | 0.0087 | 6.01E-11 | 175799 | 42.77460695 |
| rs11854073 | 15 | 58972995 | A | G | 0.425 | -0.0479994 | 0.0081 | 3.66E-09 | 175799 | 35.11572017 |
| rs10906984 | 15 | 83366296 | C | A | 0.612 | -0.0491044 | 0.0076 | 8.04E-11 | 175799 | 41.74588122 |
| rs176648 | 15 | 89939528 | G | T | 0.475 | -0.0461963 | 0.0076 | 1.39E-09 | 175799 | 36.94768237 |
| rs4702 | 15 | 91426560 | A | G | 0.568 | -0.078996 | 0.0079 | 1.76E-23 | 175799 | 99.98987367 |
| rs9925915 | 16 | 29993686 | C | G | 0.433 | -0.057004 | 0.0075 | 2.51E-14 | 175799 | 57.76810695 |
| rs11862968 | 16 | 68414975 | G | C | 0.199 | 0.054604 | 0.0093 | 4.71E-09 | 175799 | 34.47331271 |
| rs12925872 | 16 | 13752356 | T | C | 0.204 | 0.067305 | 0.0091 | 1.87E-13 | 175799 | 54.70309172 |
| rs11076631 | 16 | 89877975 | G | A | 0.494 | -0.050503 | 0.0083 | 1.33E-09 | 175799 | 37.02355943 |
| rs8048039 | 16 | 4498486 | T | A | 0.641 | -0.0511968 | 0.0081 | 1.98E-10 | 175799 | 39.94989072 |
| rs9302397 | 16 | 9967827 | A | G | 0.252 | 0.0488017 | 0.0086 | 1.49E-08 | 175799 | 32.20126991 |
| rs11647188 | 16 | 82648514 | G | A | 0.468 | -0.0441987 | 0.0077 | 1.08E-08 | 175799 | 32.94864364 |
| rs12950148 | 17 | 12872802 | G | A | 0.165 | 0.0561049 | 0.01 | 1.92E-08 | 175799 | 31.47759804 |
| rs959071 | 17 | 19142226 | T | C | 0.285 | -0.075497 | 0.0109 | 4.05E-12 | 175799 | 47.97405108 |
| rs4793888 | 17 | 55737740 | A | G | 0.2 | 0.0573961 | 0.0094 | 1.12E-09 | 175799 | 37.28284626 |
| rs4293 | 17 | 61555666 | A | G | 0.549 | 0.0428031 | 0.0076 | 2.21E-08 | 175799 | 31.71927579 |
| rs11263770 | 17 | 34896877 | A | G | 0.45 | -0.0425996 | 0.0076 | 1.74E-08 | 175799 | 31.41838504 |
| rs2696466 | 17 | 44289832 | G | A | 0.412 | -0.0522983 | 0.0081 | 1.01E-10 | 175799 | 41.68742848 |
| rs12943566 | 17 | 2157774 | G | A | 0.659 | 0.0486967 | 0.0079 | 7.85E-10 | 175799 | 37.99661258 |
| rs75329315 | 17 | 1245442 | G | T | 0.097 | -0.079698 | 0.013 | 9.38E-10 | 175799 | 37.58444499 |
| rs17512480 | 18 | 53173173 | A | T | 0.03 | 0.174603 | 0.0242 | 5.60E-13 | 165729 | 52.056225 |
| rs7238071 | 18 | 77579812 | G | A | 0.299 | 0.0591027 | 0.0081 | 2.48E-13 | 175799 | 53.24080395 |
| rs35360904 | 18 | 52751297 | G | T | 0.442 | -0.0562007 | 0.0075 | 5.40E-14 | 175799 | 56.15144321 |
| rs11083369 | 18 | 27481687 | T | G | 0.273 | 0.0608015 | 0.0083 | 3.05E-13 | 175799 | 53.66268547 |
| rs715170 | 18 | 53795514 | T | C | 0.248 | -0.061001 | 0.0088 | 5.11E-12 | 175799 | 48.05167873 |
| rs72980087 | 18 | 77632194 | A | G | 0.37 | 0.0610034 | 0.0077 | 1.92E-15 | 175799 | 62.76631492 |
| rs72986630 | 19 | 11849736 | T | C | 0.058 | 0.107797 | 0.0174 | 6.31E-10 | 175799 | 38.38087333 |
| rs322124 | 19 | 11399372 | G | C | 0.219 | 0.0521041 | 0.0089 | 4.53E-09 | 175799 | 34.27392042 |
| rs8101499 | 19 | 19476984 | G | A | 0.651 | -0.0592024 | 0.0077 | 1.77E-14 | 175799 | 59.11492943 |
| rs3810450 | 19 | 36530562 | C | T | 0.067 | -0.0892006 | 0.0157 | 1.37E-08 | 175799 | 32.2802022 |
| rs7251 | 19 | 50162909 | G | C | 0.346 | -0.0582029 | 0.008 | 3.79E-13 | 175799 | 52.93089951 |
| rs758749 | 19 | 57189718 | T | C | 0.135 | 0.0617052 | 0.0109 | 1.49E-08 | 175799 | 32.04723262 |
| rs2387414 | 19 | 51034243 | C | G | 0.567 | 0.0488962 | 0.0081 | 1.40E-09 | 175799 | 36.44015203 |
| rs1006945 | 20 | 37458009 | T | G | 0.541 | 0.0601004 | 0.0074 | 3.30E-16 | 175799 | 65.96161578 |
| rs11696755 | 20 | 48105317 | C | T | 0.165 | 0.0631954 | 0.01 | 2.61E-10 | 175799 | 39.93658581 |
| rs8134737 | 21 | 16439965 | T | A | 0.468 | 0.0467038 | 0.0076 | 9.48E-10 | 175799 | 37.76393585 |
| rs229362 | 21 | 22116499 | A | G | 0.813 | -0.0531047 | 0.0095 | 2.32E-08 | 175799 | 31.24774695 |
| rs1058167 | 22 | 42538029 | G | A | 0.673 | 0.0544034 | 0.0083 | 4.89E-11 | 175799 | 42.96312863 |
| rs732381 | 22 | 40051166 | T | A | 0.405 | 0.065798 | 0.0089 | 1.17E-13 | 175799 | 54.65694741 |
| rs6010045 | 22 | 51103091 | C | T | 0.674 | 0.0458031 | 0.0081 | 1.60E-08 | 175799 | 31.97567398 |

Supplementary Table 3. 16 SNPs associated with bipolar disorder.

| SNP | Chromosome | position | Effect allele | Other allele | effect allele frequency | Beta | Se | P | Sample size | F |
| --- | --- | --- | --- | --- | --- | --- | --- | --- | --- | --- |
| rs2314398 | 2 | 97413488 | G | C | 0.319554 | -0.084102 | 0.0144 | 5.92E-09 | 51710 | 34.11046684 |
| rs9834970 | 3 | 36856030 | C | T | 0.501233 | 0.101003 | 0.0134 | 5.53E-14 | 51710 | 56.81446875 |
| rs2071044 | 3 | 52847601 | T | C | 0.467916 | -0.077702 | 0.0135 | 9.09E-09 | 51710 | 33.12812512 |
| rs11724116 | 4 | 162294038 | T | C | 0.155277 | -0.104095 | 0.0188 | 3.27E-08 | 51710 | 30.65801558 |
| rs329319 | 5 | 133906609 | G | A | 0.565703 | -0.078802 | 0.0139 | 1.54E-08 | 51710 | 32.13992653 |
| rs55648125 | 6 | 50816718 | G | A | 0.108936 | 0.117096 | 0.0215 | 4.92E-08 | 51710 | 29.66246234 |
| rs10455979 | 6 | 166995260 | G | C | 0.473478 | 0.075004 | 0.0137 | 4.60E-08 | 51710 | 29.97282762 |
| rs17150022 | 7 | 24771777 | C | T | 0.120723 | 0.113202 | 0.0204 | 2.70E-08 | 51710 | 30.79270666 |
| rs13231398 | 7 | 110197412 | C | G | 0.107064 | -0.1207 | 0.0219 | 3.36E-08 | 51710 | 30.37570109 |
| rs73496688 | 11 | 79156748 | A | T | 0.149117 | 0.108702 | 0.019 | 1.05E-08 | 51710 | 32.73164766 |
| rs174592 | 11 | 61618608 | G | A | 0.372297 | 0.0774 | 0.0141 | 3.66E-08 | 51710 | 30.1330919 |
| rs10744560 | 12 | 2387099 | T | C | 0.342297 | 0.083201 | 0.014 | 2.92E-09 | 51710 | 35.31840001 |
| rs71395455 | 15 | 85153804 | G | A | 0.31149 | -0.082096 | 0.0146 | 1.93E-08 | 51710 | 31.61828305 |
| rs884301 | 17 | 53367464 | T | C | 0.381084 | 0.080298 | 0.0138 | 5.80E-09 | 51710 | 33.85721909 |
| rs111444407 | 19 | 19358207 | T | C | 0.154691 | 0.1166 | 0.0184 | 2.40E-10 | 51710 | 40.15701796 |
| rs5758065 | 22 | 41161049 | G | C | 0.489522 | -0.074402 | 0.0135 | 3.23E-08 | 51710 | 30.37397862 |

Supplementary Table 4. 347 SNPs associated with thyroid cancer.

| SNP | Chromosome | position | Effect allele | Other allele | effect allele frequency | Beta | Se | P | Sample size | F |
| --- | --- | --- | --- | --- | --- | --- | --- | --- | --- | --- |
| rs1145012 | 12 | 55062497 | A | C | 0.0625 | -0.94469 | 0.1772 | 3.87E-08 | 1187 | 28.42177527 |
| rs17039540 | 4 | 161323274 | A | C | 0.1881 | -0.581248 | 0.1068 | 4.15E-08 | 1187 | 29.61968515 |
| rs1562058 | 4 | 55251785 | A | C | 0.3512 | -0.480296 | 0.08818 | 4.68E-08 | 1187 | 29.66728137 |
| rs711943 | 3 | 135110411 | C | A | 0.5215 | 0.461718 | 0.08438 | 4.14E-08 | 1187 | 29.94157003 |
| rs2870132 | 20 | 38569980 | A | C | 0.1934 | -0.584113 | 0.1066 | 3.40E-08 | 1187 | 30.02474548 |
| rs1414154 | 13 | 80168006 | A | C | 0.5004 | -0.463105 | 0.08447 | 3.81E-08 | 1187 | 30.05757584 |
| rs7623609 | 3 | 133887637 | T | G | 0.6773 | 0.492167 | 0.08971 | 3.67E-08 | 1187 | 30.0983902 |
| rs10513889 | 18 | 54011876 | C | A | 0.5832 | 0.468245 | 0.08528 | 3.69E-08 | 1187 | 30.14754234 |
| rs9386492 | 6 | 106368709 | A | C | 0.3493 | -0.481591 | 0.08765 | 3.58E-08 | 1187 | 30.18928891 |
| rs948018 | 11 | 82312014 | A | C | 0.7327 | 0.521887 | 0.09472 | 3.11E-08 | 1187 | 30.35774992 |
| rs17256058 | 14 | 23592491 | T | G | 0.1303 | -0.692547 | 0.1255 | 2.26E-08 | 1187 | 30.45166567 |
| rs1216502 | 11 | 100414877 | C | A | 0.8162 | 0.592578 | 0.1073 | 2.57E-08 | 1187 | 30.49942163 |
| rs1750330 | 1 | 119493411 | C | A | 0.5294 | 0.464738 | 0.08413 | 3.07E-08 | 1187 | 30.51508553 |
| rs8076927 | 17 | 60171809 | G | T | 0.5835 | 0.472567 | 0.08544 | 2.94E-08 | 1187 | 30.59174867 |
| rs2484667 | 10 | 44172133 | C | A | 0.4435 | 0.473747 | 0.08544 | 2.65E-08 | 1187 | 30.74471463 |
| rs150929 | 16 | 2328650 | T | G | 0.3651 | -0.481752 | 0.08677 | 2.58E-08 | 1187 | 30.82533874 |
| rs10777489 | 12 | 93653808 | G | T | 0.5295 | -0.475613 | 0.0856 | 2.53E-08 | 1187 | 30.87165443 |
| rs30958 | 16 | 55278599 | G | T | 0.7539 | 0.539397 | 0.09706 | 2.32E-08 | 1187 | 30.88420876 |
| rs9537154 | 13 | 56100889 | A | C | 0.2007 | -0.578569 | 0.1041 | 2.20E-08 | 1187 | 30.88935653 |
| rs17294252 | 4 | 138801097 | A | C | 0.1525 | -0.64531 | 0.1159 | 1.81E-08 | 1187 | 31.00058708 |
| rs6870385 | 5 | 27559659 | C | A | 0.6872 | 0.501536 | 0.08994 | 2.17E-08 | 1187 | 31.09556537 |
| rs3829647 | 19 | 52216563 | G | T | 0.6535 | 0.491023 | 0.08798 | 2.16E-08 | 1187 | 31.14840088 |
| rs8043171 | 15 | 92264467 | T | G | 0.1259 | -0.704006 | 0.1261 | 1.53E-08 | 1187 | 31.16897695 |
| rs2452477 | 2 | 143756755 | T | G | 0.1425 | -0.66456 | 0.119 | 1.60E-08 | 1187 | 31.18706261 |
| rs10779770 | 1 | 12606725 | C | A | 0.8724 | 0.700575 | 0.1254 | 1.48E-08 | 1187 | 31.21146816 |
| rs7815102 | 8 | 87201127 | G | T | 0.3462 | 0.503197 | 0.08974 | 1.85E-08 | 1187 | 31.44155061 |
| rs6670601 | 1 | 192758615 | C | A | 0.5611 | 0.474333 | 0.08456 | 1.86E-08 | 1187 | 31.46565175 |
| rs17647119 | 18 | 33118693 | A | C | 0.2214 | -0.565634 | 0.1007 | 1.57E-08 | 1187 | 31.55092327 |
| rs10860779 | 12 | 102127262 | A | C | 0.2764 | -0.524756 | 0.09341 | 1.67E-08 | 1187 | 31.55935232 |
| rs10952871 | 7 | 85593608 | T | G | 0.351 | -0.493805 | 0.0879 | 1.75E-08 | 1187 | 31.55972541 |
| rs929958 | 4 | 37769060 | A | C | 0.4672 | -0.474494 | 0.08412 | 1.56E-08 | 1187 | 31.81727065 |
| rs4760566 | 12 | 129200924 | C | A | 0.8155 | 0.60991 | 0.1081 | 1.25E-08 | 1187 | 31.83318698 |
| rs6540082 | 16 | 87842141 | T | G | 0.5964 | -0.491031 | 0.08694 | 1.45E-08 | 1187 | 31.89910884 |
| rs9889777 | 17 | 49336793 | C | A | 0.6923 | 0.514834 | 0.09111 | 1.40E-08 | 1187 | 31.93025209 |
| rs4269220 | 4 | 185259670 | G | T | 0.5791 | 0.486458 | 0.0857 | 1.25E-08 | 1187 | 32.22026114 |
| rs2197176 | 2 | 117377433 | C | A | 0.8258 | 0.629797 | 0.1106 | 9.15E-09 | 1187 | 32.42581654 |
| rs7869617 | 9 | 113060439 | C | A | 0.5094 | 0.47965 | 0.08421 | 1.13E-08 | 1187 | 32.44304156 |
| rs2867803 | 7 | 75284941 | A | C | 0.4428 | -0.481429 | 0.08448 | 1.11E-08 | 1187 | 32.47556268 |
| rs7781839 | 7 | 144277757 | A | C | 0.5728 | -0.489193 | 0.0858 | 1.06E-08 | 1187 | 32.50767373 |
| rs7866443 | 9 | 119179876 | A | C | 0.1795 | -0.622689 | 0.1092 | 8.72E-09 | 1187 | 32.5159997 |
| rs274012 | 7 | 137650044 | A | C | 0.5534 | -0.487966 | 0.08535 | 9.94E-09 | 1187 | 32.68677507 |
| rs17600706 | 14 | 99150918 | T | G | 0.223 | -0.570753 | 0.09982 | 8.72E-09 | 1187 | 32.69348934 |
| rs12403117 | 1 | 79174955 | A | C | 0.2722 | -0.538026 | 0.09403 | 8.96E-09 | 1187 | 32.73962152 |
| rs6533109 | 4 | 104722062 | C | A | 0.5615 | -0.492865 | 0.0858 | 8.32E-09 | 1187 | 32.99752613 |
| rs9824256 | 3 | 113815108 | C | A | 0.6534 | 0.507498 | 0.08745 | 5.78E-09 | 1187 | 33.67821325 |
| rs4336409 | 5 | 153093323 | T | G | 0.3887 | -0.500545 | 0.08621 | 5.77E-09 | 1187 | 33.71094794 |
| rs197559 | 21 | 19362689 | G | T | 0.5286 | 0.493969 | 0.08473 | 5.03E-09 | 1187 | 33.98795047 |
| rs6949292 | 7 | 16440023 | A | C | 0.2968 | -0.537341 | 0.09206 | 4.55E-09 | 1187 | 34.06889261 |
| rs1813617 | 2 | 19590019 | A | C | 0.3482 | -0.510826 | 0.0875 | 4.64E-09 | 1187 | 34.08237744 |
| rs10493096 | 1 | 42325122 | A | C | 0.1507 | -0.683197 | 0.117 | 3.40E-09 | 1187 | 34.09731469 |
| rs11098432 | 4 | 119203708 | C | A | 0.4889 | 0.493476 | 0.08449 | 4.68E-09 | 1187 | 34.11312074 |
| rs6882903 | 5 | 40765862 | C | A | 0.7175 | 0.541113 | 0.09258 | 4.30E-09 | 1187 | 34.16186583 |
| rs4766901 | 12 | 118866406 | T | G | 0.4843 | -0.496923 | 0.08493 | 4.45E-09 | 1187 | 34.23386619 |
| rs1885597 | 14 | 25043068 | G | T | 0.5337 | 0.498403 | 0.08517 | 4.42E-09 | 1187 | 34.24427698 |
| rs6440877 | 3 | 153121917 | A | C | 0.5417 | -0.501381 | 0.08547 | 4.08E-09 | 1187 | 34.41187498 |
| rs825770 | 5 | 138115219 | G | T | 0.7904 | 0.599111 | 0.1021 | 3.38E-09 | 1187 | 34.43206765 |
| rs7858672 | 9 | 2224135 | G | T | 0.6538 | 0.523911 | 0.08892 | 3.36E-09 | 1187 | 34.71491657 |
| rs4808027 | 19 | 16171361 | G | T | 0.23 | 0.614645 | 0.1043 | 2.88E-09 | 1187 | 34.72802531 |
| rs1358852 | 6 | 90120991 | C | A | 0.355 | 0.531804 | 0.0902 | 3.16E-09 | 1187 | 34.76082891 |
| rs4142879 | 2 | 176709006 | A | C | 0.2307 | -0.585011 | 0.09916 | 2.86E-09 | 1187 | 34.80607312 |
| rs1388492 | 3 | 68245477 | T | C | 0.6763 | 0.530178 | 0.08976 | 3.00E-09 | 1187 | 34.88813203 |
| rs9325969 | 22 | 49215103 | T | G | 0.4614 | -0.501536 | 0.08487 | 3.12E-09 | 1187 | 34.92173557 |
| rs2885280 | 12 | 73412222 | A | C | 0.2278 | -0.59602 | 0.1008 | 2.66E-09 | 1187 | 34.96234887 |
| rs1468696 | 3 | 45370416 | G | T | 0.5009 | 0.502031 | 0.08463 | 2.72E-09 | 1187 | 35.1894427 |
| rs12040716 | 1 | 107109564 | C | A | 0.3667 | 0.528862 | 0.08897 | 2.44E-09 | 1187 | 35.33438567 |
| rs10260654 | 7 | 50191216 | T | G | 0.356 | -0.521044 | 0.08762 | 2.42E-09 | 1187 | 35.36244628 |
| rs272620 | 5 | 63815993 | G | T | 0.3534 | 0.532978 | 0.08958 | 2.30E-09 | 1187 | 35.3994447 |
| rs9988716 | 10 | 82715404 | A | C | 0.1627 | -0.673737 | 0.113 | 1.64E-09 | 1187 | 35.54871526 |
| rs6471521 | 8 | 96112796 | C | A | 0.6766 | 0.535973 | 0.08988 | 2.12E-09 | 1187 | 35.55983173 |
| rs9406232 | 6 | 9079324 | A | C | 0.3315 | -0.529159 | 0.08864 | 2.06E-09 | 1187 | 35.63796355 |
| rs1822338 | 11 | 24359477 | C | A | 0.6276 | 0.520035 | 0.08711 | 2.10E-09 | 1187 | 35.63929632 |
| rs11036050 | 11 | 40756460 | A | C | 0.3838 | -0.515336 | 0.08632 | 2.09E-09 | 1187 | 35.64167461 |
| rs9535343 | 13 | 50370004 | T | G | 0.3593 | -0.519194 | 0.08695 | 2.06E-09 | 1187 | 35.65497672 |
| rs10494448 | 1 | 165831128 | C | A | 0.3019 | -0.552169 | 0.09228 | 1.83E-09 | 1187 | 35.80377916 |
| rs4709487 | 6 | 161487794 | T | G | 0.1348 | -0.755023 | 0.1259 | 1.11E-09 | 1187 | 35.96407569 |
| rs1459522 | 3 | 22675098 | T | G | 0.3622 | -0.523573 | 0.08729 | 1.75E-09 | 1187 | 35.9770457 |
| rs6565631 | 17 | 77866952 | C | A | 0.7751 | 0.601115 | 0.09994 | 1.39E-09 | 1187 | 36.17732409 |
| rs1481897 | 8 | 13582367 | A | C | 0.318 | -0.543693 | 0.09031 | 1.48E-09 | 1187 | 36.24397303 |
| rs6937429 | 6 | 66021416 | G | T | 0.6614 | 0.531369 | 0.08824 | 1.49E-09 | 1187 | 36.26280791 |
| rs4738050 | 8 | 70834713 | G | T | 0.6179 | 0.519026 | 0.08615 | 1.49E-09 | 1187 | 36.29674365 |
| rs4886485 | 15 | 76531520 | C | A | 0.4774 | 0.511026 | 0.08451 | 1.30E-09 | 1187 | 36.56535465 |
| rs7186875 | 16 | 55883761 | C | A | 0.7331 | 0.578391 | 0.09559 | 1.16E-09 | 1187 | 36.61155119 |
| rs9547491 | 13 | 36947826 | G | T | 0.6858 | 0.543693 | 0.0897 | 1.15E-09 | 1187 | 36.73859952 |
| rs1552706 | 8 | 127050415 | T | G | 0.7109 | 0.561943 | 0.09269 | 1.10E-09 | 1187 | 36.75519803 |
| rs2981073 | 8 | 123467576 | C | A | 0.3802 | 0.53883 | 0.08884 | 1.12E-09 | 1187 | 36.78632771 |
| rs6951443 | 7 | 66672143 | A | C | 0.5835 | -0.528273 | 0.08707 | 1.14E-09 | 1187 | 36.81118007 |
| rs4867811 | 5 | 175319561 | G | T | 0.3396 | 0.553885 | 0.09123 | 1.07E-09 | 1187 | 36.86072375 |
| rs11786147 | 8 | 84764257 | G | T | 0.4327 | 0.519984 | 0.0856 | 1.11E-09 | 1187 | 36.90051536 |
| rs1154205 | 18 | 24586952 | G | T | 0.4628 | 0.519984 | 0.0856 | 1.09E-09 | 1187 | 36.90051536 |
| rs2243497 | 13 | 78958569 | C | A | 0.6178 | 0.530858 | 0.08709 | 9.62E-10 | 1187 | 37.15524693 |
| rs2121697 | 2 | 46709607 | C | A | 0.387 | -0.530858 | 0.08663 | 7.77E-10 | 1187 | 37.55087872 |
| rs1321947 | 20 | 12871123 | A | C | 0.4494 | -0.517179 | 0.08434 | 7.73E-10 | 1187 | 37.60231392 |
| rs886856 | 20 | 52556084 | G | T | 0.2842 | -0.567572 | 0.09254 | 7.01E-10 | 1187 | 37.61689408 |
| rs9514535 | 13 | 107088897 | A | C | 0.3709 | -0.529329 | 0.08628 | 7.44E-10 | 1187 | 37.63839565 |
| rs12782349 | 10 | 54929003 | A | C | 0.3045 | -0.555649 | 0.09054 | 6.99E-10 | 1187 | 37.66344954 |
| rs461599 | 5 | 144136931 | C | A | 0.5395 | 0.519194 | 0.08459 | 7.49E-10 | 1187 | 37.67222575 |
| rs17018881 | 3 | 81439098 | A | C | 0.1366 | -0.748237 | 0.1219 | 4.72E-10 | 1187 | 37.67653446 |
| rs1868534 | 11 | 132094466 | G | T | 0.5171 | 0.520708 | 0.08434 | 5.93E-10 | 1187 | 38.11722773 |
| rs7188594 | 16 | 83214480 | A | C | 0.3056 | -0.563523 | 0.09117 | 5.25E-10 | 1187 | 38.20492778 |
| rs10744891 | 12 | 117722341 | T | G | 0.3274 | -0.557568 | 0.08994 | 4.77E-10 | 1187 | 38.43172826 |
| rs17571067 | 1 | 226004162 | A | C | 0.291 | -0.569515 | 0.09178 | 4.44E-10 | 1187 | 38.50474286 |
| rs1898422 | 2 | 142495019 | A | C | 0.1705 | -0.687762 | 0.1108 | 3.50E-10 | 1187 | 38.52980691 |
| rs2488100 | 6 | 144031193 | G | T | 0.5795 | 0.529499 | 0.08505 | 4.20E-10 | 1187 | 38.75981117 |
| rs4904753 | 14 | 91619960 | T | G | 0.2344 | -0.613228 | 0.09848 | 3.58E-10 | 1187 | 38.77464883 |
| rs1401186 | 11 | 93812665 | A | C | 0.5111 | -0.528862 | 0.08457 | 3.48E-10 | 1187 | 39.1067806 |
| rs10916258 | 1 | 228219882 | A | C | 0.1515 | -0.735011 | 0.1172 | 2.07E-10 | 1187 | 39.33077046 |
| rs12210929 | 6 | 163647830 | A | C | 0.462 | -0.531709 | 0.08478 | 3.15E-10 | 1187 | 39.33337372 |
| rs630695 | 6 | 117252759 | G | T | 0.1366 | -0.770244 | 0.1228 | 1.87E-10 | 1187 | 39.34231527 |
| rs10416371 | 19 | 45660136 | C | A | 0.4367 | 0.541161 | 0.08627 | 3.06E-10 | 1187 | 39.34897149 |
| rs9290550 | 3 | 175096546 | T | G | 0.25 | -0.603489 | 0.09614 | 2.65E-10 | 1187 | 39.40310821 |
| rs8007859 | 14 | 69704553 | T | G | 0.6104 | 0.542144 | 0.08611 | 2.65E-10 | 1187 | 39.63894919 |
| rs6430168 | 2 | 148121393 | G | T | 0.7732 | 0.626424 | 0.09948 | 2.22E-10 | 1187 | 39.65201151 |
| rs153709 | 5 | 16904555 | A | C | 0.4108 | -0.546626 | 0.08647 | 2.25E-10 | 1187 | 39.96222418 |
| rs4896219 | 6 | 137079542 | A | C | 0.4167 | -0.540941 | 0.08547 | 2.13E-10 | 1187 | 40.0564439 |
| rs10208038 | 2 | 25032151 | G | T | 0.5615 | 0.540426 | 0.08517 | 1.93E-10 | 1187 | 40.26235513 |
| rs6984781 | 8 | 13613335 | G | T | 0.2732 | 0.622725 | 0.09803 | 1.59E-10 | 1187 | 40.35288571 |
| rs11132917 | 4 | 172764187 | G | T | 0.6498 | 0.555997 | 0.0875 | 1.77E-10 | 1187 | 40.37651122 |
| rs2589810 | 5 | 38356769 | A | C | 0.5128 | -0.537078 | 0.08446 | 1.82E-10 | 1187 | 40.43640818 |
| rs4567782 | 17 | 28268800 | C | A | 0.6109 | 0.548008 | 0.08616 | 1.73E-10 | 1187 | 40.45408815 |
| rs1390241 | 3 | 65773011 | C | A | 0.5615 | 0.5449 | 0.08565 | 1.72E-10 | 1187 | 40.47425665 |
| rs1983033 | 15 | 71909490 | A | C | 0.2848 | -0.59602 | 0.09325 | 1.29E-10 | 1187 | 40.85300294 |
| rs1971100 | 9 | 35460769 | T | G | 0.1458 | -0.760784 | 0.1186 | 7.52E-11 | 1187 | 41.14843883 |
| rs6944364 | 7 | 112095782 | G | T | 0.4399 | -0.549566 | 0.08559 | 1.17E-10 | 1187 | 41.22813221 |
| rs7849585 | 9 | 139111870 | T | G | 0.2498 | -0.621943 | 0.09684 | 9.94E-11 | 1187 | 41.24692783 |
| rs10869031 | 9 | 74130286 | G | T | 0.5979 | 0.553559 | 0.08603 | 1.06E-10 | 1187 | 41.40263543 |
| rs12186611 | 5 | 132805024 | A | C | 0.2716 | -0.606236 | 0.09393 | 8.42E-11 | 1187 | 41.65573484 |
| rs1499214 | 3 | 1390985 | T | G | 0.2695 | -0.604038 | 0.09358 | 8.33E-11 | 1187 | 41.66414175 |
| rs1028400 | 1 | 208113731 | C | A | 0.6336 | 0.560016 | 0.08672 | 8.95E-11 | 1187 | 41.70252315 |
| rs4721443 | 7 | 15528256 | G | T | 0.804 | 0.685774 | 0.1056 | 5.40E-11 | 1187 | 42.17297075 |
| rs1860990 | 9 | 122960228 | T | G | 0.2041 | -0.674325 | 0.1038 | 5.51E-11 | 1187 | 42.20304773 |
| rs2368105 | 2 | 181106013 | T | G | 0.4901 | -0.54974 | 0.08459 | 7.03E-11 | 1187 | 42.23540143 |
| rs2414003 | 15 | 50318197 | T | G | 0.161 | -0.740239 | 0.1139 | 4.42E-11 | 1187 | 42.23733194 |
| rs1880256 | 2 | 36162761 | C | A | 0.7554 | 0.632805 | 0.0972 | 5.46E-11 | 1187 | 42.38452049 |
| rs7528076 | 1 | 218361784 | A | C | 0.365 | -0.568278 | 0.08718 | 5.92E-11 | 1187 | 42.49011997 |
| rs2292324 | 16 | 84028014 | G | C | 0.06173 | -4.65111 | 0.7135 | 3.28E-42 | 1187 | 42.49377037 |
| rs1827488 | 15 | 82391020 | G | T | 0.6646 | 0.581785 | 0.08911 | 5.44E-11 | 1187 | 42.62576165 |
| rs9350030 | 6 | 17122836 | G | T | 0.3271 | 0.604316 | 0.09255 | 5.19E-11 | 1187 | 42.63589064 |
| rs11592120 | 10 | 109211106 | T | G | 0.3876 | -0.563172 | 0.08591 | 4.68E-11 | 1187 | 42.97290076 |
| rs630573 | 11 | 88650737 | C | A | 0.708 | 0.604221 | 0.09198 | 3.96E-11 | 1187 | 43.15238662 |
| rs7848973 | 9 | 100588839 | G | A | 0.527 | -0.555034 | 0.08441 | 4.12E-11 | 1187 | 43.23658407 |
| rs2044241 | 5 | 112523526 | A | C | 0.6889 | -0.623261 | 0.09468 | 3.46E-11 | 1187 | 43.33346386 |
| rs4679695 | 3 | 152687097 | C | A | 0.5666 | 0.559841 | 0.085 | 3.83E-11 | 1187 | 43.38020004 |
| rs12595914 | 16 | 83628580 | T | G | 0.4533 | -0.563523 | 0.08528 | 3.32E-11 | 1187 | 43.66454201 |
| rs163192 | 5 | 68379836 | A | C | 0.4229 | -0.568278 | 0.08593 | 3.20E-11 | 1187 | 43.73529529 |
| rs1032389 | 12 | 99692723 | A | C | 0.2812 | -0.617298 | 0.09315 | 2.60E-11 | 1187 | 43.91612588 |
| rs6498272 | 16 | 12342674 | T | G | 0.6923 | 0.604404 | 0.09119 | 2.67E-11 | 1187 | 43.92990138 |
| rs12118017 | 1 | 111306729 | C | A | 0.2657 | 0.661656 | 0.09973 | 2.23E-11 | 1187 | 44.01623301 |
| rs4233904 | 2 | 12037951 | C | A | 0.7303 | 0.626049 | 0.09423 | 2.25E-11 | 1187 | 44.14060385 |
| rs4149570 | 12 | 6451590 | C | A | 0.6911 | 0.599657 | 0.09024 | 2.39E-11 | 1187 | 44.15782176 |
| rs6578080 | 8 | 141222648 | T | G | 0.4274 | -0.564226 | 0.08475 | 2.37E-11 | 1187 | 44.32275794 |
| rs546583 | 1 | 57614330 | C | A | 0.5329 | 0.562997 | 0.08451 | 2.30E-11 | 1187 | 44.38088497 |
| rs6060124 | 20 | 33536897 | A | C | 0.1651 | -0.756513 | 0.1134 | 1.36E-11 | 1187 | 44.50478237 |
| rs2844353 | 3 | 37761233 | G | T | 0.4991 | 0.565314 | 0.08457 | 2.00E-11 | 1187 | 44.68346266 |
| rs11993947 | 8 | 124971455 | G | T | 0.439 | 0.574364 | 0.08591 | 1.93E-11 | 1187 | 44.69788612 |
| rs10786634 | 10 | 103164342 | G | T | 0.5246 | 0.573233 | 0.08545 | 1.67E-11 | 1187 | 45.0026651 |
| rs9344740 | 6 | 88619412 | T | G | 0.5823 | -0.581657 | 0.08668 | 1.60E-11 | 1187 | 45.02939508 |
| rs10475268 | 5 | 492922 | T | G | 0.4441 | -0.57412 | 0.08545 | 1.55E-11 | 1187 | 45.14204385 |
| rs12715419 | 3 | 4734046 | T | G | 0.3925 | -0.579283 | 0.08599 | 1.35E-11 | 1187 | 45.38221295 |
| rs1513052 | 12 | 17387703 | C | A | 0.6588 | 0.599657 | 0.08862 | 1.06E-11 | 1187 | 45.78701422 |
| rs9655048 | 7 | 5167160 | A | C | 0.3583 | -0.594026 | 0.08777 | 1.05E-11 | 1187 | 45.80561426 |
| rs10833197 | 11 | 19953492 | A | C | 0.431 | -0.579461 | 0.08526 | 9.00E-12 | 1187 | 46.19104276 |
| rs2690106 | 6 | 25257580 | T | G | 0.3695 | -0.593121 | 0.0871 | 7.96E-12 | 1187 | 46.37140896 |
| rs10812736 | 9 | 28064690 | A | C | 0.1699 | -0.763141 | 0.1119 | 4.65E-12 | 1187 | 46.51032782 |
| rs2001823 | 2 | 241512470 | T | G | 0.5642 | -0.594431 | 0.08694 | 6.47E-12 | 1187 | 46.74806385 |
| rs6516819 | 21 | 29510893 | G | T | 0.5802 | 0.586088 | 0.08564 | 6.35E-12 | 1187 | 46.83519595 |
| rs4332846 | 19 | 41058802 | A | C | 0.5443 | -0.583332 | 0.08519 | 6.21E-12 | 1187 | 46.88720731 |
| rs513154 | 3 | 100953212 | G | T | 0.662 | 0.606786 | 0.08845 | 5.37E-12 | 1187 | 47.06254725 |
| rs1683564 | 19 | 859214 | A | C | 0.2865 | -0.639227 | 0.09313 | 4.87E-12 | 1187 | 47.11194295 |
| rs7985861 | 13 | 69598658 | A | C | 0.3475 | -0.606236 | 0.08811 | 4.71E-12 | 1187 | 47.340522 |
| rs2336409 | 8 | 809079 | C | A | 0.4862 | 0.586119 | 0.08502 | 4.60E-12 | 1187 | 47.52579591 |
| rs6584869 | 10 | 110225049 | C | A | 0.7131 | 0.640365 | 0.09282 | 3.82E-12 | 1187 | 47.59617424 |
| rs7907903 | 10 | 1765025 | T | G | 0.5648 | -0.599386 | 0.08663 | 3.77E-12 | 1187 | 47.87144764 |
| rs4768352 | 12 | 41794490 | T | G | 0.3316 | -0.621199 | 0.08952 | 3.04E-12 | 1187 | 48.15277818 |
| rs3769754 | 2 | 98989700 | C | A | 0.4738 | 0.590561 | 0.0851 | 3.24E-12 | 1187 | 48.15821778 |
| rs12433740 | 14 | 46933240 | T | G | 0.2538 | -0.669235 | 0.09594 | 2.05E-12 | 1187 | 48.65840439 |
| rs9867503 | 3 | 187221936 | C | A | 0.6952 | 0.641314 | 0.0916 | 1.85E-12 | 1187 | 49.01742513 |
| rs9516234 | 13 | 94100353 | C | A | 0.7173 | 0.654504 | 0.09342 | 1.73E-12 | 1187 | 49.08455796 |
| rs12925224 | 16 | 9668433 | G | T | 0.6688 | 0.624741 | 0.08909 | 1.78E-12 | 1187 | 49.17474301 |
| rs8026464 | 15 | 33812915 | C | A | 0.6928 | 0.645119 | 0.09177 | 1.51E-12 | 1187 | 49.41720773 |
| rs1021224 | 5 | 96837053 | C | A | 0.3819 | 0.625938 | 0.08892 | 1.50E-12 | 1187 | 49.55228978 |
| rs2236674 | 21 | 45880800 | A | C | 0.6228 | -0.63074 | 0.08952 | 1.39E-12 | 1187 | 49.64329512 |
| rs4882110 | 11 | 48558249 | T | G | 0.4655 | -0.600386 | 0.08493 | 1.27E-12 | 1187 | 49.9733962 |
| rs678476 | 1 | 5347979 | A | C | 0.321 | -0.637902 | 0.09004 | 1.02E-12 | 1187 | 50.19228356 |
| rs11688848 | 2 | 204916206 | A | C | 0.1695 | -0.79518 | 0.1122 | 6.33E-13 | 1187 | 50.22791873 |
| rs12151689 | 2 | 166656155 | C | A | 0.7209 | 0.661455 | 0.09328 | 9.14E-13 | 1187 | 50.28327229 |
| rs2722633 | 3 | 164384338 | C | A | 0.5178 | 0.603672 | 0.08513 | 1.07E-12 | 1187 | 50.28480824 |
| rs9289086 | 3 | 117425366 | C | A | 0.3285 | 0.65959 | 0.09295 | 8.94E-13 | 1187 | 50.35578113 |
| rs2911738 | 8 | 72586292 | G | T | 0.3306 | -0.635822 | 0.08945 | 8.82E-13 | 1187 | 50.52547578 |
| rs2011347 | 2 | 106907876 | G | T | 0.6493 | 0.627547 | 0.08816 | 8.29E-13 | 1187 | 50.66981895 |
| rs2184234 | 6 | 74920235 | G | T | 0.5664 | 0.612674 | 0.08597 | 8.27E-13 | 1187 | 50.78846039 |
| rs1792746 | 18 | 53803363 | A | C | 0.3795 | -0.623435 | 0.08744 | 7.84E-13 | 1187 | 50.8349104 |
| rs9578483 | 13 | 23170754 | A | C | 0.1392 | -0.872274 | 0.1218 | 2.78E-13 | 1187 | 51.28747031 |
| rs5023314 | 6 | 148358209 | A | C | 0.4588 | -0.612858 | 0.0855 | 6.15E-13 | 1187 | 51.37921797 |
| rs156166 | 3 | 6425168 | C | A | 0.5282 | 0.606052 | 0.08453 | 6.06E-13 | 1187 | 51.40413312 |
| rs11721807 | 4 | 157094509 | T | G | 0.2295 | -0.715802 | 0.09962 | 3.94E-13 | 1187 | 51.62888432 |
| rs10877902 | 12 | 63044181 | T | G | 0.3463 | 0.658556 | 0.09125 | 3.77E-13 | 1187 | 52.08584036 |
| rs1889562 | 6 | 119449385 | G | T | 0.4127 | 0.628609 | 0.08707 | 3.94E-13 | 1187 | 52.12236347 |
| rs1224751 | 6 | 58437809 | T | C | 0.4337 | -0.613597 | 0.08499 | 4.14E-13 | 1187 | 52.12316714 |
| rs7909838 | 10 | 2297365 | A | C | 0.3683 | -0.626236 | 0.08671 | 3.91E-13 | 1187 | 52.16001259 |
| rs3594 | 8 | 30535660 | A | C | 0.3292 | -0.644167 | 0.08906 | 3.47E-13 | 1187 | 52.31564253 |
| rs11927465 | 3 | 28823658 | G | T | 0.5486 | 0.614706 | 0.08493 | 3.65E-13 | 1187 | 52.3856885 |
| rs1482176 | 3 | 73327602 | T | G | 0.3825 | -0.625862 | 0.08637 | 3.33E-13 | 1187 | 52.50870747 |
| rs8019424 | 14 | 22491095 | G | T | 0.5314 | 0.61804 | 0.08528 | 3.39E-13 | 1187 | 52.52170117 |
| rs5749581 | 22 | 33624845 | A | C | 0.5209 | -0.617345 | 0.08496 | 2.90E-13 | 1187 | 52.79914169 |
| rs2720392 | 7 | 100486035 | A | C | 0.3642 | -0.634312 | 0.08724 | 2.72E-13 | 1187 | 52.86576745 |
| rs7770868 | 6 | 38464626 | A | C | 0.4509 | -0.618225 | 0.08499 | 2.79E-13 | 1187 | 52.91240089 |
| rs7998196 | 13 | 91515582 | G | T | 0.6353 | -0.657002 | 0.09026 | 2.42E-13 | 1187 | 52.9837539 |
| rs1887387 | 9 | 90879921 | T | C | 0.3552 | -0.641884 | 0.08793 | 2.15E-13 | 1187 | 53.2891698 |
| rs3745720 | 19 | 48836082 | C | A | 0.5349 | 0.617483 | 0.08453 | 2.19E-13 | 1187 | 53.36152996 |
| rs2026912 | 10 | 91212595 | A | C | 0.09948 | -1.08146 | 0.1473 | 2.78E-14 | 1187 | 53.9033092 |
| rs10787889 | 10 | 120758823 | T | G | 0.3478 | -0.650854 | 0.08824 | 1.20E-13 | 1187 | 54.40466716 |
| rs4255455 | 10 | 128608985 | C | A | 0.553 | 0.631112 | 0.08543 | 1.17E-13 | 1187 | 54.57478873 |
| rs1511954 | 15 | 57698962 | T | G | 0.3276 | -0.66068 | 0.08938 | 1.03E-13 | 1187 | 54.63885875 |
| rs2756315 | 6 | 893800 | G | T | 0.5341 | 0.632428 | 0.08548 | 1.07E-13 | 1187 | 54.73853275 |
| rs1325201 | 1 | 80417950 | T | G | 0.6027 | 0.6362 | 0.08591 | 1.00E-13 | 1187 | 54.84030878 |
| rs7294444 | 12 | 13101454 | T | G | 0.4271 | -0.63036 | 0.08486 | 8.53E-14 | 1187 | 55.17867135 |
| rs12140722 | 1 | 213091573 | C | A | 0.4797 | 0.635518 | 0.08543 | 7.86E-14 | 1187 | 55.33945768 |
| rs1370301 | 4 | 147947622 | G | T | 0.5569 | 0.643595 | 0.08547 | 3.89E-14 | 1187 | 56.7019396 |
| rs6505863 | 18 | 13974987 | C | A | 0.7157 | 0.71335 | 0.09359 | 1.52E-14 | 1187 | 58.09602743 |
| rs11638590 | 15 | 55330141 | C | A | 0.566 | -0.667829 | 0.08758 | 1.80E-14 | 1187 | 58.14611011 |
| rs6827297 | 4 | 135554935 | G | T | 0.728 | 0.721547 | 0.09454 | 1.38E-14 | 1187 | 58.25028503 |
| rs1846861 | 2 | 227359131 | C | A | 0.5473 | 0.652773 | 0.08532 | 1.51E-14 | 1187 | 58.53595014 |
| rs6919522 | 6 | 44469505 | A | C | 0.298 | -0.703804 | 0.09155 | 9.43E-15 | 1187 | 59.09990248 |
| rs4648935 | 1 | 24466035 | T | G | 0.3463 | -0.689952 | 0.08918 | 7.02E-15 | 1187 | 59.85532865 |
| rs10474588 | 5 | 78772071 | C | A | 0.5398 | 0.663588 | 0.08487 | 3.95E-15 | 1187 | 61.13482078 |
| rs1193787 | 11 | 63095411 | T | C | 0.464 | -0.665532 | 0.08469 | 2.82E-15 | 1187 | 61.75521218 |
| rs10928200 | 2 | 144578152 | T | G | 0.2569 | -0.761854 | 0.09678 | 1.84E-15 | 1187 | 61.96868333 |
| rs17137948 | 7 | 104115294 | A | C | 0.5191 | 0.666895 | 0.08455 | 2.25E-15 | 1187 | 62.21393897 |
| rs6046115 | 20 | 19449317 | T | G | 0.4625 | -0.67119 | 0.08508 | 2.24E-15 | 1187 | 62.23518606 |
| rs12469609 | 2 | 83383702 | G | T | 0.5805 | 0.681416 | 0.08627 | 2.02E-15 | 1187 | 62.38857376 |
| rs2483374 | 13 | 25533831 | C | A | 0.6016 | 0.682405 | 0.08626 | 1.80E-15 | 1187 | 62.58431352 |
| rs4148124 | 21 | 43698255 | C | A | 0.6535 | 0.700978 | 0.08845 | 1.50E-15 | 1187 | 62.80773059 |
| rs10954267 | 7 | 129933481 | A | C | 0.4948 | -0.67629 | 0.08515 | 1.42E-15 | 1187 | 63.08071785 |
| rs1467345 | 7 | 11783917 | A | G | 0.1736 | -0.882856 | 0.1109 | 5.21E-16 | 1187 | 63.37480754 |
| rs10762573 | 10 | 75798148 | C | A | 0.7425 | 0.768302 | 0.09634 | 7.78E-16 | 1187 | 63.59905219 |
| rs6828245 | 4 | 25993373 | A | C | 0.3991 | -0.69535 | 0.08646 | 6.04E-16 | 1187 | 64.68095977 |
| rs4884522 | 13 | 53053768 | A | C | 0.5282 | 0.694548 | 0.08585 | 4.20E-16 | 1187 | 65.45215213 |
| rs935990 | 11 | 133089795 | C | A | 0.6852 | 0.733761 | 0.09027 | 2.56E-16 | 1187 | 66.07274727 |
| rs6546667 | 2 | 71313262 | G | A | 0.541 | 0.694148 | 0.08459 | 1.59E-16 | 1187 | 67.33891329 |
| rs3813636 | 1 | 178489978 | G | T | 0.5227 | 0.69816 | 0.08497 | 1.44E-16 | 1187 | 67.51164488 |
| rs7301555 | 12 | 85399747 | T | G | 0.06687 | -3.81263 | 0.4569 | 2.86E-43 | 1187 | 69.63170117 |
| rs1327144 | 1 | 184051573 | C | A | 0.4602 | 0.71393 | 0.08551 | 4.51E-17 | 1187 | 69.70716989 |
| rs11743743 | 5 | 6598553 | A | C | 0.4264 | -0.726497 | 0.08588 | 1.74E-17 | 1187 | 71.5621828 |
| rs11883054 | 19 | 10157219 | G | T | 0.7254 | 0.809232 | 0.09519 | 8.53E-18 | 1187 | 72.27089656 |
| rs12524738 | 6 | 140194149 | A | G | 0.05297 | -2.72159 | 0.319 | 1.08E-28 | 1187 | 72.78871206 |
| rs8095811 | 18 | 2419068 | G | T | 0.05434 | -2.12528 | 0.2471 | 6.04E-24 | 1187 | 73.97531443 |
| rs4792390 | 17 | 13628787 | A | C | 0.3199 | -0.776964 | 0.09028 | 3.96E-18 | 1187 | 74.06596615 |
| rs950275 | 10 | 49897219 | A | C | 0.2718 | -0.869407 | 0.09534 | 2.65E-20 | 1187 | 83.15645559 |
| rs10502692 | 18 | 36040352 | T | C | 0.07807 | -3.89026 | 0.4175 | 1.82E-54 | 1187 | 86.82490081 |
| rs11006981 | 10 | 28568378 | C | A | 0.06557 | -2.36542 | 0.2435 | 5.01E-32 | 1187 | 94.36666304 |
| rs16838428 | 4 | 6200768 | T | C | 0.0774 | -2.95459 | 0.2916 | 2.19E-42 | 1187 | 102.66429 |
| rs8062459 | 16 | 88934674 | G | A | 0.122 | 2.82197 | 0.2692 | 7.65E-43 | 1187 | 109.8891145 |
| rs28433203 | 1 | 46196813 | T | G | 0.09072 | -2.72098 | 0.2341 | 1.28E-49 | 1187 | 135.097674 |
| rs7904993 | 10 | 13863721 | C | T | 0.2352 | 1.36226 | 0.1168 | 4.47E-34 | 1187 | 136.0298827 |
| rs1577026 | 13 | 59445576 | A | C | 0.09253 | -2.46876 | 0.2104 | 2.66E-46 | 1187 | 137.6785468 |
| rs638335 | 1 | 78527876 | T | G | 0.7619 | -1.40143 | 0.1176 | 1.09E-35 | 1187 | 142.0130244 |
| rs895649 | 11 | 119751585 | G | A | 0.3754 | 1.18111 | 0.0962 | 3.96E-36 | 1187 | 150.7407074 |
| rs10199888 | 2 | 216503552 | T | C | 0.2828 | -1.20397 | 0.09614 | 1.42E-37 | 1187 | 156.8278163 |
| rs26887 | 5 | 53553740 | G | A | 0.1822 | 1.93167 | 0.1541 | 3.24E-44 | 1187 | 157.1305362 |
| rs9464641 | 6 | 14038947 | G | T | 0.1779 | 2.08989 | 0.1644 | 2.32E-47 | 1187 | 161.6006969 |
| rs7735978 | 5 | 103376332 | A | C | 0.1176 | -2.56525 | 0.1992 | 5.06E-56 | 1187 | 165.8367295 |
| rs9558058 | 13 | 104012768 | T | G | 0.1105 | -2.65073 | 0.2053 | 4.13E-58 | 1187 | 166.7067128 |
| rs12441088 | 15 | 78928264 | T | G | 0.5583 | -1.21462 | 0.09031 | 1.11E-42 | 1187 | 180.8877561 |
| rs10031777 | 4 | 48498290 | C | T | 0.6152 | 1.20899 | 0.08867 | 9.30E-44 | 1187 | 185.9053981 |
| rs2566511 | 7 | 150703041 | T | C | 0.1243 | -2.49532 | 0.1819 | 3.38E-61 | 1187 | 188.1859071 |
| rs2239214 | 14 | 72888427 | G | A | 0.2728 | 1.55878 | 0.1133 | 1.55E-47 | 1187 | 189.282224 |
| rs11692610 | 2 | 203499512 | C | T | 0.1364 | -2.46899 | 0.1757 | 4.02E-63 | 1187 | 197.4672777 |
| rs4658359 | 1 | 89847372 | T | G | 0.8659 | 2.26433 | 0.1609 | 1.40E-59 | 1187 | 198.0465826 |
| rs1019024 | 8 | 1363967 | C | T | 0.4387 | 1.29088 | 0.09109 | 2.02E-47 | 1187 | 200.8308102 |
| rs7034893 | 9 | 5494438 | C | T | 0.1582 | -1.93033 | 0.1346 | 4.67E-56 | 1187 | 205.6709721 |
| rs754038 | 10 | 71300334 | C | A | 0.5172 | 1.29755 | 0.08914 | 1.05E-49 | 1187 | 211.8863328 |
| rs662393 | 11 | 74086308 | G | T | 0.8537 | 2.33904 | 0.159 | 1.21E-65 | 1187 | 216.4118556 |
| rs2640734 | 8 | 27532654 | T | C | 0.1522 | -2.21733 | 0.1503 | 1.35E-63 | 1187 | 217.6419983 |
| rs219254 | 7 | 150992226 | G | A | 0.8548 | 2.76208 | 0.1871 | 1.76E-75 | 1187 | 217.9339706 |
| rs2250057 | 9 | 135775203 | A | C | 0.407 | -1.34861 | 0.09026 | 1.63E-52 | 1187 | 223.245181 |
| rs3844222 | 16 | 29283230 | G | T | 0.5621 | 1.35363 | 0.0896 | 1.36E-53 | 1187 | 228.2358818 |
| rs6793262 | 3 | 17923381 | G | T | 0.5909 | 1.35712 | 0.08969 | 7.57E-54 | 1187 | 228.954115 |
| rs10049939 | 4 | 65252628 | G | A | 0.8437 | 3.0297 | 0.1997 | 1.39E-86 | 1187 | 230.1670355 |
| rs4548277 | 9 | 14993419 | T | G | 0.8429 | 2.30409 | 0.1487 | 4.85E-71 | 1187 | 240.0915863 |
| rs12510224 | 4 | 180702862 | G | T | 0.1798 | -2.16631 | 0.1379 | 1.41E-69 | 1187 | 246.7815437 |
| rs17381234 | 13 | 108998914 | G | A | 0.2052 | -1.88717 | 0.1192 | 1.42E-65 | 1187 | 250.6510552 |
| rs10894838 | 11 | 134410837 | T | G | 0.1618 | -2.45877 | 0.1547 | 1.43E-77 | 1187 | 252.6127017 |
| rs2677879 | 18 | 2547500 | T | G | 0.4297 | -1.47622 | 0.0908 | 2.95E-62 | 1187 | 264.3202721 |
| rs4922081 | 8 | 19536790 | A | C | 0.2111 | -2.08586 | 0.1254 | 8.21E-75 | 1187 | 276.6783893 |
| rs9297398 | 8 | 108647423 | C | A | 0.2131 | -2.01741 | 0.1195 | 1.45E-75 | 1187 | 285.005032 |
| rs4358768 | 8 | 135905576 | T | G | 0.2048 | -2.29165 | 0.1344 | 1.49E-82 | 1187 | 290.7354846 |
| rs4391665 | 1 | 115921680 | G | A | 0.8158 | 2.3758 | 0.1392 | 3.23E-85 | 1187 | 291.3005371 |
| rs1123570 | 5 | 167392547 | A | C | 0.2544 | -1.90314 | 0.111 | 4.54E-75 | 1187 | 293.9649265 |
| rs12651136 | 4 | 30625891 | A | C | 0.2302 | -2.01141 | 0.1169 | 1.12E-77 | 1187 | 296.0548551 |
| rs10949938 | 7 | 64254791 | T | G | 0.7805 | 2.06593 | 0.1197 | 2.65E-79 | 1187 | 297.8810689 |
| rs12908044 | 15 | 37186014 | A | C | 0.7752 | 2.13369 | 0.1224 | 6.63E-82 | 1187 | 303.8783839 |
| rs4962362 | 10 | 128442327 | C | A | 0.2363 | -2.09476 | 0.1193 | 6.53E-82 | 1187 | 308.3100327 |
| rs13292880 | 9 | 35817579 | A | C | 0.2652 | -1.89712 | 0.1078 | 2.64E-78 | 1187 | 309.7077573 |
| rs2679163 | 2 | 232816237 | T | G | 0.2377 | -2.07227 | 0.1175 | 3.54E-82 | 1187 | 311.0405036 |
| rs7234149 | 18 | 31285269 | C | A | 0.7336 | 1.91596 | 0.108 | 1.03E-79 | 1187 | 314.7207409 |
| rs2866442 | 4 | 78558585 | G | T | 0.7066 | 1.83822 | 0.103 | 5.43E-79 | 1187 | 318.5081316 |
| rs971732 | 2 | 50142117 | C | A | 0.2351 | -2.13877 | 0.1195 | 1.06E-85 | 1187 | 320.3261226 |
| rs576070 | 5 | 31385490 | T | G | 0.2377 | -2.01741 | 0.1127 | 2.07E-83 | 1187 | 320.4354131 |
| rs4143816 | 10 | 50899856 | T | G | 0.7455 | 1.974 | 0.1095 | 2.93E-83 | 1187 | 324.987052 |
| rs13019891 | 2 | 113829869 | T | G | 0.2479 | -2.0159 | 0.1114 | 7.99E-85 | 1187 | 327.4670353 |
| rs6054459 | 20 | 6670381 | T | G | 0.2919 | -1.92346 | 0.1053 | 1.22E-83 | 1187 | 333.6641722 |
| rs3101528 | 8 | 107658187 | G | T | 0.7392 | 2.07545 | 0.1128 | 4.04E-88 | 1187 | 338.5371635 |
| rs6946868 | 7 | 83795479 | C | A | 0.2749 | -2.01741 | 0.1086 | 3.51E-88 | 1187 | 345.0870707 |
| rs1077420 | 19 | 58968875 | C | A | 0.2679 | -2.0691 | 0.1107 | 2.70E-90 | 1187 | 349.3556084 |
| rs4961718 | 9 | 16446156 | C | A | 0.7053 | 1.92552 | 0.1027 | 8.88E-88 | 1187 | 351.5241612 |
| rs2868166 | 7 | 75406913 | A | C | 0.2195 | -2.6709 | 0.1417 | 1.64E-108 | 1187 | 355.2839231 |
| rs11708894 | 3 | 104966715 | T | G | 0.3553 | -1.81708 | 0.09591 | 8.23E-87 | 1187 | 358.938724 |
| rs7271235 | 20 | 60296529 | G | T | 0.7077 | 1.99805 | 0.1049 | 1.52E-91 | 1187 | 362.795363 |
| rs9294379 | 6 | 88152535 | A | C | 0.74 | 2.1698 | 0.1138 | 8.12E-96 | 1187 | 363.5422457 |
| rs6532146 | 4 | 90166778 | A | C | 0.2757 | -2.13707 | 0.1116 | 2.71E-95 | 1187 | 366.6984771 |
| rs163952 | 5 | 92276435 | C | A | 0.7677 | 2.44369 | 0.1274 | 2.07E-104 | 1187 | 367.9199752 |
| rs2836836 | 21 | 40389491 | T | G | 0.2611 | -2.21183 | 0.1152 | 1.50E-97 | 1187 | 368.6366667 |
| rs10124357 | 9 | 79429800 | C | T | 0.7295 | 2.05416 | 0.1069 | 1.43E-94 | 1187 | 369.2437269 |
| rs1442664 | 18 | 67207852 | T | G | 0.3339 | -1.92073 | 0.09942 | 1.08E-91 | 1187 | 373.2373711 |
| rs9869760 | 3 | 119233558 | A | C | 0.2937 | -2.0526 | 0.1062 | 8.74E-95 | 1187 | 373.559354 |
| rs1082023 | 7 | 40006195 | T | G | 0.323 | -1.96184 | 0.101 | 1.93E-93 | 1187 | 377.2979302 |
| rs7823055 | 8 | 55511676 | T | G | 0.3783 | -1.84137 | 0.09437 | 1.17E-91 | 1187 | 380.7274731 |
| rs12564912 | 1 | 165051974 | T | G | 0.2524 | -2.42838 | 0.1237 | 1.92E-107 | 1187 | 385.3841912 |
| rs10832835 | 11 | 2880009 | C | A | 0.2502 | -2.3086 | 0.1175 | 1.54E-104 | 1187 | 386.0305268 |
| rs550201 | 18 | 43341772 | C | A | 0.3326 | -1.99437 | 0.1013 | 4.39E-96 | 1187 | 387.6078596 |
| rs1365242 | 15 | 45420718 | C | A | 0.7255 | 2.20003 | 0.1105 | 1.42E-103 | 1187 | 396.399091 |
| rs4564540 | 15 | 100681868 | T | G | 0.3152 | -2.09476 | 0.1049 | 9.57E-101 | 1187 | 398.7654916 |
| rs6487825 | 12 | 29590845 | G | T | 0.6451 | 1.92758 | 0.09625 | 1.03E-97 | 1187 | 401.0729263 |
| rs11975969 | 7 | 32854040 | G | T | 0.6931 | 2.13962 | 0.1062 | 2.15E-103 | 1187 | 405.904872 |
| rs8183334 | 20 | 9184660 | G | T | 0.621 | 1.93448 | 0.09559 | 3.03E-99 | 1187 | 409.5468264 |
| rs698171 | 5 | 127156760 | C | A | 0.5923 | 1.91122 | 0.09431 | 6.96E-99 | 1187 | 410.6821937 |
| rs1001948 | 10 | 25466693 | C | A | 0.6861 | 2.08667 | 0.1023 | 1.37E-104 | 1187 | 416.0602992 |
| rs13404338 | 2 | 79450227 | G | A | 0.677 | 2.13453 | 0.1038 | 1.14E-106 | 1187 | 422.8728659 |
| rs1667822 | 3 | 163930675 | T | G | 0.4096 | -1.97041 | 0.09571 | 1.92E-102 | 1187 | 423.8367135 |
| rs2802523 | 13 | 41025396 | C | A | 0.5852 | 1.98268 | 0.09573 | 1.07E-103 | 1187 | 428.9524312 |
| rs6478287 | 9 | 119947461 | G | T | 0.6341 | 2.05338 | 0.09882 | 3.80E-106 | 1187 | 431.766513 |
| rs10797423 | 1 | 2358944 | C | A | 0.5565 | 1.97472 | 0.09458 | 4.14E-105 | 1187 | 435.9256563 |
| rs2156354 | 18 | 64180050 | G | T | 0.6064 | 2.01066 | 0.09555 | 1.57E-107 | 1187 | 442.8084436 |
| rs7516666 | 1 | 100811859 | T | G | 0.4547 | -2.00545 | 0.09526 | 4.70E-107 | 1187 | 443.202828 |
| rs4881077 | 10 | 3133541 | A | C | 0.4654 | -1.99731 | 0.09473 | 2.11E-107 | 1187 | 444.5451518 |
| rs2157787 | 16 | 72928928 | C | A | 0.6058 | 2.05885 | 0.09758 | 1.30E-108 | 1187 | 445.1719438 |
| rs1547039 | 14 | 94937886 | A | C | 0.4527 | -1.9929 | 0.0943 | 7.24E-108 | 1187 | 446.6297303 |
| rs1485354 | 12 | 30938922 | C | A | 0.6264 | 2.09395 | 0.09884 | 2.11E-110 | 1187 | 448.8147704 |
| rs2973786 | 5 | 177725209 | T | G | 0.4283 | -2.04253 | 0.09622 | 3.33E-109 | 1187 | 450.6155588 |
| rs641603 | 15 | 29741156 | T | G | 0.3199 | -2.2462 | 0.1057 | 1.53E-115 | 1187 | 451.5926566 |
| rs12196235 | 6 | 103827338 | C | A | 0.5571 | 2.06436 | 0.09556 | 3.04E-113 | 1187 | 466.6793546 |
| rs1610315 | 6 | 166662128 | A | C | 0.475 | -2.07864 | 0.0962 | 1.63E-113 | 1187 | 466.8833824 |
| rs11041733 | 11 | 8103104 | T | G | 0.4017 | -2.09883 | 0.09586 | 5.06E-117 | 1187 | 479.3797418 |
| rs738667 | 22 | 47548112 | A | C | 0.3791 | -2.18926 | 0.09913 | 9.84E-121 | 1187 | 487.7356178 |
| rs11703587 | 22 | 32322414 | C | A | 0.5074 | 2.17068 | 0.09776 | 4.20E-121 | 1187 | 493.0253202 |
| rs2567501 | 17 | 70711674 | C | A | 0.5235 | 2.18214 | 0.09786 | 9.31E-122 | 1187 | 497.2271081 |
| rs1594467 | 6 | 7920361 | G | A | 0.6349 | 2.62073 | 0.1073 | 6.18E-156 | 1187 | 596.5476187 |

Supplementary Table 5. Estimates of the causal effect of thyroid cancer on different mental disorders.

| **Exposure** | **Outcome** | **IVW (fixed-effect)** | | | | **MR‒Egger** | | | | | | **Weighted median** | | **IVW (random-effect)** | |
| --- | --- | --- | --- | --- | --- | --- | --- | --- | --- | --- | --- | --- | --- | --- | --- |
|  |  | **OR (95% CI)** | **P value** | **Cochran Q** | **P value** | **OR (95% CI)** | **P value** | **Cochran Q** | **P value** | **MR‒Egger intercept** | **P value** | **OR (95% CI)** | **P value** | **OR (95% CI)** | **P value** |
| Thyroid cancer | Major depressive disease | 1.000 (0.999-1.001) | 0.997 | 657.964 | 5.266e-24 | 0.999(0.998-1.000) | 0.259 | 654.147 | 9.717e-24 | 0.001 | 0.167 | 1.000(0.999-1.001) | 0.648 | 1.000 (0.999-1.001) | 0.997 |
| Thyroid cancer | Schizophrenia | 1.001 (1.000-1.002) | 0.076 | 644.705 | 1.498e-21 | 1.000 (0.999-1.002) | 0.776 | 643.143 | 1.573e-21 | 0.001 | 0.367 | 1.001 (0.999-1.002) | 0.310 | 1.001 (1.000-1.002) | 0.076 |
| Thyroid cancer | Bipolar disorder | 0.999 (0.997-1.000) | 0.120 | 433.291 | 0.001 | 1.000(0.997-1.002) | 0.836 | 432.836 | 0.001 | -0.001 | 0.402 | 0.998 (0.996-1.000) | 0.060 | 0.998 (0.997-1.000) | 0.120 |
